# CpG-creating mutations are costly in many human viruses

**DOI:** 10.1007/s10682-020-10039-z

**Published:** 2020-04-24

**Authors:** Victoria R. Caudill, Sarina Qin, Ryan Winstead, Jasmeen Kaur, Kaho Tisthammer, E. Geo Pineda, Caroline Solis, Sarah Cobey, Trevor Bedford, Oana Carja, Rosalind M. Eggo, Katia Koelle, Katrina Lythgoe, Roland Regoes, Scott Roy, Nicole Allen, Milo Aviles, Brittany A. Baker, William Bauer, Shannel Bermudez, Corey Carlson, Edgar Castellanos, Francisca L. Catalan, Angeline Katia Chemel, Jacob Elliot, Dwayne Evans, Natalie Fiutek, Emily Fryer, Samuel Melvin Goodfellow, Mordecai Hecht, Kellen Hopp, E. Deshawn Hopson, Amirhossein Jaberi, Christen Kinney, Derek Lao, Adrienne Le, Jacky Lo, Alejandro G. Lopez, Andrea López, Fernando G. Lorenzo, Gordon T. Luu, Andrew R. Mahoney, Rebecca L. Melton, Gabriela Do Nascimento, Anjani Pradhananga, Nicole S. Rodrigues, Annie Shieh, Jasmine Sims, Rima Singh, Hasan Sulaeman, Ricky Thu, Krystal Tran, Livia Tran, Elizabeth J. Winters, Albert Wong, Pleuni S. Pennings

**Affiliations:** 1 Department of Biology, San Francisco State University, San Francisco, CA USA; 2 Department of Biology, University of Oregon, Eugene, OR USA; 3 Quantitative Systems Biology, Univeristy of California, Merced, CA USA; 4 Vaccine and Infectious Disease Division, Fred Hutchinson Cancer Research Center, Seattle, WA USA; 5 Department of Computational Biology, School of Computer Science, Carnegie Mellon University, Pittsburgh, USA; 6 Department of Infectious Disease Epidemiology, London School of Hygiene & Tropical Medicine, London, UK; 7 Department of Biology, Emory University, Atlanta, GA USA; 8 Big Data Institute, University of Oxford, Oxford, UK; 9 Department of Neurological Surgery, University of California, San Francisco, CA USA; 10 Department of Organismic and Evolutionary Biology, Harvard University, Cambridge, MA USA; 11 Department of Plant Biology, Carnegie Institution for Science, Stanford, CA USA; 12 Health Sciences Center, University of New Mexico, Albuquerque, NM USA; 13 UCSD Biomed Sciences PhD Program, University of California, San Diego, CA USA; 14 Biochemistry, Molecular, Cellular and Developmental Biology Graduate Group, University of California, Davis, CA USA; 15 UCSF Tetrad Graduate Program, University of California, San Francisco, CA USA; 16 Department of Ecology and Evolution, University of Chicago, Chicago, IL USA; 17 Department of Environmental Systems Science, ETH Zurich, Zurich, Switzerland

**Keywords:** Mutations, Viruses, Fitness costs, CpG sites

## Abstract

Mutations can occur throughout the virus genome and may be beneficial, neutral or deleterious. We are interested in mutations that yield a C next to a G, producing CpG sites. CpG sites are rare in eukaryotic and viral genomes. For the eukaryotes, it is thought that CpG sites are rare because they are prone to mutation when methylated. In viruses, we know less about why CpG sites are rare. A previous study in HIV suggested that CpG-creating transition mutations are more costly than similar non-CpG-creating mutations. To determine if this is the case in other viruses, we analyzed the allele frequencies of CpG-creating and non-CpG-creating mutations across various strains, subtypes, and genes of viruses using existing data obtained from Genbank, HIV Databases, and Virus Pathogen Resource. Our results suggest that CpG sites are indeed costly for most viruses. By understanding the cost of CpG sites, we can obtain further insights into the evolution and adaptation of viruses.

**Electronic supplementary material:**

The online version of this article (10.1007/s10682-020-10039-z) contains supplementary material, which is available to authorized users.

## Introduction

Viruses cause a multitude of diseases such as AIDS, Dengue Fever, Polio, Hepatitis, and the flu. Due to their fast replication, large population sizes and high mutation rates, viruses are able to quickly adapt to new environments (Cuevas et al. 2015). The ability of viruses to adapt quickly is seen in drug resistance evolution in HIV and HCV, immune escape in influenza and vaccine-derived polio outbreaks. High mutation rates may also lead to a high mutational load, since a large proportion of mutations are costly to the virus. In fact, experimental work has shown that most mutations are deleterious for viruses, with a select few being neutral or beneficial (Sanjuán et al. 2004; Duffy 2018).

Fitness costs influence the fate of mutations. Mutations that suffer little or no fitness costs are likely to persist in the population, whereas mutations with high fitness costs will likely be weeded out. A detailed knowledge of mutational fitness costs (also termed selection coefficients) is important to discover new functional properties of a genome and to understand and predict the evolutionary dynamics of populations. Past studies of fitness costs have produced important practical insights into problems as diverse as drug resistance in viruses (Beerenwinkel et al. 2005), extinction in small populations (Schultz and Lynch 1997), and the effect of accumulating deleterious mutations on human health (Keightley 2012). However, studying fitness costs in natural populations is difficult. As a result, most of what we know comes from in vitro studies or phylogenetic approaches (Stern et al. 2007), neither of which can directly give detailed information about the costs of individual mutations in vivo. Costs of mutations can also be studied using within-host diversity data (Zanini et al. 2017; Theys et al. 2018). In this study we use between-host diversity to study fitness costs.

Several different types of studies found evidence that CpG sites are costly for viruses. A CpG site refers to an occurrence of a nucleotide C followed by G in the 5$^{\prime }$ to 3$^{\prime }$ direction. Studies of viral genomic sequences found that CpG sites were underrepresented in almost all small viruses tested (Karlin and Cardon 1994). Burns et al. (2009) found that CpG sites significantly decreased replicative fitness of polio viruses in vitro, while an increased GC content in itself had little to no effect on the virus’s overall fitness. Stern et al. (2017) showed that CpG sites in the polio vaccine were often mutated in vaccine-derived polio outbreaks, indicating a direct cost of CpG sites in polio *in vivo*. In 2018, a previous paper from our group (Theys et al. 2018) showed that in HIV, transition mutations resulting in CpG sites, were twice as costly as -otherwise similar- non-CpG-creating mutations, thereby revealing that CpG mutations confer a cost within the host.

It is not entirely clear why CpG sites are costly, but it is likely, at least in part, because the mammalian immune system uses CpG sites to recognize foreign genetic material (Murphy and Weaver 2016). Recently it was shown that ZAP proteins, which inhibit the proliferation of most RNA viruses, are more effective when the CpG sites were common (Takata et al. 2017; Ficarelli et al. 2019).

While (Theys et al. 2018) focused on the cost of CpG-creating mutations in HIV. Here we expanded our scope to encompass an array of human viruses, including Dengue, Influenza, Entero, Herpes, Hepatitis B and C. We focused on human viruses with a sufficient number of available sequences in Genbank, HIV Databases, or The Virus Pathogen Resource (VPR). Unlike in the Theys et al. (2018) paper, we focus on population-wide data (one sequence per patient) as opposed to within-patient data. The main assumption for this study is that when CpG-creating mutations come with a cost (either within hosts or at the transmission stage), we expect them to occur at lower frequencies in the population-wide sample compared to non-CpG-creating mutations. Since the types of mutations we consider (CpG-creating and non-CpG-creating) all occur on the same species-wide genealogy, we consider any significant differences in frequencies to be likely the result of a difference in cost. For a second analysis, we assume that the average frequency of mutations is inversely proportional to the cost of the mutations. This is likely an oversimplification, but it allows us to quantify the effect size we observe.

Depending on data availability, either individual genes or whole genomes were used. We found that CpG sites are costly in most viruses, though the effect is much stronger in some viruses (e.g., HIV, BK Polyoma) than others (e.g., HCV, Rota ). A full list of viruses can be found in Table 1.

## Methods

### Data and R scripts

Data and R scripts are available on Github:

https://github.com/Vcaudill/CpG_sites/releases/tag/v1.

**Fig. 1:**
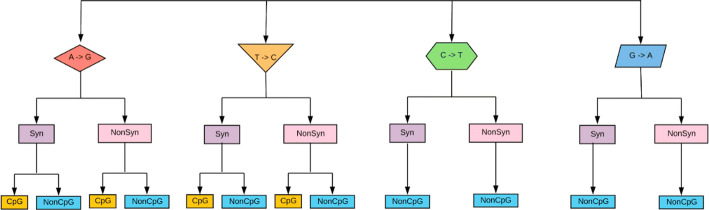
A pictorial representation of 12 transition mutation groups. Each nucleotide can mutate to one other nucleotide due to a transition. Each mutation (and site) was categorized into synonymous or non-synonymous by the resulting amino acid. For A and T, we further separated the groups into CpG-creating or non-CpG-creating mutations (Nucleotides C and G cannot form CpG sites). Most comparisons in this study are between adjacent yellow and blue mutation categories (CpG-creating vs non-CpG-creating)

**Fig. 2:**
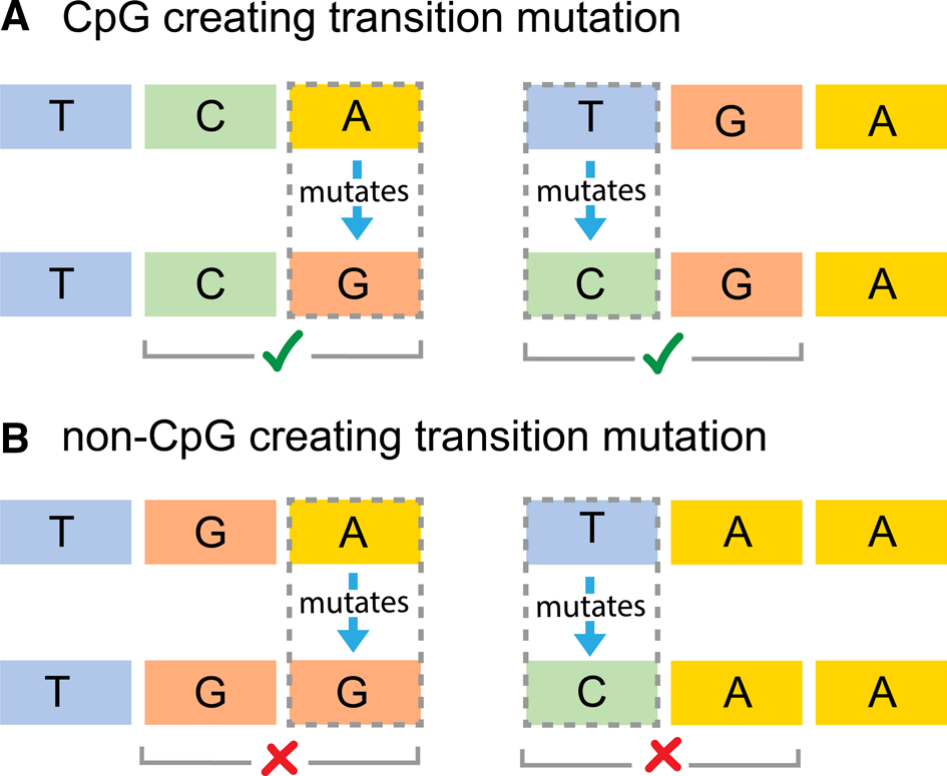
How CpG sites are created. A. There are two ways for a CpG site to be formed by a transition mutation; (1) a C precedes an A (CA) and the A mutates to a G, and (2) a T precedes a G (TG) and the T mutates to a C. B. In this study, we compare mutations that create CpG sites with similar mutations (A$\rightarrow$G and T$\rightarrow$C) that do not create CpG sites

**Fig. 3:**
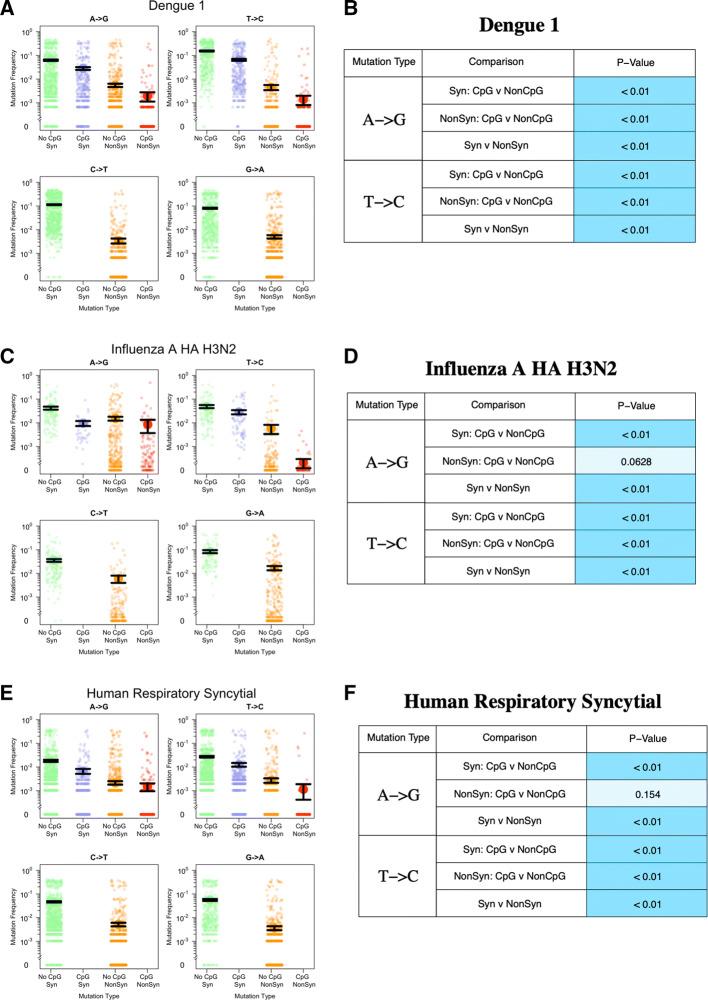
Observed transition mutation frequencies of CpG/non-CpG-creating mutations in select viral datasets (**a** the whole genome of Dengue 1 virus, **c** the HA gene of Influenza A virus H3N2, and **e** the glycoprotein gene of Human Respiratory Syncytial virus). Each figure on the left (**a**, **c**, **e**) displays transition mutation frequencies, with the mean and standard errors (black lines). The Wilcoxon test results are shown on the right (**b**, **d**, **f**). The shade of the blue color in the *p* value cell represents the significance level; darker the shade, the more significant the results are ($<~0.01$ dark blue, 0.01–0.05 medium blue, $>~0.05$ light blue)

### Data Collection

The sequences were retrieved from the NCBI Genbank, the HIV Databases (http://www.hiv.lanl.gov/), and the Virus Pathogen Resource (VPR, https://www.viprbrc.org/) using R scripts or manually (see Table 1 for data sources). We selected viral sequences from a human host, and proteins required for viral fitness (e.g. VP1, VP2, envelope protein). Dengue, Entero, and Polio sequences were all collected through the VPR, HIV sequences from the HIV Database, and HCV, Human Parainfluenza, Influenza, Human Respiratory Syncytial, Measles, Rhino, Rota, BK, Human Boca, Hepatitis B, Human Heperies, Human Papilloma, and Parvo from Genbank.

**Fig. 4:**
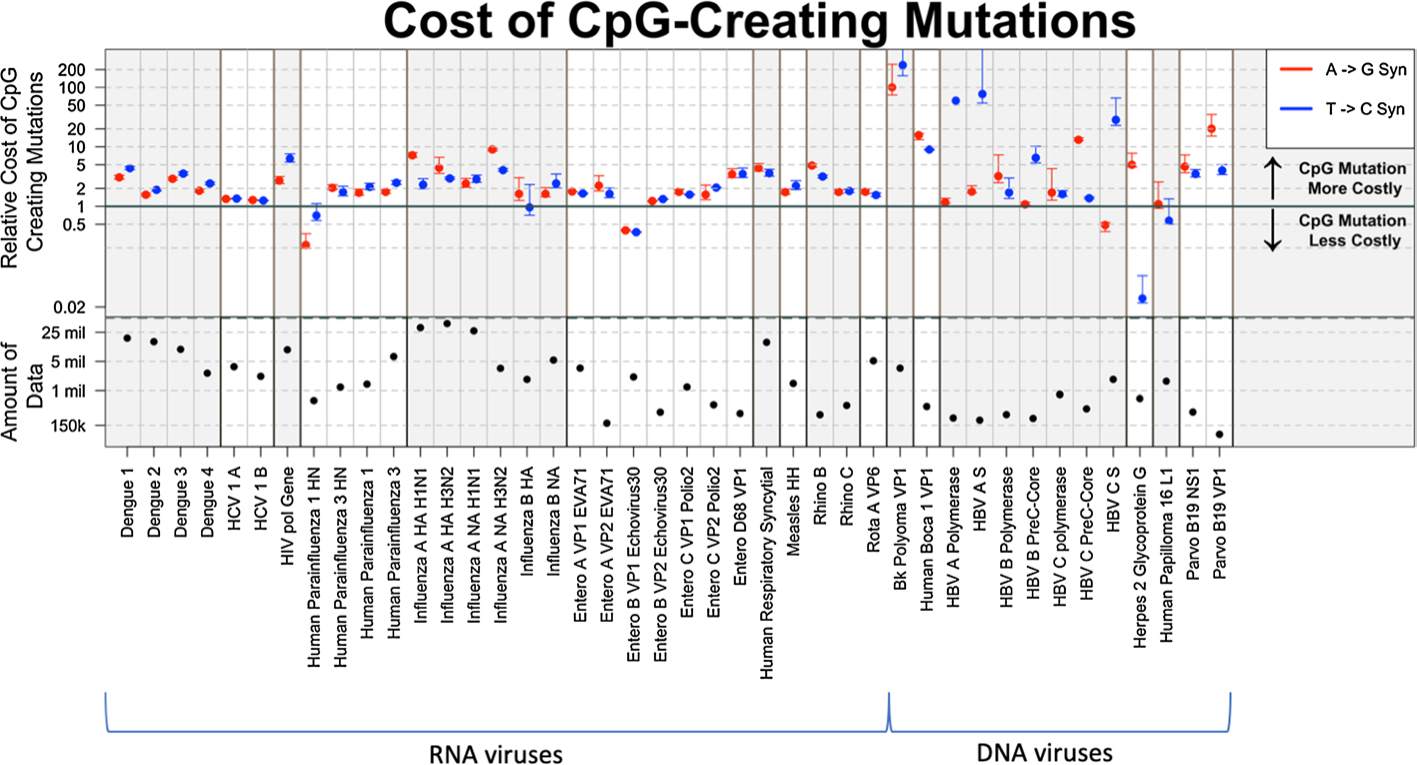
Overview of the cost associated with CpG-creating mutations. Each dot represents a ratio of the average virus mutation frequency of non-CpG-creating mutations to the average frequency of CpG-creating mutations. The bottom half of the figure depicts the total amount of data in each virus data set (the number of sequences $\times$ the number of nucleotides)

**Fig. 5:**
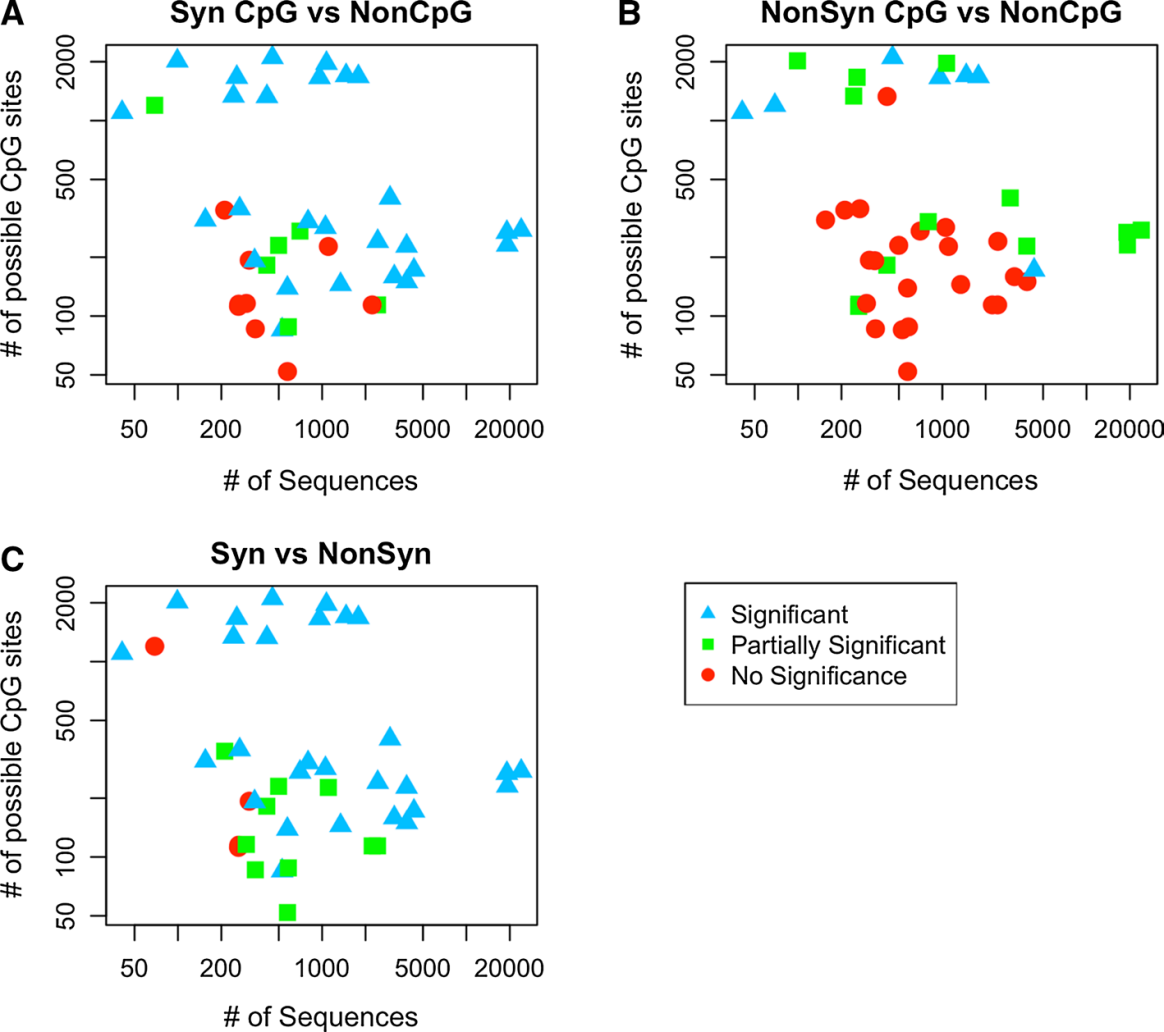
Each point represents one dataset. Its location corresponds to the amount of sequences (on the *x* axis) and the number of sites with CpG-creating mutations (on the *y* axis) for each data set. The colors and shapes represent what was found significant in each Wilcoxon test; blue triangles if both A$\rightarrow$G and T$\rightarrow$C are significant, green squares if only one was significant (partially significant) and red circles if both are not significant. We find that, in general, we are more likely to find significant effects for viruses for which we have more data (towards the top and the right)

### Further data preparation and filtering

After data collection, obtained sequences were aligned and trimmed using Geneious v.11.1.4. After checking the alignment, an online translation tool (Artimo et al. 2012) was used to identify coding regions. Once a coding region was found, the sequences were verified using NCBI BLAST. We used the program RDP4 (Martin et al. 2015) to determine if any sequences were the result of recombination. If RDP4 showed that a sequence was recombined it was cut from our analysis, unless the overall number of sequences was below 100.

Consensus sequences for each virus/protein data set were generated using R or Geneious. A custom R script was also used to identify stop codons created by mutations in the coding sequences.

**Table 1:** Information pertaining to the datasets, such as virus name, how much and where data was available, statistical results

Dataset	#Sequence	# Nucleotide	Source	A$\rightarrow$G mutations				T$\rightarrow$C mutations			
				CpG versus non-CpG creating		Syn versus Nonsyn	Ratio	CpG versus non-CpG creating		Syn versus nonsyn	Ratio
				Synonymous	Non-synonymous		Non CpG/CpG	Synonymous	Non-synonymous		Non CpG/CpG
				*p* value	*p* value	*p* value	A -> G Syn	*p* value	*p* value	*p* value	T$\rightarrow$C Syn
Dengue 1 (WG)	1783	10176	Genbank	< 0.01	< 0.01	< 0.01	3.06	< 0.01	< 0.01	< 0.01	4.37
Dengue 2 (WG)	1466	10173	Genbank	< 0.01	0.0128	< 0.01	1.57	< 0.01	0.0297	< 0.01	1.89
Dengue 3 (WG)	959	10170	Genbank	< 0.01	0.0191	< 0.01	2.90	< 0.01	< 0.01	< 0.01	3.54
Dengue 4 (WG)	256	10206	Genbank	< 0.01	0.0724	< 0.01	1.83	< 0.01	< 0.01	< 0.01	2.42
HCV 1A (WG)	414	9033	Genbank	0.021	0.385	< 0.01	1.33	0.027	0.193	< 0.01	1.35
HCV 1B (WG)	243	9033	Genbank	< 0.01	0.891	< 0.01	1.27	< 0.01	< 0.01	< 0.01	1.25
HIV pol gene	2956	3231	HIV Database	< 0.01	< 0.01	< 0.01	2.71	< 0.01	0.156	< 0.01	6.34
H Parainfluenza 1 HN	340	1728	Genbank	< 0.01	0.68	< 0.01	0.22	0.0132	0.214	< 0.01	0.70
H Parainfluenza 3 HN	702	1725	Genbank	0.271	0.45	< 0.01	2.05	< 0.01	0.782	< 0.01	1.73
H Parainfluenza 1 (WG)	99	14397	Genbank	< 0.01	0.378	< 0.01	1.69	< 0.01	< 0.01	< 0.01	2.12
H Parainfluenza 3 (WG)	452	14469	Genbank	< 0.01	0.0151	< 0.01	1.75	< 0.01	< 0.01	< 0.01	2.49
Influenza A NA H3N2	19095	1710	Genbank	< 0.01	0.224	< 0.01	7.20	< 0.01	0.126	< 0.01	2.31
Influenza A HA H1N1	24005	1701	Genbank	< 0.01	0.328	< 0.01	4.44	< 0.01	< 0.01	< 0.01	2.94
Influenza A HA H3N2	19226	1410	Genbank	< 0.01	0.0628	< 0.01	2.40	< 0.01	< 0.01	< 0.01	2.85
Influenza A NA H1N1	2428	1407	Genbank	< 0.01	0.19	< 0.01	9.03	< 0.01	< 0.01	< 0.01	4.05
Influenza B HA	1054	1755	Genbank	< 0.01	0.0946	< 0.01	1.62	< 0.01	0.162	< 0.01	0.96
Influenza B NA	3852	1398	Genbank	< 0.01	0.0413	< 0.01	1.62	< 0.01	0.199	< 0.01	2.42
Entero A VP1 EVA71	3866	894	VPR	< 0.01	0.384	< 0.01	1.77	0.0221	0.253	< 0.01	1.64
Entero A VP2 EVA71	575	294	VPR	0.0783	0.477	< 0.01	2.22	0.0847	0.545	< 0.01	1.62
Entero B VP1 Echovirus30	2419	876	VPR	0.0178	0.664	< 0.01	0.39	0.412	0.204	< 0.01	0.37
Entero B VP2 Echovirus30	413	750	VPR	< 0.01	< 0.01	< 0.01	1.23	0.128	0.196	< 0.01	1.32
Entero C VP1 Polio2	1342	906	VPR	< 0.01	0.288	< 0.01	1.75	0.0247	0.126	< 0.01	1.57
Entero C VP2 Polio2	574	813	VPR	< 0.01	0.926	< 0.01	1.57	< 0.01	0.488	< 0.01	2.07
Entero D68 VP1	528	546	VPR	0.0158	0.12	< 0.01	3.45	< 0.01	0.897	< 0.01	3.49
H Respiratory Syncytial	1071	13437	Genbank	< 0.01	0.154	< 0.01	4.40	< 0.01	< 0.01	< 0.01	3.60
Measles HH	799	1851	Genbank	< 0.01	0.0929	< 0.01	1.73	< 0.01	0.0258	< 0.01	2.20
Rhino B (WG)	41	6579	Genbank	< 0.01	< 0.01	< 0.01	4.84	< 0.01	0.0426	< 0.01	3.17
Rhino C (WG)	69	6531	Genbank	< 0.01	< 0.01	1	1.75	0.38	< 0.01	1	1.81
Rota A VP6	4331	1197	Genbank	0.0105	0.0228	< 0.01	1.75	0.0201	0.0346	< 0.01	1.55
Bk Polyoma VP1	3164	1089	Genbank	< 0.01	0.158	< 0.01	100.29	< 0.01	0.189	< 0.01	237.84
H Boca 1 VP1	211	2013	Genbank	0.195	0.0873	< 0.01	15.70	0.258	0.141	< 0.01	9.00
HBV A Polymerase	264	852	Genbank	0.389	0.347	0.192	1.17	0.15	< 0.01	0.41	59.65
HBV A S	263	837	Genbank	0.291	0.262	0.153	1.77	0.155	< 0.01	0.392	77.12
HBV B Polymerase	298	909	Genbank	0.351	0.832	0.017	3.21	0.154	0.453	< 0.01	1.70
HBV B PreC-Core	344	639	Genbank	0.764	0.199	< 0.01	1.08	0.212	0.526	< 0.01	6.53
HBV C polymerase	499	1635	Genbank	0.0176	0.218	< 0.01	1.70	0.212	0.545	< 0.01	1.60
HBV C PreC-Core	583	639	Genbank	0.047	0.876	< 0.01	13.10	0.788	0.599	< 0.01	1.37
HBV C S	2224	834	Genbank	0.646	0.153	0.0179	0.48	0.249	0.213	0.82	28.31
Herpes 2 Glycoprotein G	312	2109	Genbank	0.193	0.529	0.678	4.99	0.972	0.268	< 0.01	0.03
H Papilloma 16 L1	1104	1518	Genbank	0.0965	0.456	< 0.01	1.09	0.102	0.577	< 0.01	0.57
Parvo B19 NS1	155	2016	Genbank	< 0.01	0.805	< 0.01	4.65	< 0.01	0.22	< 0.01	3.49
Parvo B19 VP1	268	2343	Genbank	< 0.01	0.645	< 0.01	20.16	< 0.01	0.711	< 0.01	3.99

The word “Human” at the beginning of a virus name is shortened to “H”. If the whole genome was used for a virus, it is indicated by (WG)

**Table 2:** The number of data sets (out of 42) for which the Wilcoxon test was significant (percentages in parentheses) indicating that non-CpG-creating mutations were observed at higher frequencies than, otherwise similar, CpG-creating mutations

Summary of comparisons		
Comparisons	A$\rightarrow$G	T$\rightarrow$C
Synonymous: CpG versus non-CpG	32 (76.2%)	28 (66.7%)
Non-synonymous: CpG versus non-CpG	10 (23.8%)	17 (40.5%)
Synonymous versus non-synonymous	38 (90.5%)	38 (90.5%)

Accurate estimation of mutation frequencies requires sufficient data points. Therefore, we calculated data points as the number of sequences multiplied by the number of nucleotides, and removed data sets that had less than 60,000 data points. We were able to collect sufficient data for 42 data sets.

### Data analysis

For each of the 42 data sets, the consensus sequence was translated to create a wild type protein sequence. For each nucleotide, we determined whether a transition mutation would change the amino acid and/or create a CpG site. We determined whether the transition mutation was synonymous, non-synonymous or nonsense by comparing the wild type amino acid to the mutated amino acid. We calculated the frequency of the transition mutation for each nucleotide in the data set by dividing the number of observed transition mutations by the sum of the number of transition mutations and the wild type nucleotide.

### Statistical analysis

To determine if CpG sites were costly to viruses, the data were separated into groups. First, the sites were split into four categories; each represented a consensus nucleotide and its transition mutated form (Adenine to Guanine (A$\rightarrow$G), Thymine to Cytosine (T$\rightarrow$C), Cytosine to Thymine (C$\rightarrow$T), or Guanine to Adenine (G$\rightarrow$A)). The nucleotides were then sectioned into groups of synonymous and non-synonymous, and further by CpG-creating or non-CpG-creating mutations (Fig. 1). A Wilcoxon rank-sum test was performed to determine if the mutation frequencies differed between groups of synonymous versus non-synonymous, and CpG versus non-CpG-creating mutations (Fig. 3). To calculate a “cost ratio” of CpG-creating transition mutations, we divided the mean mutation frequency of non-CpG-creating mutations by the mean mutation frequency of CpG-creating mutations of the same type (Fig. 4).

### Phylogenetic approach

For each dataset we used PhyML (Guindon et al. 2010) to create unrooted trees from 200 randomly selected sequences. From the PhyML tree output we rooted the tree using the midpoint rooting method. Once rooted we used PAML (Yang 2007) to construct the ancestral sequences. Using these ancestral sequences, we repeated the “cost ratio” analysis (see supplementary figure S2).

### Simulations

Using the SLIM simulation framework (Haller and Messer 2019), we simulated viral genomes in 200 hosts. We simulated a genomic region of 10,000 base pairs and a within-host population size of 5000, the first half of the genome (0–4999) is set to have only non-CpG-creating mutations with a cost of 0.01. The second half of the genome (5000–10,000) is set to have only non-CpG-creating mutations with a cost of 0.005. The mutation rate is set to $10^{-5}$. Each simulation in a host starts with a population that consists of wildtype sequences only. The simulation will run through 1000 generations, after which a sample of 1 sequence is taken. When the simulation is run 200 times, we have 200 sequences to analyze. The average frequency of non-CpG-creating mutations was 0.011, whereas the average frequency of CpG-creating mutations was 0.006. The ratio between the two means was 1.9. The difference in frequencies was significant (Wilcoxon test, *p* value $< 0.01$).

### Relation between cost and genomic CpG under-representation

The relationships between costs of CpG creating mutations and the degrees of CG dinucleotide under/over-representation (Rho statistic values) were assessed for all viral genes/genomes used in our study. The Rho statistic is obtained by dividing the frequency of dinucleotide xy by the product of frequencies of nucleotide x and nucleotide y, and calculated using the ’seqinr’ package (Charif et al. 2004) in R. The results showed overall significant negative correlation (Spearman’s $\rho = -0.37$, $p =0.0005$), indicating the higher the costs of CpG creating mutations, the more CG dinucleotide was underrepresented . Correlation was also assessed separately for A G and T C mutations, which resulted in significant negative correlation for T C mutations (Spearman’s $\rho = -0.43$, $P =0.004$), and marginally significant correlation for A G mutations (Spearman’s $\rho = -0.29$, $P = 0.06$).

## Results

We collected 42 viral datasets from online sources (Genbank, Los Alamos HIV Database, Virus Pathogen Resource (VPR)), each of which is a group of viral sequences of the same species, subtype and gene (see Table 1). Each sequence in a dataset came from an individual host from various parts of the world. The mean number of sequences in a dataset is 2501, median 579, with a maximum at 24,005 and a minimum at 41. The mean number of nucleotides for each sequence is 3710, median 1706, with a maximum of 14,469 and a minimum of 294. We established a minimum cut off of 60,000 data points per dataset (number of nucleotides $\times$ number of sequences), viruses or genes with less data available were not included.

We use the following approach. We assume that mutations occur at random, but are then subject to selection and drift. Selection and drift can act within hosts or at the transmission stage. For most mutations, selection will act to purge the mutations from the viral population (within-host population or the global population). Whether within-host or between-host effects are more important is not clear for most viruses, but either way, we expect that more deleterious mutations are less likely to be observed often, and more benign mutations will be observed more often. The main focus of our paper is to determine whether CpG-creating mutations are observed less often in each of the 42 datasets than (otherwise similar) non-CpG-creating mutations. We focus on A$\rightarrow$G and T$\rightarrow$C mutations, because transition mutations are more common in viruses than transversion mutations and only these transition mutations can create CpG sites.

To check whether our approach was sound, in principle, and whether there was sufficient power to asses the cost of CpG-creating mutations, we first tested whether synonymous mutations were observed at higher frequencies than non-synonymous mutations using the non-parametric Wilcoxon test. All tests are one-tailed, because we expect synonymous mutations to occur at a higher frequency than non-synonymous mutations. To make our approach for non-synonymous sites as similar as possible to our approach for CpG-creating mutations, we also focus solely on A$\rightarrow$G and T$\rightarrow$C mutations. We observed a significant difference between the frequencies of synonymous mutations and non-synonymous mutations for 38 of the 42 datasets analyzed (90.5%) (Table 2).

As an additional test to make sure our approach was sound, we ran simulations in SLIM (Haller and Messer 2019). We simulated virus genomes with costly CpG-creating mutations and less costly non-CpG-creating mutations in 200 patients and find that as expected, the results show a higher average frequency for the non-CpG-creating mutations. See supplementary figure S1.

Our study focused on transition mutations that result in CpG sites. We focused on transition mutations because they occur at a much higher rate than tranversion mutations, and provide greater power to detect meaningful differences. There are two ways for a CpG site to be formed by a transition mutation; (1) a C precedes an A (CA) and the A mutates to a G, and (2) a T precedes a G (TG) and the T mutates to a C (see Fig. 2).

Both synonymous and non-synonymous mutations can create CpG sites. For example, when a TCA codon, which encodes Serine, mutates where the A becomes G (A$\rightarrow$G), making the codon to TCG, this will result in a new CpG site without changing the amino acid. Comparing synonymous CpG-creating versus synonymous non-CpG-creating mutations, we found that the frequencies of non-CpG mutations were significantly higher than those of CpG-creating mutations in 32 of the data sets (76.2%) for A$\rightarrow$G mutations and 28 of the data sets (66.7%) for T$\rightarrow$C mutations.

Non-synonymous mutations result in an amino acid change that alters the protein. Mutations which create a CpG site and cause a non-synonymous amino acid change are called non-synonymous CpG-creating mutations. While mutations that are non-synonymous but do not create CpG sites are called non-synonymous non-CpG-creating mutations. When comparing non-synonymous CpG-creating versus non-synonymous non-CpG-creating mutations, non-CpG-creating mutations had a significantly higher frequency than CpG-creating mutations 23.8% of the time for A$\rightarrow$G mutations and 40.5% for T$\rightarrow$C mutations (See Table 2).

From our collection of viruses, we show results from three datasets as examples (Fig. 3). Only A$\rightarrow$G and T$\rightarrow$C mutations can form CpG sites, but here we also show C$\rightarrow$T and G$\rightarrow$A nucleotides as a comparison. Our results varied, they ranged from exhibiting high mutation frequencies to low mutation frequencies and significant to not significant test results. The three examples chosen show the diversity of our results.

In each graph, four categories of mutations are compared with one another: synonymous non-CpG-creating mutations (green), synonymous CpG-creating mutations (blue), non-synonymous non-CpG-creating mutations (orange), and non-synonymous CpG-creating mutations (red). Each colored point is the mutation frequency observed at a single position within each of these categories, along with the mean value and standard error bars (one standard error above and below the mean) in black.

Figure 3a shows mutation frequencies for Dengue 1. Dengue’s genome is comprised of one large polyprotein. For Dengue 1, we have 1783 sequences and 10,176 nucleotides, making this a particularly large dataset. We show frequencies of all 10,176 sites in the genome, split into the four different transition mutations (A$\rightarrow$G, T$\rightarrow$C, C$\rightarrow$T, G$\rightarrow$A) and then split into synonymous(green and blue) and non-synonymous (orange and red). Non-CpG-creating mutations are green and orange, while CpG-creating mutations are red and blue. For this data set, all tested comparisons are significantly different (*p* < 0.01, Wilcoxon test). Synonymous CpG-creating mutations occur at lower frequencies than synonymous non-CpG-creating mutations, for both A$\rightarrow$G and T$\rightarrow$C mutations (green vs blue and orange vs red respectively). There is also a significant difference between the synonymous and non-synonymous mutations for both A$\rightarrow$G and T$\rightarrow$C mutations.

Next, we show mutation frequencies for the HA gene (hemagglutinin) of the Influenza A H3N2 strain (Fig. 3c, d). The *p* values show that non-CpG-creating mutations occur at higher frequencies than CpG-creating mutations for synonymous A$\rightarrow$G and T$\rightarrow$C mutations. For the synonymous T$\rightarrow$C mutations, the graph shows that the mean frequencies are almost the same, but the non-parametric Wilcoxon test still detects a significant difference (*p* < 0.01) (Fig. 3d). For non-synonymous mutations, we find a significant difference between CpG-creating and non-CpG-creating mutations for T$\rightarrow$C but not A$\rightarrow$G mutations. The difference in frequencies between synonymous and non-synonymous mutations is significant for both A$\rightarrow$G and T$\rightarrow$C mutations.

Next, we show the results for Human Respiratory Syncytial Virus G gene (Fig. 3e, f). The results here are very similar to the Influenza virus in the figure: all tests are significant except for the difference between CpG-creating and non-CpG-creating mutations for non-synonymous A$\rightarrow$G mutations (Fig. 3f).

### Cost of CpG-creating mutations across all datasets

With a Wilcoxon test, we could determine whether CpG-creating mutations occur at lower frequencies than otherwise similar non-CpG-creating mutations, but it does not give us a sense of the effect size of this effect. To get a better sense of how much less frequent CpG-creating mutations are (and thus roughly how much more costly) we divided the mean frequency of non-CpG-creating mutations by the mean frequency of CpG-creating mutations for each of the datasets (Fig. 4). We graphed only the synonymous mutations as they more often showed a significant CpG effect.

We calculated two ratios for each dataset: (1) the ratio of the mean frequency of synonymous, A$\rightarrow$G, non-CpG-creating mutations and synonymous, A$\rightarrow$G, CpG-creating mutations (red), and (2) the ratio of the mean frequency of synonymous, T$\rightarrow$C, non-CpG-creating mutations and synonymous, T$\rightarrow$C, CpG-creating mutations (blue). When these ratios are above 1 it means that the non-CpG-creating mutations have a higher average frequency than CpG-creating mutations, which shows that the CpG-creating mutations are more costly. The higher the frequency, the higher the cost of CpG-creating mutations relative to the cost of non-CpG-creating mutations. The black line in the Fig. 4 indicates the *ratio* = 1. Most, though not all, viruses analyzed show ratios higher than 1 (above the solid black line).

We performed a sign-test (exact binomial test) to determine whether we were significantly more likely to find cost ratios higher than 1 versus cost ratios lower than 1. We found a highly significant result for both types of mutations, which confirms that the over-representation of positive cost ratios in Fig. 4 is not due to chance. For A$\rightarrow$G mutations (39 ratios higher than 1 out of 42 observations), *p* value = 5.63e$-$09, and for T$\rightarrow$C mutations (37 ratios higher than 1 out of 42 observations) *p* value = 4.43e$-$07.

In Fig. 4 the viruses are arranged by genus, with RNA viruses on the left and DNA viruses on the right. We see that the calculated frequency ratios are consistently above 1 for Dengue 1–4, Hepatitis C, HIV, Influenza A, Human Respiratory Syncytial virus, Measles, Rhino viruses, Rota A virus, BK polyoma, Human Boca and Parvo virus. Results are mixed (though still majority above 1 for Parainfluenza, Influenza B, Entero viruses Hepatitis B, Herpes virus and Human papiloma.

There is a pattern among groups of viruses where one type of mutation is more costly than the other. In Dengue and Human Parainfluenza CpG-creating T$\rightarrow$ C mutations are relatively more costly than CpG-creating A$\rightarrow$G mutations. In Entero and Hepatitis B, on the other hand CpG-creating A$\rightarrow$G mutations are more costly than CpG-creating T$\rightarrow$C mutations. It is unclear whether this is an artifact of our dataset or a real effect.

Since we suspect that the amount of data available per dataset may affect our results, we plotted the product of the number of sequences and the number of nucleotides per dataset at the bottom of Fig. 4. In a separate figure (Fig. 5 and supplementary figure S3), we show how the amount of available data affects whether we find significant results for A$\rightarrow$G or T$\rightarrow$C mutations or both. In these figures, each dot represents a dataset, the *x* axis shows the number of sequences in each dataset and *y* axis shows the number of sites at which a transition mutation creates a CpG site. Blue triangles indicated two significant Wilcoxon tests (for A$\rightarrow$G and T$\rightarrow$C mutations), green squares indicate one significant result and red dots indicate no significant result. The figure shows that, in general, having more data makes it more likely to find one or two significant results. Figure 5a shows the comparison synonymous CpG-creating versus synonymous non-CpG-creating mutations. In this figure, the red and green data points are clearly clustered in the lower left corner, which suggests that the absence of significant results here is due to a lack or data. Figure 5b shows the comparison non-synonymous CpG-creating versus non-synonymous non-CpG-creating mutations. In this case, it seems that only our largest datasets lead to significant result. Finally, Fig. 5c shows the comparison synonymous versus non-synonymous mutations.

We wanted to determine whether the datasets for which we estimated high costs of CpG-creating mutations also showed a lack of CpG-sites in their genomes. To test this, we determined the relationship between the cost ratio we estimated and the CpG under-representation (Rho statistic values) and we found that overall, this relationship is indeed negative (Spearman’s $\rho ~=~-0.37$, *p* = 0.0005) (see Methods and supplementary figure S4). This could mean that the different costs we estimate in different viruses have existed for long enough evolutionary time scales to affect the genome content of the viruses we study.

## Discussion

### CpG-creating mutations are costly in most viruses

There is previous evidence that CpG-creating mutations are costly for viruses such as HIV and Polio (Theys et al. 2018; Stern et al. 2017). It is expected that such mutations are also costly in other viruses, because CpG sites are rare in many viruses (Karlin and Cardon 1994). Here we used global data for 42 viral datasets to test whether CpG sites are costly for most human viruses. For many viruses, information on within-host diversity is not readily available, so we focused on between-host diversity, using datasets with one viral sequence per patient. We expect that mutation frequencies in such datasets are determined by mutation rates, selection coefficients and stochastic effects such as drift and selective sweeps (Hartl and Clark 2007). Our main assumption here is that stochastic effects and mutation rates affect CpG-creating and non-CpG-creating mutations equally (see section on study limitations). This means that any significant difference in mutation frequencies between CpG-creating and non-CpG-creating mutations will be due to differences in selection coefficients, which allows us to determine whether CpG-creating mutations are generally more costly than non-CpG-creating mutations (Theys et al. 2018).

We found that indeed, in the majority of viruses we tested, the mutation frequencies were significantly different between CpG-creating and non-CpG-creating mutations, which shows that there is a fitness cost to CpG-creating mutations in most viruses. We found a significant effect of CpG-creating mutations in 76.2 % of datasets for synonymous A$\rightarrow$G mutations and in 66.7% of synonymous T$\rightarrow$C mutations.

To test the statistical power of our novel approach, we also tested whether we could detect a difference in frequencies between synonymous and non-synonymous mutations. We used the same datasets and methods to demonstrate that synonymous mutations occur at higher frequencies than non-synonymous mutations. We detected a significant difference between non-synonymous mutations and synonymous mutations in 90.5 % of datasets for A$\rightarrow$G mutations and also 90.5% of datasets for T$\rightarrow$C mutations. While we detect the CpG effect not as often as the effect of non-synonymous mutations, we still detect the effect in more that two-thirds of the viruses. The cost of CpG-creating mutations should probably be considered near ubiquitous in human viruses.

We also tested for an effect of CpG-creating mutations among non-synonymous mutations, but found that this effect was only detected in 23.8% of datasets for A$\rightarrow$G mutations and 40.5% of datasets for T$\rightarrow$C mutations. One reason for this low number of significant results is probably that many non-synonymous mutations occur at very low frequencies (see figure 2A, 2C, 2E).

### Quantifying the cost

After we found that a majority of viruses displayed a lower frequency of CpG-creating mutations when compared to non-CpG-creating mutations we moved on to quantify this cost. We did this separately for A$\rightarrow$G and T$\rightarrow$C mutations. For each of these two types of mutations, we calculated the ratio between the mean frequency of synonymous CpG-creating mutations and the mean frequency of synonymous non-CpG-creating mutations. We hypothesize that when CpG-mutations come with a large cost, they will be found at much lower frequencies, whereas if they come with a small cost, their frequencies will only be slightly lower than those of non-CpG-creating mutations. Therefore, the ratio we calculate will give us a sense of the relative cost of CpG sites in different viruses.

The levels of the cost ratio vary widely between viruses, with some clear differences between viral genera. For example, for HIV, the ratio is near 5 for both A$\rightarrow$G and C$\rightarrow$T mutations. This shows that CpG sites in HIV come with a large cost, as shown before based on a different data set (Theys et al. 2018). On the other hand, in Hepatitis C the ratio is close to 1 for both genotype 1A and 1B. The Wilcoxon tests were significant for Hepatitis C, but the fact that the ratio is close to 1 shows that the effect size is small. We find similar results when we look at within-host diversity for HCV using another dataset (Tisthammer, unpublished). The cost ratio for BK Polyoma is very high: we see a 100-fold difference in mean frequencies. This result is so extreme, that we are tempted to think it is not robust, but we did find that the number of CpG sites in the BK Polyoma genome is extremely low (less than 5% of the expected number, in supplementary figure S4 the two upper left dots are BK Polyoma.). This could mean that for some unknown reason CpG-sites are much more costly in BK Polyoma virus than the other viruses. Future studies could look into this.

We find more variable cost ratios in the DNA viruses than in the RNA viruses. This may be because of the smaller sample sizes for DNA viruses, or it may be that different selection pressures are at play in DNA viruses versus RNA viruses. In RNA viruses, we expect that the mammalian immune system recognizes CpG sites and forces the viruses to mimic the low CpG content in mammalian genomes (Takata et al. 2017). In DNA viruses, it is not clear if the same mechanism is at work, though unmethylated CpG sites are expected to stimulate the immune response (Hoelzer et al. 2008).

The cost ratio was calculated for both A$\rightarrow$G and C$\rightarrow$T mutations. These two ratios are not necessarily equal. In some viruses, we see surprising patterns in the cost ratios. For example, in the Dengue viruses, T$\rightarrow$C CpG-creating mutations (blue) are relatively more costly than A$\rightarrow$G mutations (red). In Influenza A however, the trend is in the other direction, where T$\rightarrow$C CpG-creating mutations (blue) are relatively less costly than A$\rightarrow$G mutations (red). Further studies are needed to determine what causes these patterns.

### Limitations and future studies

Our study has a number of limitations. We only included datasets with at least 60,000 data points per dataset (number of nucleotides $\times$ number of sequences). However, we still find that our larger datasets are more likely to yield significant results (Fig. 5). This suggests that increasing either the number of sequences or the sequence length for some of the viral datasets will increase the number of datasets with significant results.

Another limitation of our study is that we used one sequence per patient. This means that we don’t have any information on within-host diversity, and rare variants that exist within hosts will be missed. While we believe that having within-host diversity data would be useful, this study shows that even with one sequence per patient, we are able to detect costs of mutations. However, it is unclear whether this cost occurs during replication in the host, during transmission or both.

We and others have studied within-host diversity in HIV and HCV and other viruses to study costs of mutations within the host (Wang et al. 2010; Rambaut et al. 2004; Alizon et al. 2011; Theys et al. 2018). This is possible for these viruses because patients are infected for a long time and there is an expectation that mutation and selection occur within the host. For most other viruses, however, it is not clear whether it is possible to study within-host fitness costs separately from between-host effects. For example, if patients are infected with a diverse sample of the virus, then within-host mutation and selection may not be the dominant effects that shape within-host genetic diversity (Varble et al. 2014; Poon et al. 2016). For those types of viruses, studying within-host and between-host diversity may lead to the same results, and having data on within-host diversity may not necessarily increase our knowledge of fitness costs of mutations.

Finally, one of the main assumptions of this study is that the mutation rate doesn’t depend on the neighboring nucleotide. For example, we assume that an A$\rightarrow$G mutation is equally likely to occur when it is next to a *C* (creating a CpG site), or another nucleotide (not creating a CpG site). Similarly, we assume that an T$\rightarrow$C mutation is equally likely to occur when it is followed by a *G* (creating a CpG site), or another nucleotide (not creating a CpG site). In principle, it is possible that the cost we infer is due to a lower mutation rate of CpG-creating mutations. We believe that this is unlikely for several reasons. (1) Our results for A$\rightarrow$G and T$\rightarrow$C mutations are very similar, if this was due to a mutation rate effect, it would have to have the same effect on both of these mutation types. (2) Our results are consistent with results from epidemiological studies on polio (Stern et al. 2017) and in vitro studies on HIV (Takata et al. 2017; Ficarelli et al. 2019). Future studies will hopefully measure viral mutation rates with more precision.

In conclusion, we find that CpG-creating mutations are costly for most human viruses. For viruses in which we do not detect an effect of CpG-creating mutations, it is likely because of a small sample size. It was already known for some viruses that CpG-creating mutations were costly, but we have now shown that this cost occurs in most human viruses. Future work should focus on better understanding why the cost of CpG-creating mutations is higher in some viruses than others, and whether there is a relation with how the virus interacts with the human host, and possibly other hosts. We are also excited about future studies that could find what other types of mutations are costly, and we hypothesize that with the advent of artificial intelligence in population genetics (Sheehan and Song 2016; Schrider and Kern 2018), we will be able to get a much more complete understanding of the fitness landscape of viruses. Another interesting future direction would be to use modeling studies to determine the effects of the cost of these CpG-creating mutations on the effective population size and adaptive potential of viral populations.

## Electronic supplementary material

Below is the link to the electronic supplementary material.

Supplementary material 1 (pdf 32615 KB)

Supplementary material 2 (pdf 634 KB)
