# Supplementary material for: CpG-creating mutations are costly in many human viruses
Source: Evol Ecol. 2020 Apr 24;34(3):339–59. doi: 10.1007/s10682-020-10039-z (PMC7245597; doi:10.1007/s10682-020-10039-z)
Supplement: Supplementary file 1 — Supplementary material 1 (pdf 32615 KB) [file 10682_2020_10039_MOESM1_ESM.pdf]

Dengue 1

A

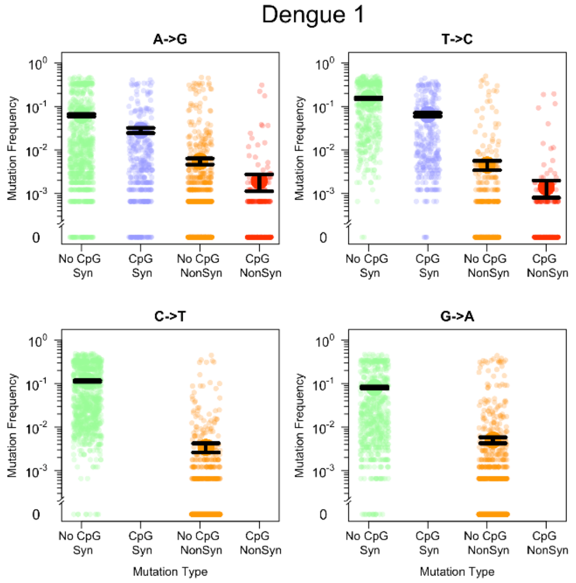

B

Dengue 1

Mutation Type	Comparison	P–Value
A–>G	Syn: CpG v NonCpG	< 0.01
	NonSyn: CpG v NonCpG	< 0.01
	Syn v NonSyn	< 0.01
T–>C	Syn: CpG v NonCpG	< 0.01
	NonSyn: CpG v NonCpG	< 0.01
	Syn v NonSyn	< 0.01

Supplementary Fig 1. (a) Transition mutation frequencies for the analysis of Dengue 1 with mean and standard error (black line). (b) Wilcoxon test results, where the shade of the blue in the P-value cell represents the significance level; darker the shade, the more significant the results.

Dengue 2

A

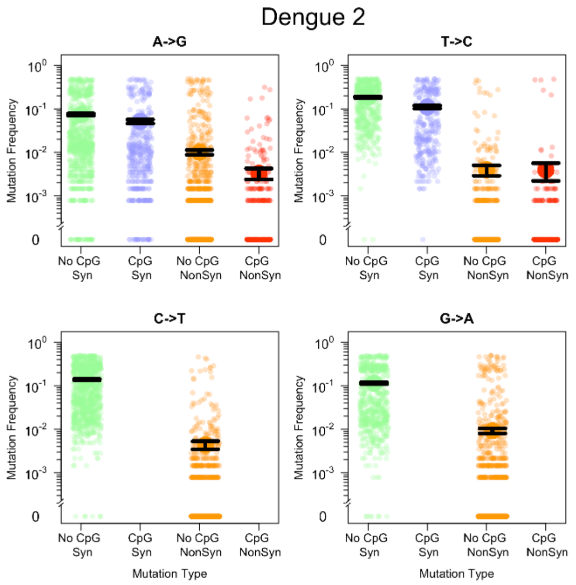

B

Dengue 2

Mutation Type	Comparison	P–Value
A–>G	Syn: CpG v NonCpG	< 0.01
	NonSyn: CpG v NonCpG	0.0128
	Syn v NonSyn	< 0.01
T–>C	Syn: CpG v NonCpG	< 0.01
	NonSyn: CpG v NonCpG	0.0297
	Syn v NonSyn	< 0.01

Supplementary Fig 2. (a) Transition mutation frequencies for the analysis of Dengue 2 with mean and standard error (black line). (b) Wilcoxon test results, where the shade of the blue in the P-value cell represents the significance level; darker the shade, the more significant the results.

Dengue 3

A

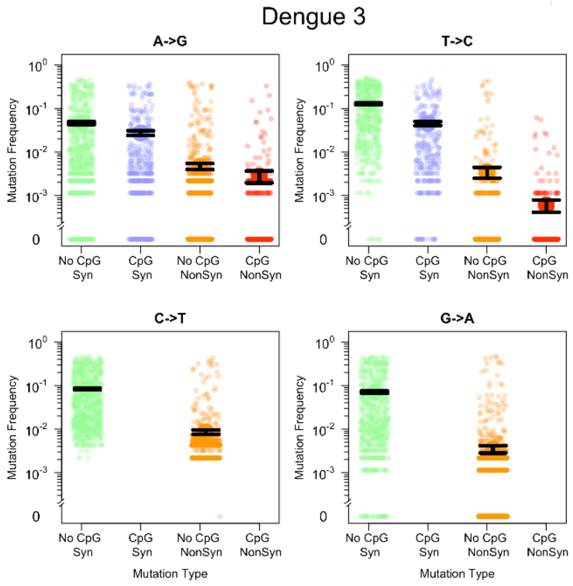

B

Dengue 3

Mutation Type	Comparison	P–Value
A–>G	Syn: CpG v NonCpG	< 0.01
	NonSyn: CpG v NonCpG	0.0191
	Syn v NonSyn	< 0.01
T–>C	Syn: CpG v NonCpG	< 0.01
	NonSyn: CpG v NonCpG	< 0.01
	Syn v NonSyn	< 0.01

Supplementary Fig 3. (a) Transition mutation frequencies for the analysis of Dengue 3 with mean and standard error (black line). (b) Wilcoxon test results, where the shade of the blue in the P-value cell represents the significance level; darker the shade, the more significant the results.

Dengue 4

A

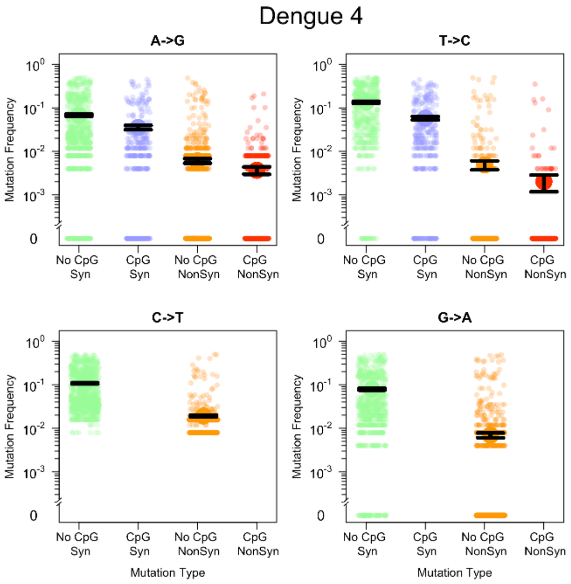

B

Dengue 4

Mutation Type	Comparison	P−Value
A−>G	Syn: CpG v NonCpG	< 0.01
	NonSyn: CpG v NonCpG	0.0724
	Syn v NonSyn	< 0.01
T−>C	Syn: CpG v NonCpG	< 0.01
	NonSyn: CpG v NonCpG	< 0.01
	Syn v NonSyn	< 0.01

Supplementary Fig 4. (a) Transition mutation frequencies for the analysis of Dengue 4 with mean and standard error (black line). (b) Wilcoxon test results, where the shade of the blue in the P-value cell represents the significance level; darker the shade, the more significant the results.

HCV1 A

A

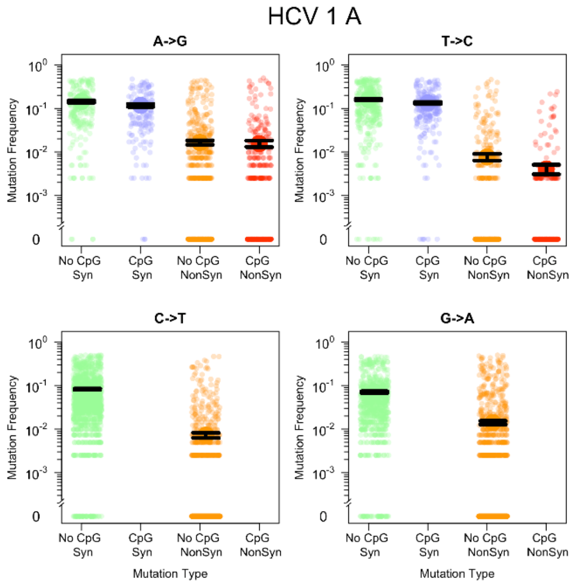

B

HCV 1 A

Mutation Type	Comparison	P–Value
A–>G	Syn: CpG v NonCpG	0.021
	NonSyn: CpG v NonCpG	0.385
	Syn v NonSyn	< 0.01
T–>C	Syn: CpG v NonCpG	0.027
	NonSyn: CpG v NonCpG	0.193
	Syn v NonSyn	< 0.01

Supplementary Fig 5. (a) Transition mutation frequencies for the analysis of HCV 1 A with mean and standard error (black line). (b) Wilcoxon test results, where the shade of the blue in the P-value cell represents the significance level; darker the shade, the more significant the results.

HCV 1 B

A

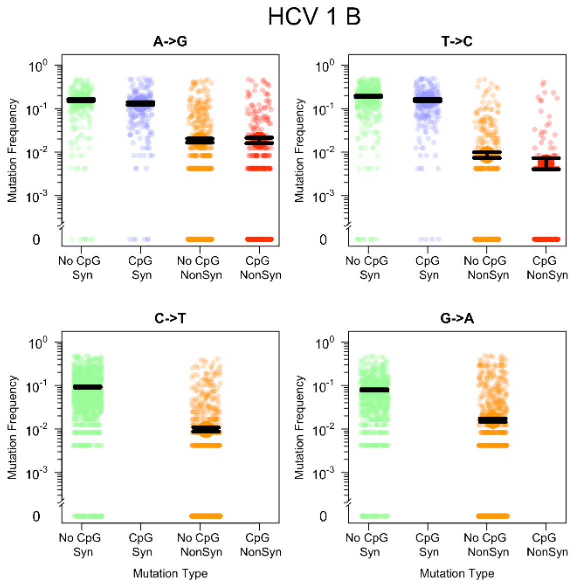

B

HCV 1 B

Mutation Type	Comparison	P–Value
A–>G	Syn: CpG v NonCpG	< 0.01
	NonSyn: CpG v NonCpG	0.891
	Syn v NonSyn	< 0.01
T–>C	Syn: CpG v NonCpG	< 0.01
	NonSyn: CpG v NonCpG	< 0.01
	Syn v NonSyn	< 0.01

Supplementary Fig 6. (a) Transition mutation frequencies for the analysis of HCV 1 B with mean and standard error (black line). (b) Wilcoxon test results, where the shade of the blue in the P-value cell represents the significance level; darker the shade, the more significant the results.

HIV pol Gene

A

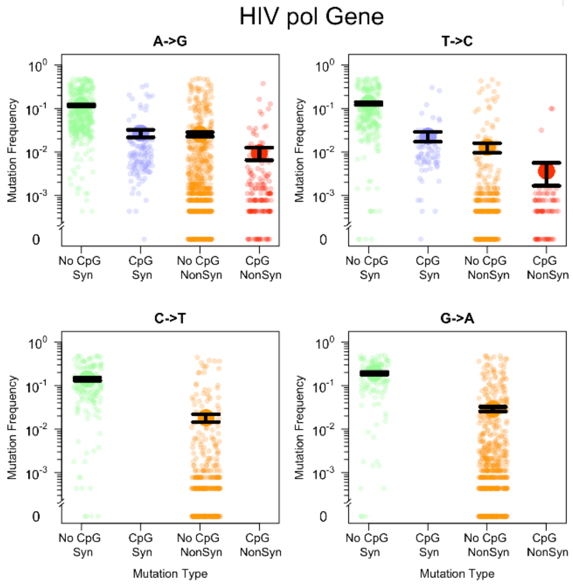

B

HIV pol Gene

Mutation Type	Comparison	P–Value
A–>G	Syn: CpG v NonCpG	< 0.01
	NonSyn: CpG v NonCpG	< 0.01
	Syn v NonSyn	< 0.01
T–>C	Syn: CpG v NonCpG	< 0.01
	NonSyn: CpG v NonCpG	0.156
	Syn v NonSyn	< 0.01

Supplementary Fig 7. (a) Transition mutation frequencies for the analysis of HIV Pol with mean and standard error (black line). (b) Wilcoxon test results, where the shade of the blue in the P-value cell represents the significance level; darker the shade, the more significant the results.

Human Parainfluenza 1 HN

A

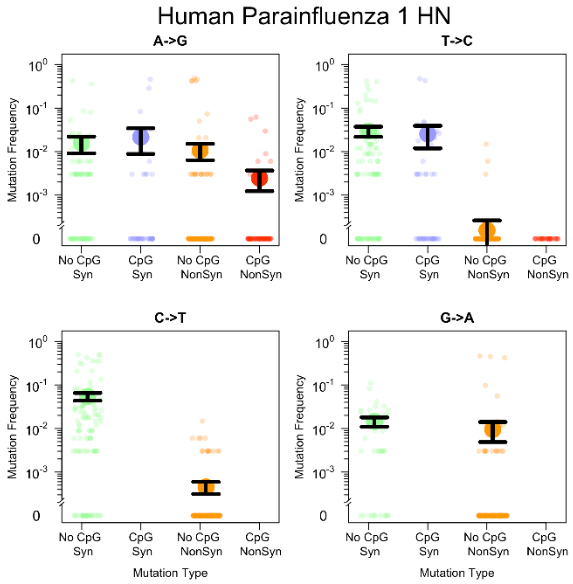

B

Human Parainfluenza 1 HN

Mutation Type	Comparison	P−Value
A−>G	Syn: CpG v NonCpG	< 0.01
	NonSyn: CpG v NonCpG	0.68
	Syn v NonSyn	< 0.01
T−>C	Syn: CpG v NonCpG	0.0132
	NonSyn: CpG v NonCpG	0.214
	Syn v NonSyn	< 0.01

Supplementary Fig 8. (a) Transition mutation frequencies for the analysis of Human Parainfluenza 1 HN with mean and standard error (black line). (b) Wilcoxon test results, where the shade of the blue in the P-value cell represents the significance level; darker the shade, the more significant the results.

Human Parainfluenza 3 HN

A

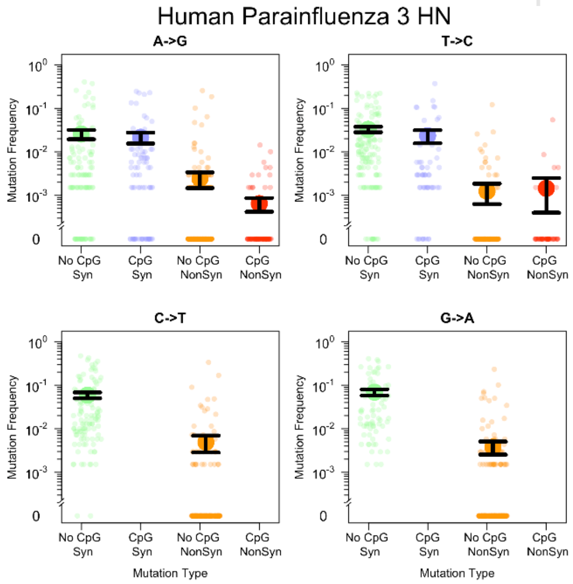

B

Human Parainfluenza 3 HN

Mutation Type	Comparison	P−Value
A−>G	Syn: CpG v NonCpG	0.271
	NonSyn: CpG v NonCpG	0.45
	Syn v NonSyn	< 0.01
T−>C	Syn: CpG v NonCpG	< 0.01
	NonSyn: CpG v NonCpG	0.782
	Syn v NonSyn	< 0.01

Supplementary Fig 9. (a) Transition mutation frequencies for the analysis of Human Parainfluenza 3 HN with mean and standard error (black line). (b) Wilcoxon test results, where the shade of the blue in the P-value cell represents the significance level; darker the shade, the more significant the results.

Human Parainfluenza 1

A

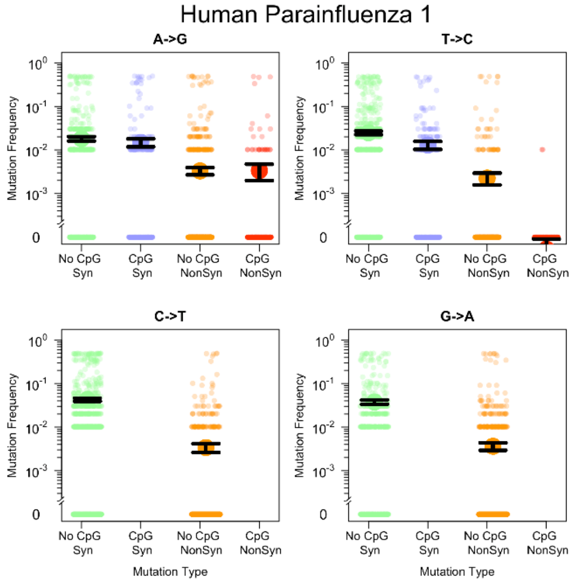

B

Human Parainfluenza 1

Mutation Type	Comparison	P−Value
A−>G	Syn: CpG v NonCpG	< 0.01
	NonSyn: CpG v NonCpG	0.378
	Syn v NonSyn	< 0.01
T−>C	Syn: CpG v NonCpG	< 0.01
	NonSyn: CpG v NonCpG	< 0.01
	Syn v NonSyn	< 0.01

Supplementary Fig 10. (a) Transition mutation frequencies for the analysis of Human Parainfluenza 1 with mean and standard error (black line). (b) Wilcoxon test results, where the shade of the blue in the P-value cell represents the significance level; darker the shade, the more significant the results.

Human Parainfluenza 3

A

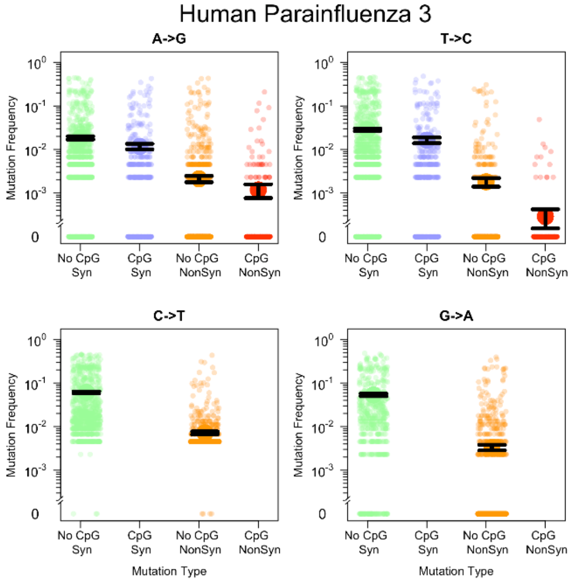

B

Human Parainfluenza 3

Mutation Type	Comparison	P–Value
A–>G	Syn: CpG v NonCpG	< 0.01
	NonSyn: CpG v NonCpG	0.0151
	Syn v NonSyn	< 0.01
T–>C	Syn: CpG v NonCpG	< 0.01
	NonSyn: CpG v NonCpG	< 0.01
	Syn v NonSyn	< 0.01

Supplementary Fig 11. (a) Transition mutation frequencies for the analysis of Human Parainfluenza 3 with mean and standard error (black line). (b) Wilcoxon test results, where the shade of the blue in the P-value cell represents the significance level; darker the shade, the more significant the results.

Influenza A HA H1N1

A

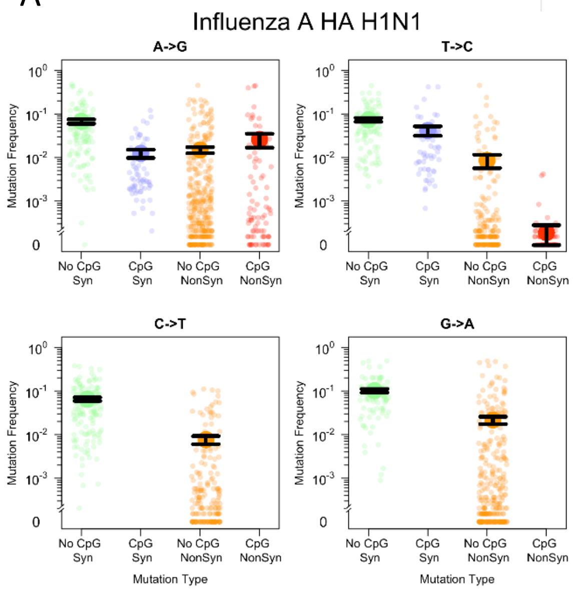

B

Influenza A HA H1N1

Mutation Type	Comparison	P–Value
A–>G	Syn: CpG v NonCpG	< 0.01
	NonSyn: CpG v NonCpG	0.328
	Syn v NonSyn	< 0.01
T–>C	Syn: CpG v NonCpG	< 0.01
	NonSyn: CpG v NonCpG	< 0.01
	Syn v NonSyn	< 0.01

Supplementary Fig 12. (a) Transition mutation frequencies for the analysis of Influenza A HA H1N1 with mean and standard error (black line). (b) Wilcoxon test results, where the shade of the blue in the P-value cell represents the significance level; darker the shade, the more significant the results.

Influenza A HA H3N2

A

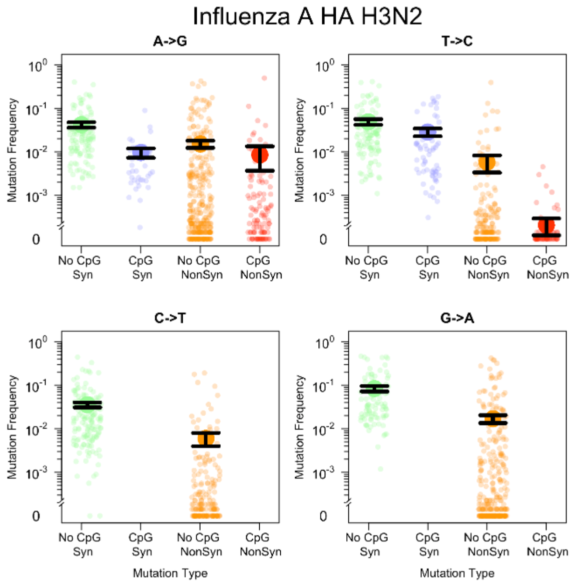

B

Influenza A HA H3N2

Mutation Type	Comparison	P–Value
A–>G	Syn: CpG v NonCpG	< 0.01
	NonSyn: CpG v NonCpG	0.0628
	Syn v NonSyn	< 0.01
T–>C	Syn: CpG v NonCpG	< 0.01
	NonSyn: CpG v NonCpG	< 0.01
	Syn v NonSyn	< 0.01

Supplementary Fig 13. (a) Transition mutation frequencies for the analysis of Influenza A HA H3N2 with mean and standard error (black line). (b) Wilcoxon test results, where the shade of the blue in the P-value cell represents the significance level; darker the shade, the more significant the results.

Influenza A NA H1N1

A

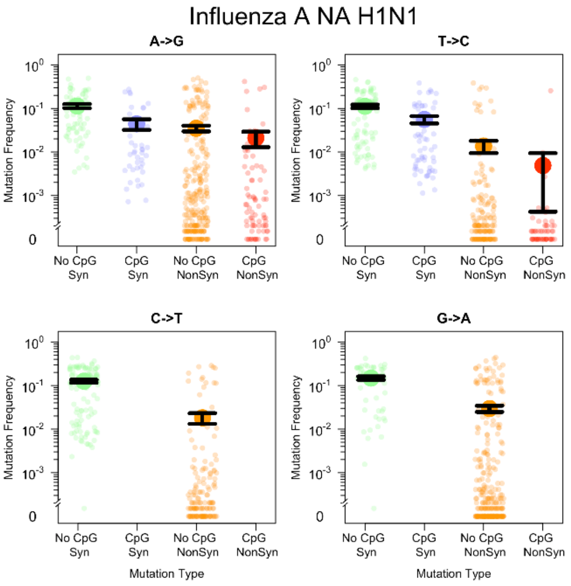

B

Influenza A NA H1N1

Mutation Type	Comparison	P–Value
A–>G	Syn: CpG v NonCpG	< 0.01
	NonSyn: CpG v NonCpG	0.19
	Syn v NonSyn	< 0.01
T–>C	Syn: CpG v NonCpG	< 0.01
	NonSyn: CpG v NonCpG	< 0.01
	Syn v NonSyn	< 0.01

Supplementary Fig 14. (a) Transition mutation frequencies for the analysis of Influenza A NA H1N1 with mean and standard error (black line). (b) Wilcoxon test results, where the shade of the blue in the P-value cell represents the significance level; darker the shade, the more significant the results.

Influenza A NA H3N2

A

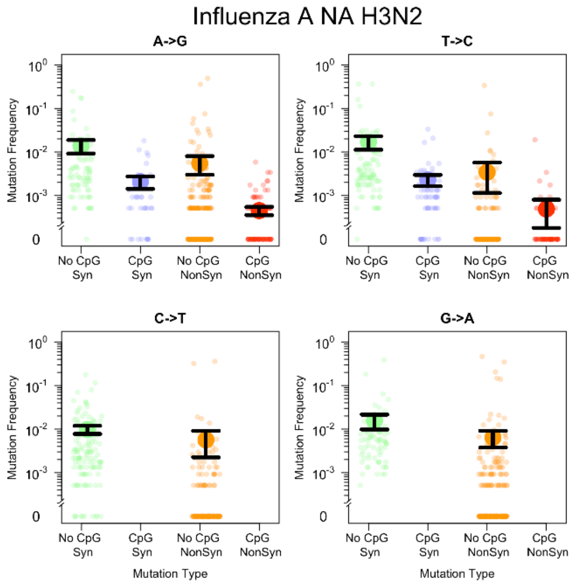

B

Influenza A NA H3N2

Mutation Type	Comparison	P–Value
A–>G	Syn: CpG v NonCpG	< 0.01
	NonSyn: CpG v NonCpG	0.224
	Syn v NonSyn	< 0.01
T–>C	Syn: CpG v NonCpG	< 0.01
	NonSyn: CpG v NonCpG	0.126
	Syn v NonSyn	< 0.01

Supplementary Fig 15. (a) Transition mutation frequencies for the analysis of Influenza A NA H3N2 with mean and standard error (black line). (b) Wilcoxon test results, where the shade of the blue in the P-value cell represents the significance level; darker the shade, the more significant the results.

Influenza B HA

A

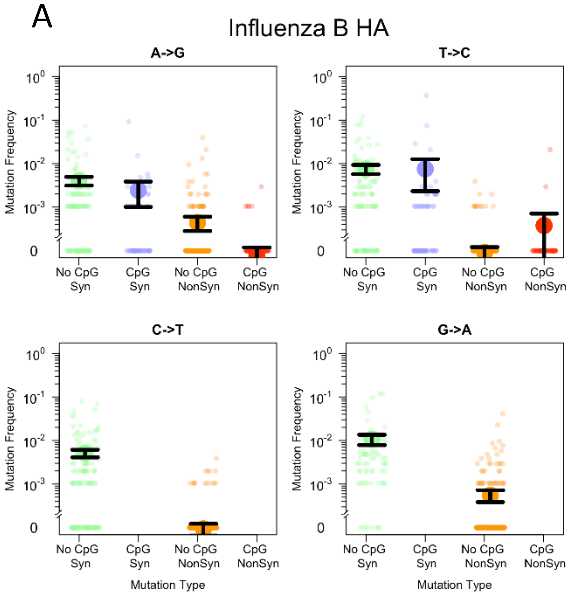

B

Influenza B HA

Mutation Type	Comparison	P−Value
A−>G	Syn: CpG v NonCpG	< 0.01
	NonSyn: CpG v NonCpG	0.0946
	Syn v NonSyn	< 0.01
T−>C	Syn: CpG v NonCpG	< 0.01
	NonSyn: CpG v NonCpG	0.162
	Syn v NonSyn	< 0.01

Supplementary Fig 16. (a) Transition mutation frequencies for the analysis of Influenza B HA with mean and standard error (black line). (b) Wilcoxon test results, where the shade of the blue in the P-value cell represents the significance level; darker the shade, the more significant the results.

Influenza B NA

A

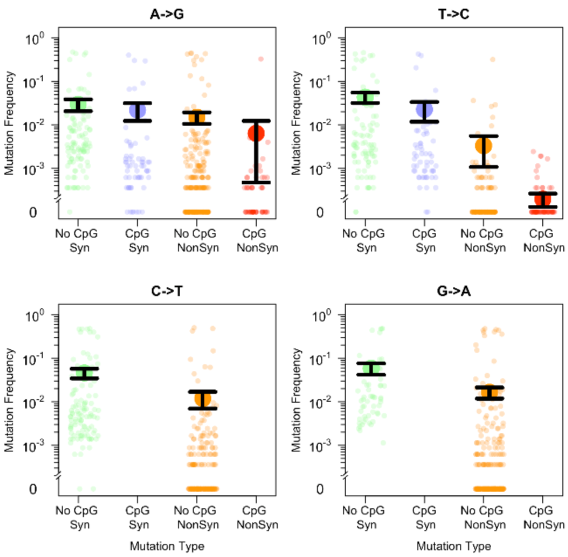

B

Influenza B NA

Mutation Type	Comparison	P–Value
A–>G	Syn: CpG v NonCpG	< 0.01
	NonSyn: CpG v NonCpG	0.0413
	Syn v NonSyn	< 0.01
T–>C	Syn: CpG v NonCpG	< 0.01
	NonSyn: CpG v NonCpG	0.119
	Syn v NonSyn	< 0.01

Supplementary Fig 17. (a) Transition mutation frequencies for the analysis of Influenza B NA with mean and standard error (black line). (b) Wilcoxon test results, where the shade of the blue in the P-value cell represents the significance level; darker the shade, the more significant the results.

Entero A Vp1 EVA71

A

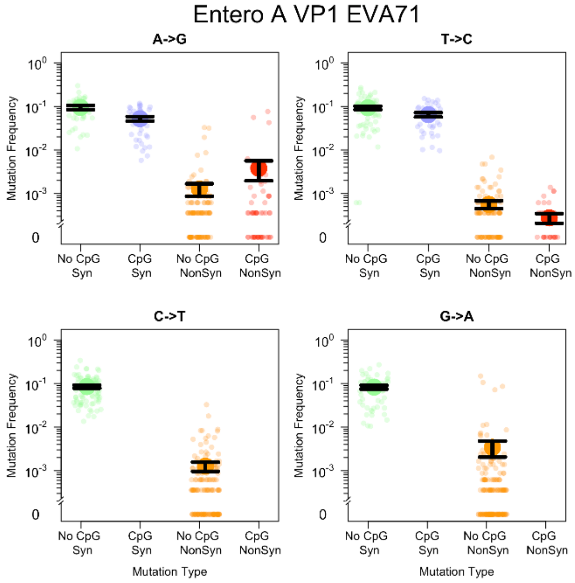

B

Entero A VP1 EVA71

Mutation Type	Comparison	P−Value
A−>G	Syn: CpG v NonCpG	< 0.01
	NonSyn: CpG v NonCpG	0.384
	Syn v NonSyn	< 0.01
T−>C	Syn: CpG v NonCpG	0.0221
	NonSyn: CpG v NonCpG	0.253
	Syn v NonSyn	< 0.01

Supplementary Fig 18. (a) Transition mutation frequencies for the analysis of Entero A VP1 Eva71 with mean and standard error (black line). (b) Wilcoxon test results, where the shade of the blue in the P-value cell represents the significance level; darker the shade, the more significant the results.

Entero A Vp2 EVA71

A

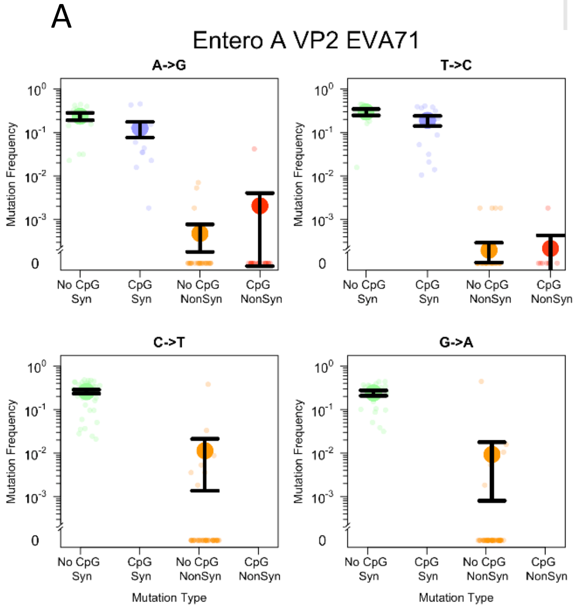

B

Entero A VP2 EVA71

Mutation Type	Comparison	P−Value
A−>G	Syn: CpG v NonCpG	0.0783
	NonSyn: CpG v NonCpG	0.477
	Syn v NonSyn	< 0.01
T−>C	Syn: CpG v NonCpG	0.0847
	NonSyn: CpG v NonCpG	0.545
	Syn v NonSyn	< 0.01

Supplementary Fig 19. (a) Transition mutation frequencies for the analysis of Entero A VP2 Eva71 with mean and standard error (black line). (b) Wilcoxon test results, where the shade of the blue in the P-value cell represents the significance level; darker the shade, the more significant the results.

Entero B Vp1 Echovirus30

A

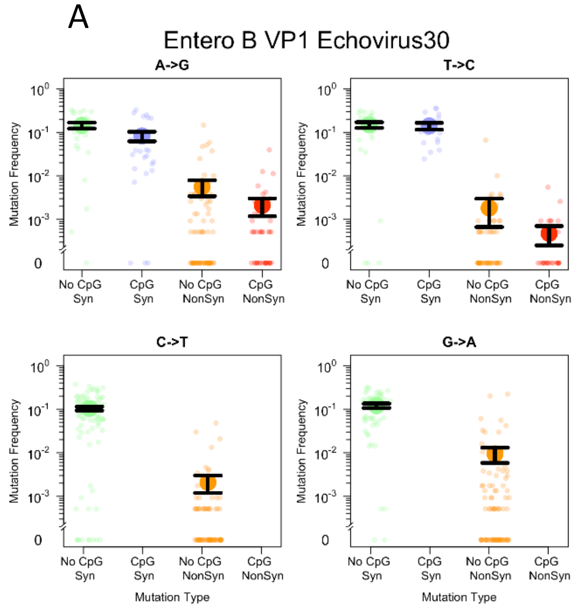

B

Entero B VP1 Echovirus30

Mutation Type	Comparison	P−Value
A−>G	Syn: CpG v NonCpG	0.0178
	NonSyn: CpG v NonCpG	0.664
	Syn v NonSyn	< 0.01
T−>C	Syn: CpG v NonCpG	0.412
	NonSyn: CpG v NonCpG	0.204
	Syn v NonSyn	< 0.01

Supplementary Fig 20. (a) Transition mutation frequencies for the analysis of Entero B VP1 Echovirus30 with mean and standard error (black line). (b) Wilcoxon test results, where the shade of the blue in the P-value cell represents the significance level; darker the shade, the more significant the results.

Entero B Vp2 Echovirus30

A

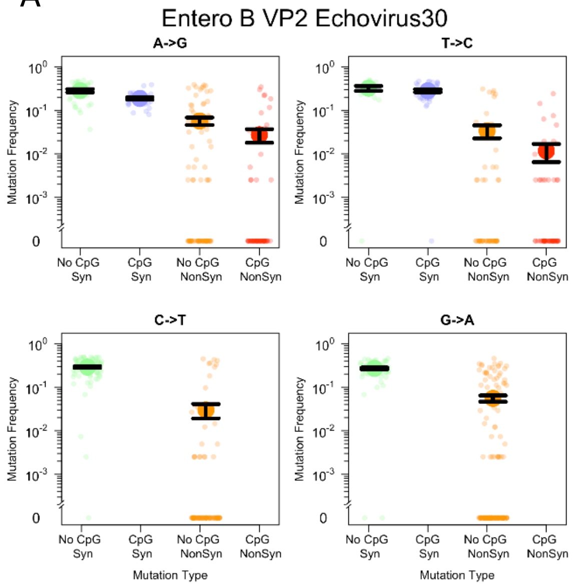

B

Entero B VP2 Echovirus30

Mutation Type	Comparison	P−Value
A−>G	Syn: CpG v NonCpG	< 0.01
	NonSyn: CpG v NonCpG	< 0.01
	Syn v NonSyn	< 0.01
T−>C	Syn: CpG v NonCpG	0.128
	NonSyn: CpG v NonCpG	0.196
	Syn v NonSyn	< 0.01

Supplementary Fig 21. (a) Transition mutation frequencies for the analysis of Entero B VP2 Echovirus30 with mean and standard error (black line). (b) Wilcoxon test results, where the shade of the blue in the P-value cell represents the significance level; darker the shade, the more significant the results.

Entero C VP1 Polio2

A

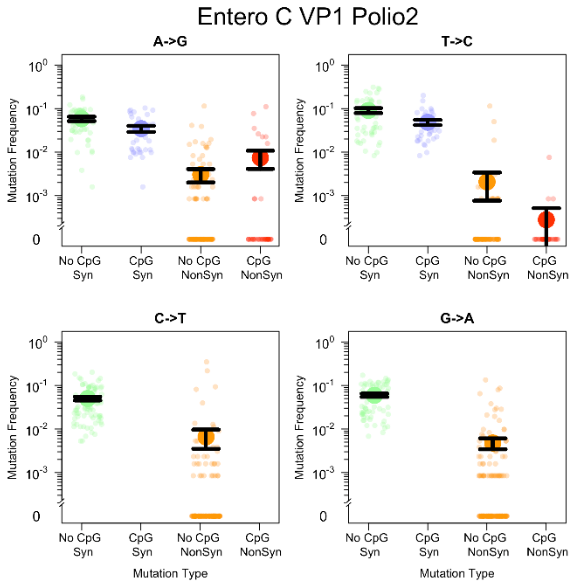

B

Entero C VP1 Polio2

Mutation Type	Comparison	P−Value
A−>G	Syn: CpG v NonCpG	< 0.01
	NonSyn: CpG v NonCpG	0.288
	Syn v NonSyn	< 0.01
T−>C	Syn: CpG v NonCpG	0.0247
	NonSyn: CpG v NonCpG	0.126
	Syn v NonSyn	< 0.01

Supplementary Fig 22. (a) Transition mutation frequencies for the analysis of Entero C VP1 Polio2 with mean and standard error (black line). (b) Wilcoxon test results, where the shade of the blue in the P-value cell represents the significance level; darker the shade, the more significant the results.

Entero C VP2 Polio2

A

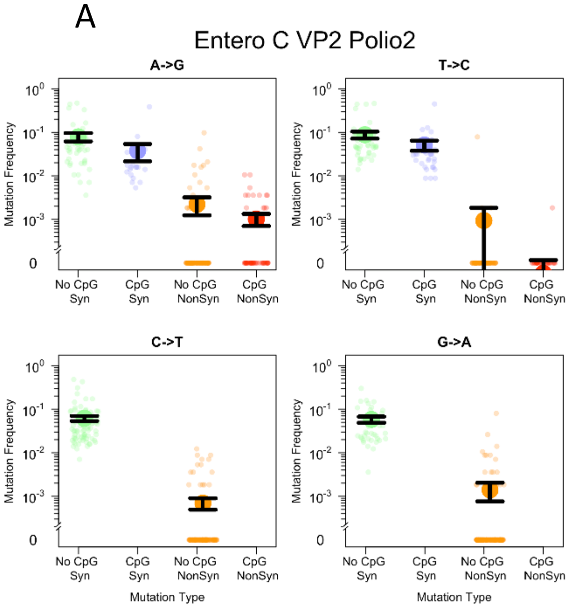

B

Entero C VP2 Polio2

Mutation Type	Comparison	P−Value
A−>G	Syn: CpG v NonCpG	< 0.01
	NonSyn: CpG v NonCpG	0.926
	Syn v NonSyn	< 0.01
T−>C	Syn: CpG v NonCpG	< 0.01
	NonSyn: CpG v NonCpG	0.488
	Syn v NonSyn	< 0.01

Supplementary Fig 23. (a) Transition mutation frequencies for the analysis of Entero C VP2 Polio2 with mean and standard error (black line). (b) Wilcoxon test results, where the shade of the blue in the P-value cell represents the significance level; darker the shade, the more significant the results.

Entero D68 VP1

A

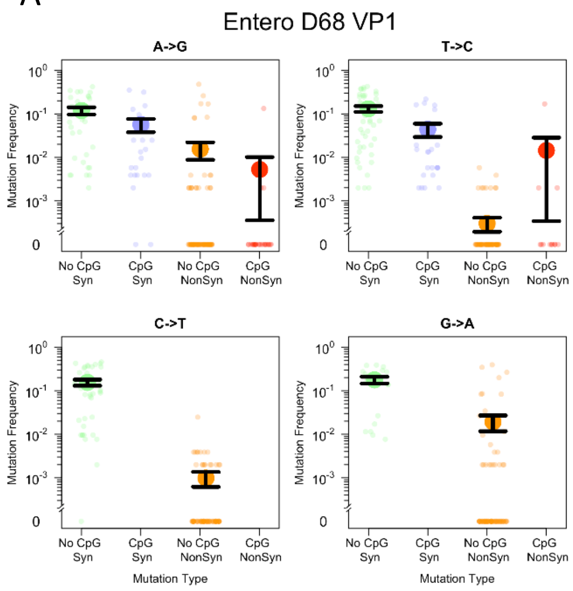

B

Entero D68 VP1

Mutation Type	Comparison	P−Value
A−>G	Syn: CpG v NonCpG	0.0158
	NonSyn: CpG v NonCpG	0.12
	Syn v NonSyn	< 0.01
T−>C	Syn: CpG v NonCpG	< 0.01
	NonSyn: CpG v NonCpG	0.897
	Syn v NonSyn	< 0.01

Supplementary Fig 24. (a) Transition mutation frequencies for the analysis of Entero D68 VP1 with mean and standard error (black line). (b) Wilcoxon test results, where the shade of the blue in the P-value cell represents the significance level; darker the shade, the more significant the results.

Human Respiratory Syncytial

A

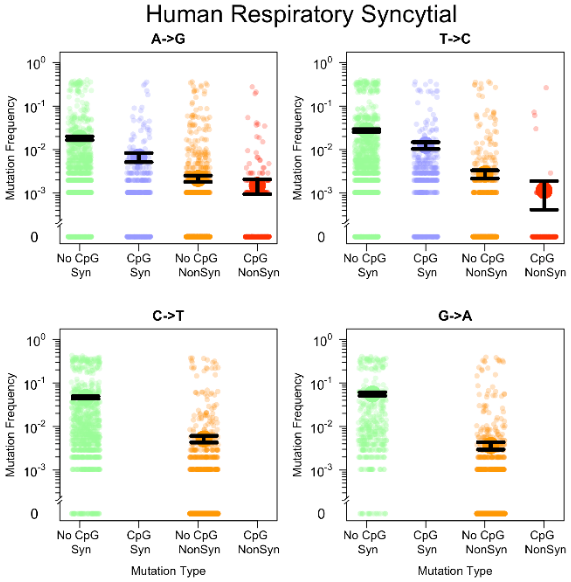

B

Human Respiratory Syncytial

Mutation Type	Comparison	P–Value
A–>G	Syn: CpG v NonCpG	< 0.01
	NonSyn: CpG v NonCpG	0.154
	Syn v NonSyn	< 0.01
T–>C	Syn: CpG v NonCpG	< 0.01
	NonSyn: CpG v NonCpG	< 0.01
	Syn v NonSyn	< 0.01

Supplementary Fig 25. (a) Transition mutation frequencies for the analysis of Human Respiratory Syncytial with mean and standard error (black line). (b) Wilcoxon test results, where the shade of the blue in the P-value cell represents the significance level; darker the shade, the more significant the results.

Measles HH

A

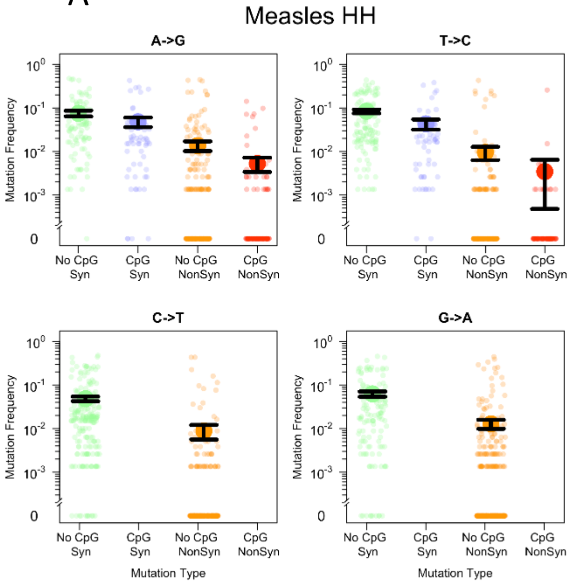

B

Measles HH

Mutation Type	Comparison	P–Value
A–>G	Syn: CpG v NonCpG	< 0.01
	NonSyn: CpG v NonCpG	0.0929
	Syn v NonSyn	< 0.01
T–>C	Syn: CpG v NonCpG	< 0.01
	NonSyn: CpG v NonCpG	0.0258
	Syn v NonSyn	< 0.01

Supplementary Fig 26. (a) Transition mutation frequencies for the analysis of Measles HH with mean and standard error (black line). (b) Wilcoxon test results, where the shade of the blue in the P-value cell represents the significance level; darker the shade, the more significant the results.

Rhino B

A

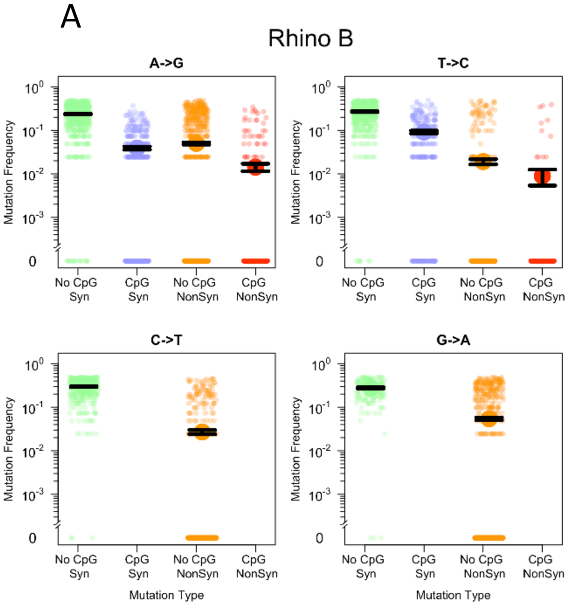

B

Rhino B

Mutation Type	Comparison	P–Value
A–>G	Syn: CpG v NonCpG	< 0.01
	NonSyn: CpG v NonCpG	< 0.01
	Syn v NonSyn	< 0.01
T–>C	Syn: CpG v NonCpG	< 0.01
	NonSyn: CpG v NonCpG	0.0426
	Syn v NonSyn	< 0.01

Supplementary Fig 27. (a) Transition mutation frequencies for the analysis of Rhino B with mean and standard error (black line). (b) Wilcoxon test results, where the shade of the blue in the P-value cell represents the significance level; darker the shade, the more significant the results.

Rhino C

A

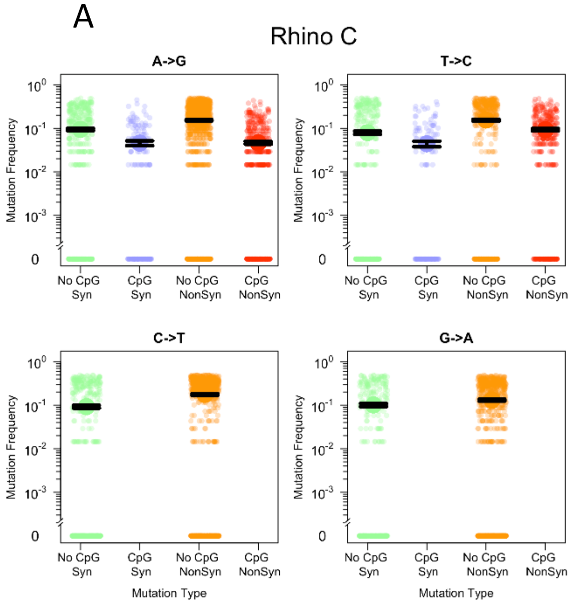

B

Rhino C

Mutation Type	Comparison	P−Value
A−>G	Syn: CpG v NonCpG	< 0.01
	NonSyn: CpG v NonCpG	< 0.01
	Syn v NonSyn	1
T−>C	Syn: CpG v NonCpG	0.38
	NonSyn: CpG v NonCpG	< 0.01
	Syn v NonSyn	1

Supplementary Fig 28. (a) Transition mutation frequencies for the analysis of Rhino C with mean and standard error (black line). (b) Wilcoxon test results, where the shade of the blue in the P-value cell represents the significance level; darker the shade, the more significant the results.

Rota A VP6

A

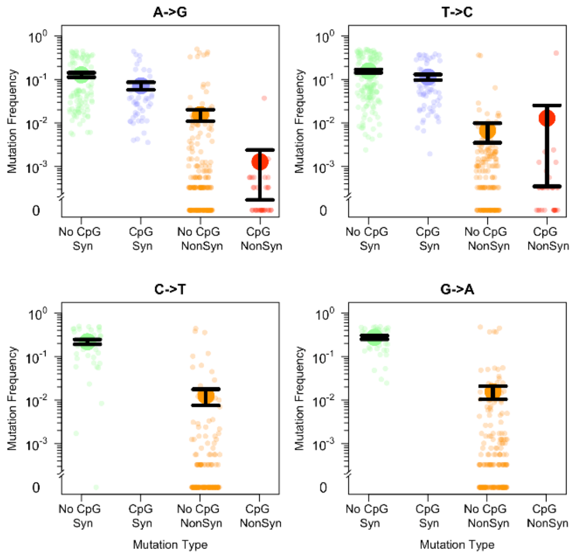

B

Rota A VP6

Mutation Type	Comparison	P−Value
A−>G	Syn: CpG v NonCpG	0.0105
	NonSyn: CpG v NonCpG	0.0228
	Syn v NonSyn	< 0.01
T−>C	Syn: CpG v NonCpG	0.0201
	NonSyn: CpG v NonCpG	0.0346
	Syn v NonSyn	< 0.01

Supplementary Fig 29. (a) Transition mutation frequencies for the analysis of Rota A VP6 with mean and standard error (black line). (b) Wilcoxon test results, where the shade of the blue in the P-value cell represents the significance level; darker the shade, the more significant the results.

BK Polyoma VP1

A

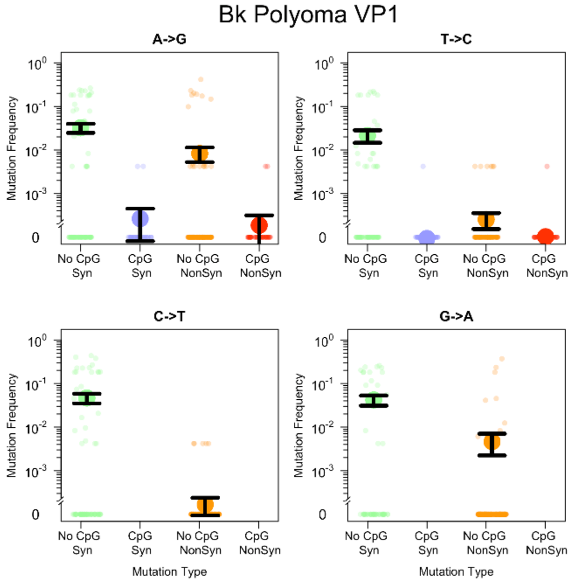

B

Bk Polyoma VP1

Mutation Type	Comparison	P−Value
A−>G	Syn: CpG v NonCpG	< 0.01
	NonSyn: CpG v NonCpG	0.158
	Syn v NonSyn	< 0.01
T−>C	Syn: CpG v NonCpG	< 0.01
	NonSyn: CpG v NonCpG	0.189
	Syn v NonSyn	< 0.01

Supplementary Fig 30. (a) Transition mutation frequencies for the analysis of BK Polyoma VP1 with mean and standard error (black line). (b) Wilcoxon test results, where the shade of the blue in the P-value cell represents the significance level; darker the shade, the more significant the results.

Human Boca VP1

A

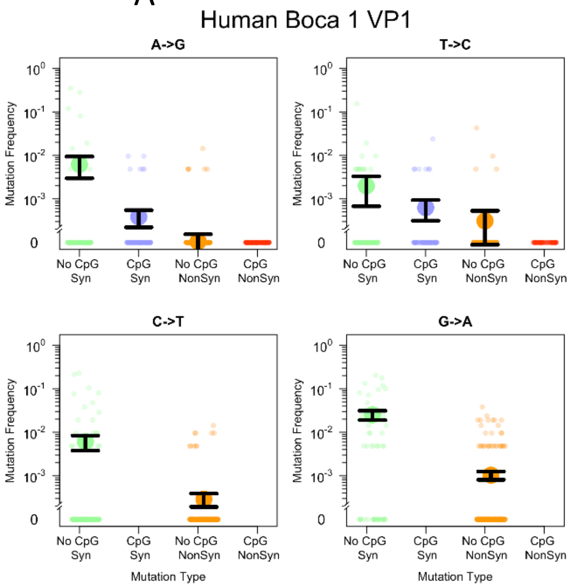

B

Human Boca 1 VP1

Mutation Type	Comparison	P−Value
A−>G	Syn: CpG v NonCpG	0.195
	NonSyn: CpG v NonCpG	0.0873
	Syn v NonSyn	< 0.01
T−>C	Syn: CpG v NonCpG	0.258
	NonSyn: CpG v NonCpG	0.141
	Syn v NonSyn	< 0.01

Supplementary Fig 31. (a) Transition mutation frequencies for the analysis of Human Boca VP1 with mean and standard error (black line). (b) Wilcoxon test results, where the shade of the blue in the P-value cell represents the significance level; darker the shade, the more significant the results.

HBV A polymerase

A

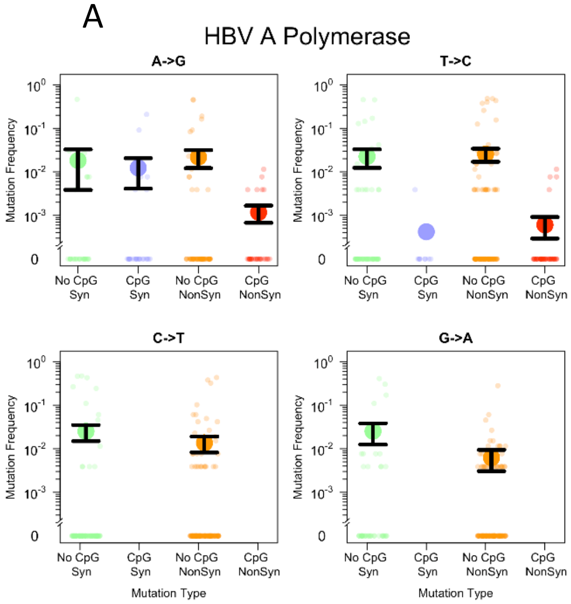

B

HBV A Polymerase

Mutation Type	Comparison	P−Value
A−>G	Syn: CpG v NonCpG	0.389
	NonSyn: CpG v NonCpG	0.347
	Syn v NonSyn	0.192
T−>C	Syn: CpG v NonCpG	0.15
	NonSyn: CpG v NonCpG	< 0.01
	Syn v NonSyn	0.41

Supplementary Fig 32. (a) Transition mutation frequencies for the analysis of HBV A Polymerase with mean and standard error (black line). (b) Wilcoxon test results, where the shade of the blue in the P-value cell represents the significance level; darker the shade, the more significant the results.

HBV A S

A

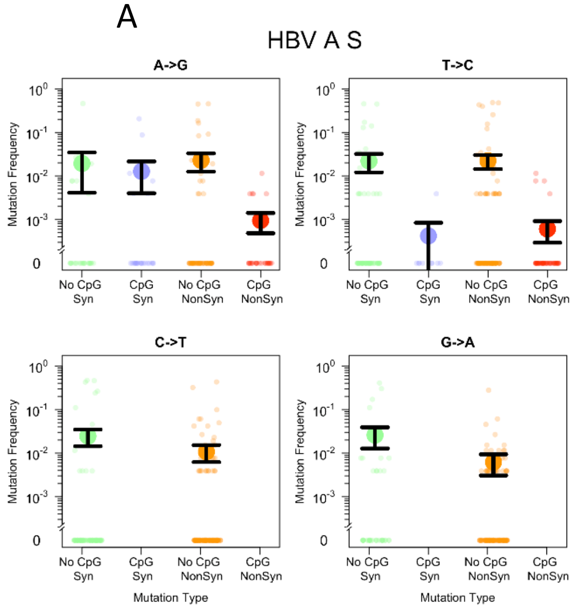

B

HBV A S

Mutation Type	Comparison	P–Value
A–>G	Syn: CpG v NonCpG	0.291
	NonSyn: CpG v NonCpG	0.262
	Syn v NonSyn	0.153
T–>C	Syn: CpG v NonCpG	0.155
	NonSyn: CpG v NonCpG	< 0.01
	Syn v NonSyn	0.392

Supplementary Fig 33. (a) Transition mutation frequencies for the analysis of HBV A S with mean and standard error (black line). (b) Wilcoxon test results, where the shade of the blue in the P-value cell represents the significance level; darker the shade, the more significant the results.

HBV B Polymerase

A

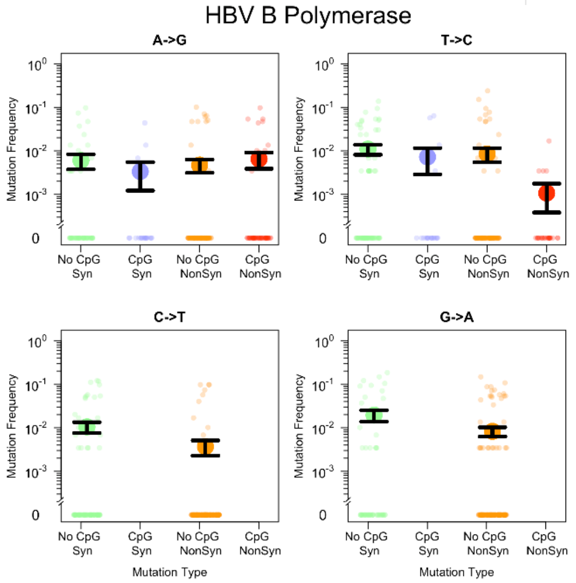

B

HBV B Polymerase

Mutation Type	Comparison	P−Value
A−>G	Syn: CpG v NonCpG	0.351
	NonSyn: CpG v NonCpG	0.832
	Syn v NonSyn	0.017
T−>C	Syn: CpG v NonCpG	0.154
	NonSyn: CpG v NonCpG	0.453
	Syn v NonSyn	< 0.01

Supplementary Fig 34. (a) Transition mutation frequencies for the analysis of HBV A Polymerase with mean and standard error (black line). (b) Wilcoxon test results, where the shade of the blue in the P-value cell represents the significance level; darker the shade, the more significant the results.

HBV B PreC-Core

A

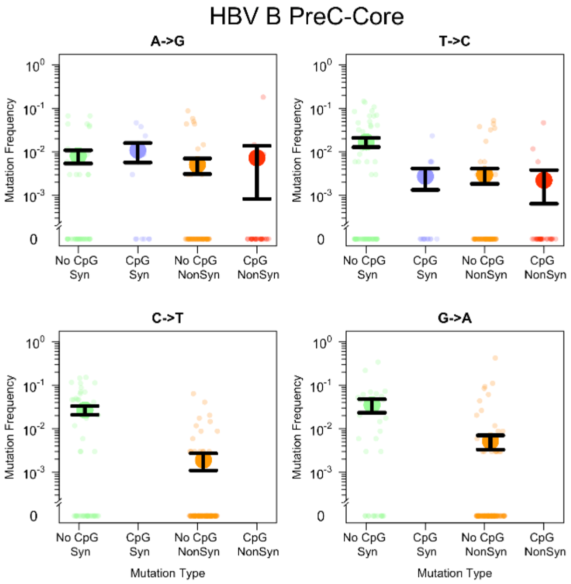

B

HBV B PreC−Core

Mutation Type	Comparison	P−Value
A−>G	Syn: CpG v NonCpG	0.764
	NonSyn: CpG v NonCpG	0.199
	Syn v NonSyn	< 0.01
T−>C	Syn: CpG v NonCpG	0.212
	NonSyn: CpG v NonCpG	0.526
	Syn v NonSyn	< 0.01

Supplementary Fig 35. (a) Transition mutation frequencies for the analysis of HBV B PreC-Core with mean and standard error (black line). (b) Wilcoxon test results, where the shade of the blue in the P-value cell represents the significance level; darker the shade, the more significant the results.

HBV C Polymerase

A

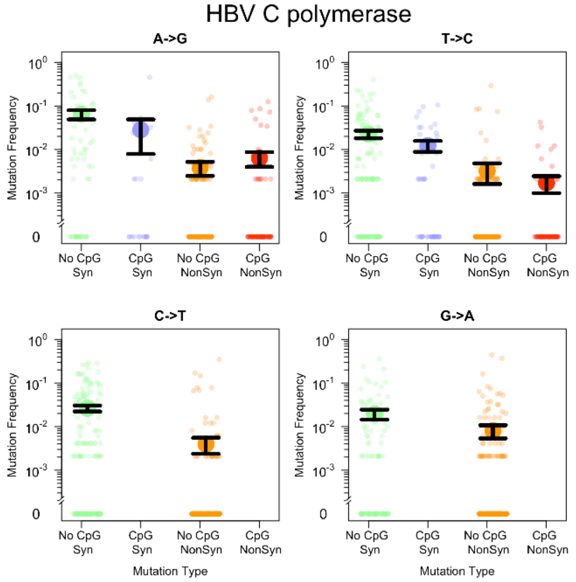

B

HBV C polymerase

Mutation Type	Comparison	P–Value
A–>G	Syn: CpG v NonCpG	0.0176
	NonSyn: CpG v NonCpG	0.218
	Syn v NonSyn	< 0.01
T–>C	Syn: CpG v NonCpG	0.212
	NonSyn: CpG v NonCpG	0.545
	Syn v NonSyn	< 0.01

Supplementary Fig 36. (a) Transition mutation frequencies for the analysis of HBV C Polymerase with mean and standard error (black line). (b) Wilcoxon test results, where the shade of the blue in the P-value cell represents the significance level; darker the shade, the more significant the results.

HBV C PreC-Core

A

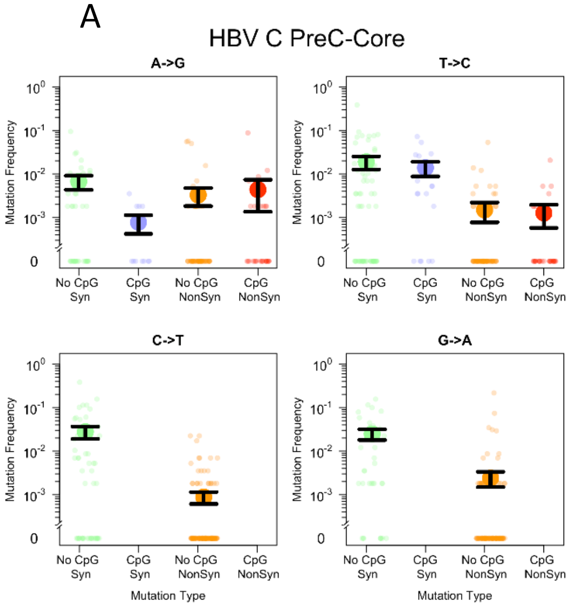

B

HBV C PreC−Core

Mutation Type	Comparison	P−Value
A−>G	Syn: CpG v NonCpG	0.047
	NonSyn: CpG v NonCpG	0.876
	Syn v NonSyn	< 0.01
T−>C	Syn: CpG v NonCpG	0.788
	NonSyn: CpG v NonCpG	0.599
	Syn v NonSyn	< 0.01

Supplementary Fig 37. (a) Transition mutation frequencies for the analysis of HBV C PreC-Core with mean and standard error (black line). (b) Wilcoxon test results, where the shade of the blue in the P-value cell represents the significance level; darker the shade, the more significant the results.

HBV C S

A

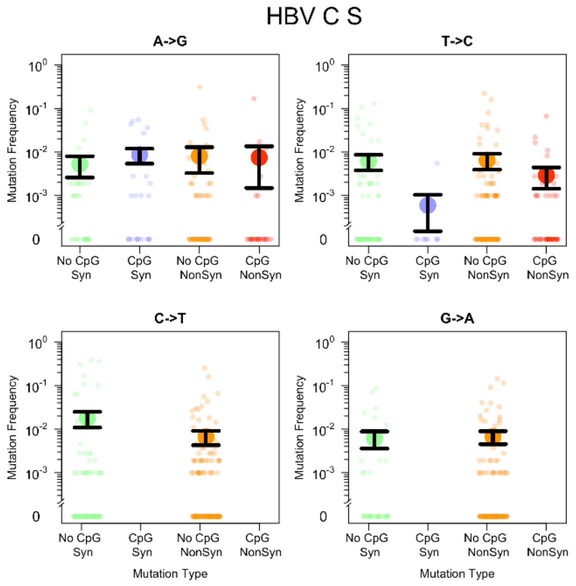

B

HBV C S

Mutation Type	Comparison	P–Value
A–>G	Syn: CpG v NonCpG	0.646
	NonSyn: CpG v NonCpG	0.153
	Syn v NonSyn	0.0179
T–>C	Syn: CpG v NonCpG	0.249
	NonSyn: CpG v NonCpG	0.213
	Syn v NonSyn	0.82

Supplementary Fig 38. (a) Transition mutation frequencies for the analysis of HBV C S with mean and standard error (black line). (b) Wilcoxon test results, where the shade of the blue in the P-value cell represents the significance level; darker the shade, the more significant the results.

Herpes 2 Glycoprotein G

A

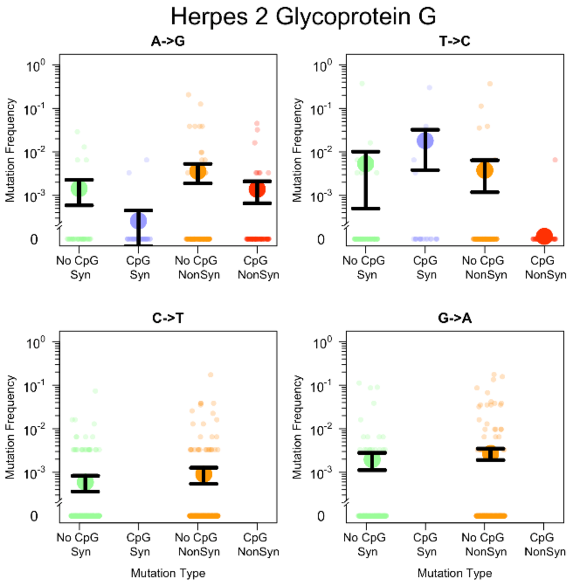

B

Herpes 2 Glycoprotein G

Mutation Type	Comparison	P−Value
A−>G	Syn: CpG v NonCpG	0.193
	NonSyn: CpG v NonCpG	0.529
	Syn v NonSyn	0.678
T−>C	Syn: CpG v NonCpG	0.972
	NonSyn: CpG v NonCpG	0.268
	Syn v NonSyn	< 0.01

Supplementary Fig 39. (a) Transition mutation frequencies for the analysis of Herpes 2 Glycoprotein G with mean and standard error (black line). (b) Wilcoxon test results, where the shade of the blue in the P-value cell represents the significance level; darker the shade, the more significant the results.

Human Papilloma 16 L1

A

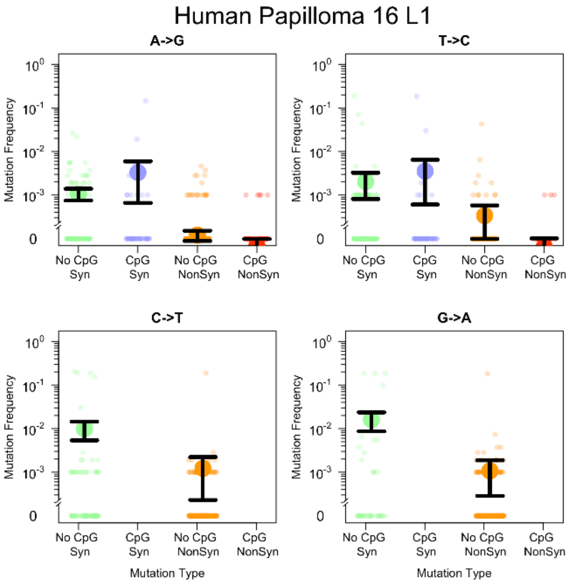

B

Human Papilloma 16 L1

Mutation Type	Comparison	P−Value
A−>G	Syn: CpG v NonCpG	0.0965
	NonSyn: CpG v NonCpG	0.456
	Syn v NonSyn	< 0.01
T−>C	Syn: CpG v NonCpG	0.102
	NonSyn: CpG v NonCpG	0.577
	Syn v NonSyn	< 0.01

Supplementary Fig 40. (a) Transition mutation frequencies for the analysis of Human Papilloma 16 L1 with mean and standard error (black line). (b) Wilcoxon test results, where the shade of the blue in the P-value cell represents the significance level; darker the shade, the more significant the results.

Parvo B19 NS1

A

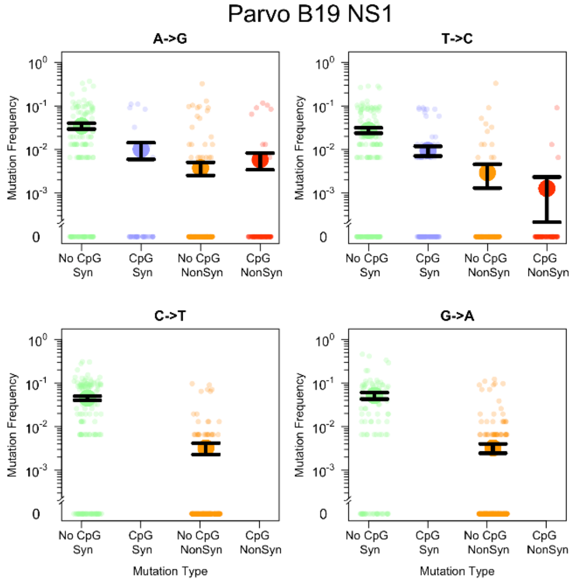

B

Parvo B19 NS1

Mutation Type	Comparison	P–Value
A–>G	Syn: CpG v NonCpG	< 0.01
	NonSyn: CpG v NonCpG	0.805
	Syn v NonSyn	< 0.01
T–>C	Syn: CpG v NonCpG	< 0.01
	NonSyn: CpG v NonCpG	0.22
	Syn v NonSyn	< 0.01

Supplementary Fig 41. (a) Transition mutation frequencies for the analysis of Parvo B19 NS1 with mean and standard error (black line). (b) Wilcoxon test results, where the shade of the blue in the P-value cell represents the significance level; darker the shade, the more significant the results.

Parvo B19 VP1

A

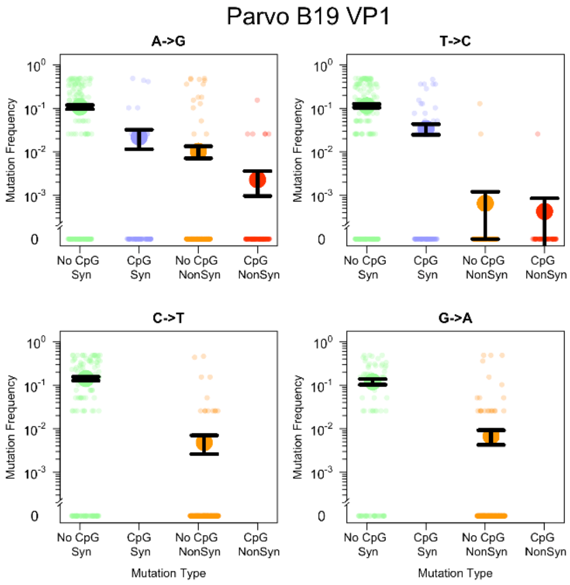

B

Parvo B19 VP1

Mutation Type	Comparison	P−Value
A−>G	Syn: CpG v NonCpG	< 0.01
	NonSyn: CpG v NonCpG	0.645
	Syn v NonSyn	< 0.01
T−>C	Syn: CpG v NonCpG	< 0.01
	NonSyn: CpG v NonCpG	0.711
	Syn v NonSyn	< 0.01

Supplementary Fig 42. (a) Transition mutation frequencies for the analysis of Parvo B19 VP1 with mean and standard error (black line). (b) Wilcoxon test results, where the shade of the blue in the P-value cell represents the significance level; darker the shade, the more significant the results.

Ancestral Dengue 1

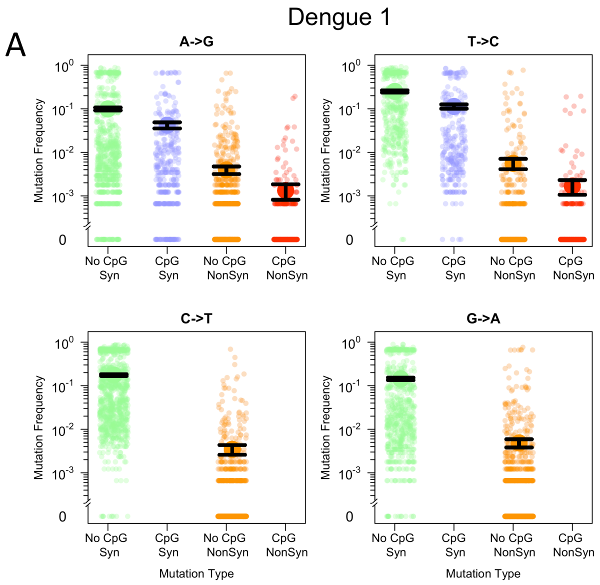

B

Dengue 1

Mutation Type	Comparison	P–Value
A–>G	Syn: CpG v NonCpG	< 0.01
	NonSyn: CpG v NonCpG	< 0.01
	Syn v NonSyn	< 0.01
T–>C	Syn: CpG v NonCpG	< 0.01
	NonSyn: CpG v NonCpG	0.0107
	Syn v NonSyn	< 0.01

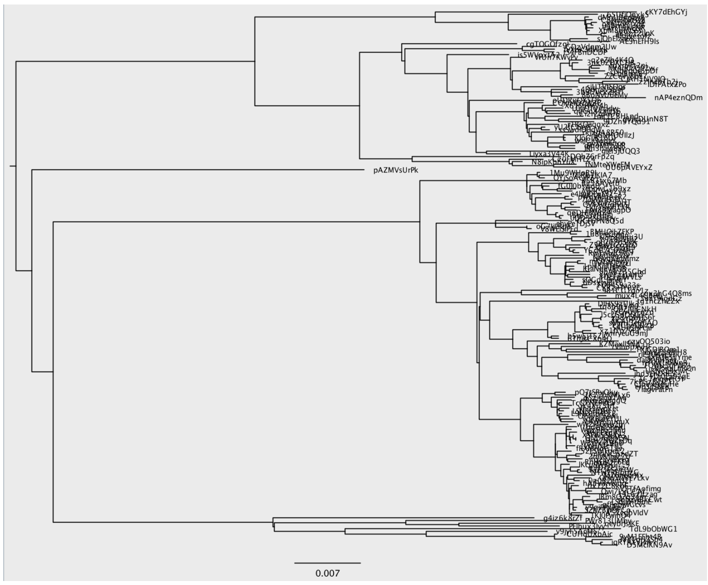

Supplementary Fig. 43 (a) Transition mutation frequencies for the ancestral analysis of Dengue 1 with mean and standard error (black line). (b) Wilcoxon test results, where the shade of the blue in the P-value cell represents the significance level; darker the shade, the more significant the results. (c) Midpoint rooted tree from a sample size of 200.

Ancestral Dengue 2

A

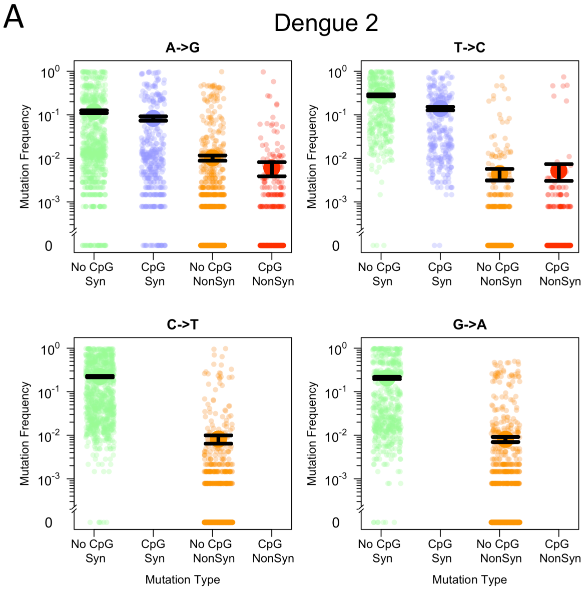

B

Dengue 2

Mutation Type	Comparison	P–Value
A–>G	Syn: CpG v NonCpG	< 0.01
	NonSyn: CpG v NonCpG	0.0174
	Syn v NonSyn	< 0.01
T–>C	Syn: CpG v NonCpG	< 0.01
	NonSyn: CpG v NonCpG	0.0198
	Syn v NonSyn	< 0.01

C

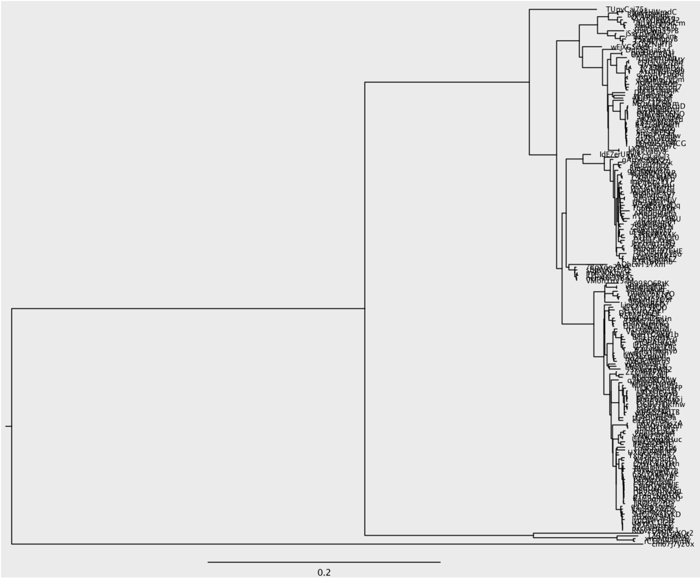

Supplementary Fig. 44 (a) Transition mutation frequencies for the ancestral analysis of Dengue 2 with mean and standard error (black line). (b) Wilcoxon test results, where the shade of the blue in the P-value cell represents the significance level; darker the shade, the more significant the results. (c) Midpoint rooted tree from a sample size of 200.

Ancestral Dengue 3

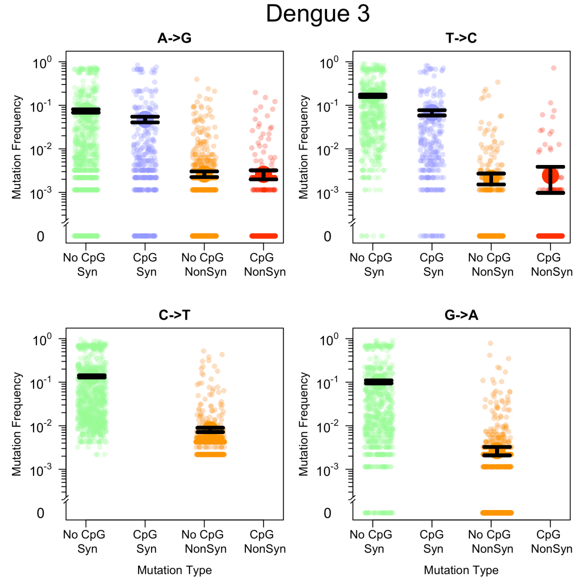

B

Dengue 3

Mutation Type	Comparison	P–Value
A–>G	Syn: CpG v NonCpG	< 0.01
	NonSyn: CpG v NonCpG	0.0677
	Syn v NonSyn	< 0.01
T–>C	Syn: CpG v NonCpG	< 0.01
	NonSyn: CpG v NonCpG	0.0168
	Syn v NonSyn	< 0.01

C

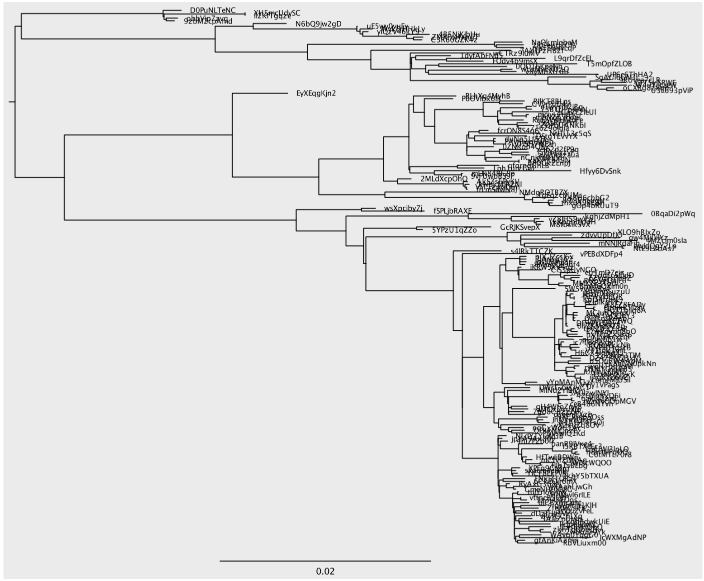

Supplementary Fig. 45 (a) Transition mutation frequencies for the ancestral analysis of Dengue 3 with mean and standard error (black line). (b) Wilcoxon test results, where the shade of the blue in the P-value cell represents the significance level; darker the shade, the more significant the results. (c) Midpoint rooted tree from a sample size of 200.

Ancestral Dengue 4

A

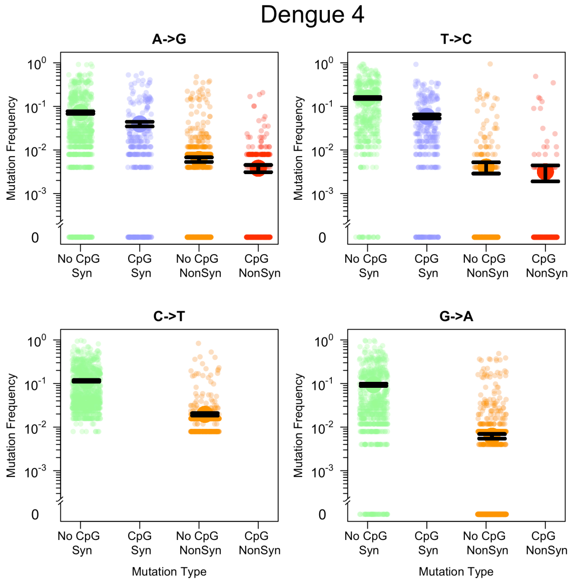

B

Dengue 4

Mutation Type	Comparison	P–Value
A–>G	Syn: CpG v NonCpG	< 0.01
	NonSyn: CpG v NonCpG	0.0575
	Syn v NonSyn	< 0.01
T–>C	Syn: CpG v NonCpG	< 0.01
	NonSyn: CpG v NonCpG	0.0572
	Syn v NonSyn	< 0.01

C

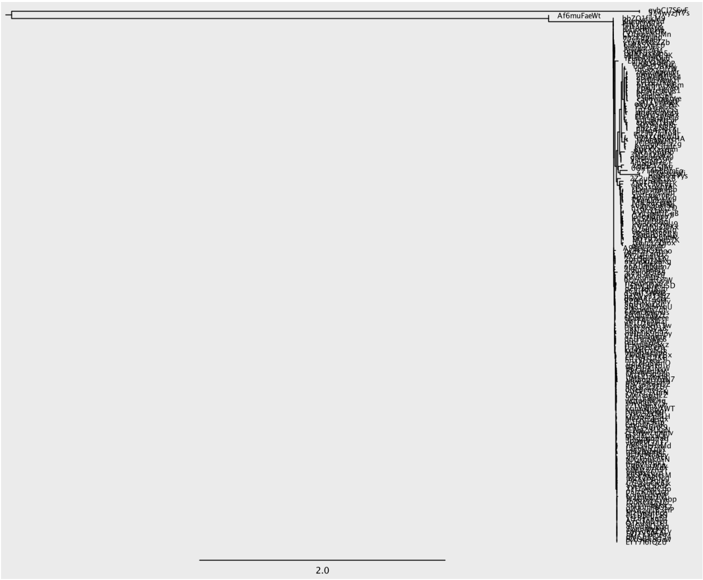

Supplementary Fig. 46 (a) Transition mutation frequencies for the ancestral analysis of Dengue 4 with mean and standard error (black line). (b) Wilcoxon test results, where the shade of the blue in the P-value cell represents the significance level; darker the shade, the more significant the results. (c) Midpoint rooted tree from a sample size of 200.

Ancestral HCV 1 A

A

HCV 1 A

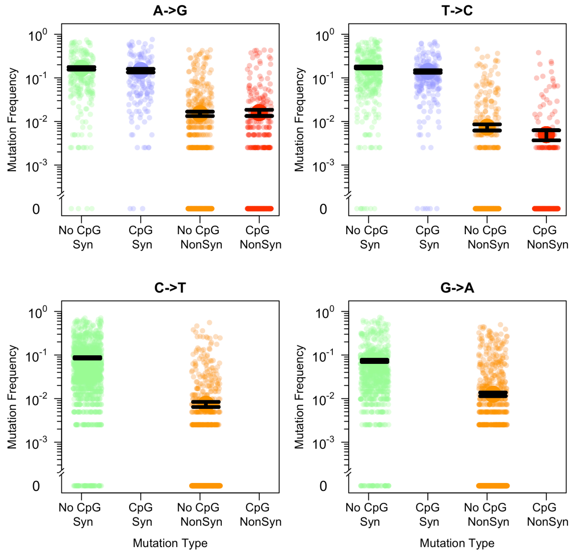

B

HCV 1 A

Mutation Type	Comparison	P–Value
A–>G	Syn: CpG v NonCpG	0.0649
	NonSyn: CpG v NonCpG	0.602
	Syn v NonSyn	< 0.01
T–>C	Syn: CpG v NonCpG	0.0143
	NonSyn: CpG v NonCpG	0.189
	Syn v NonSyn	< 0.01

C

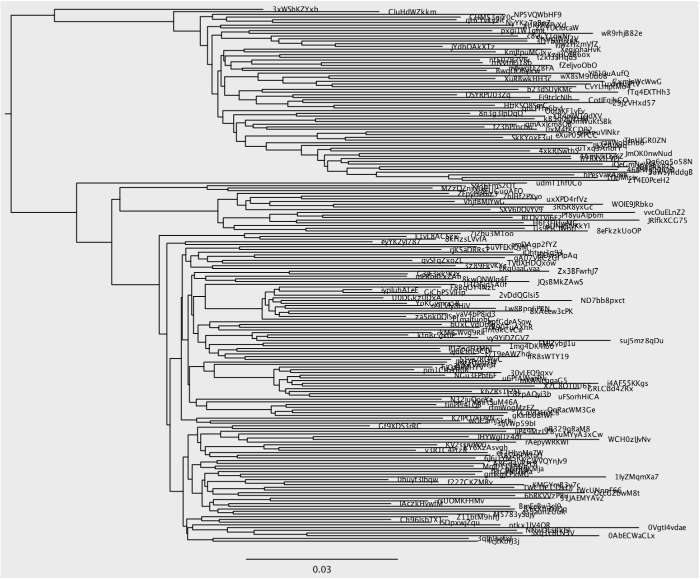

Supplementary Fig. 47 (a) Transition mutation frequencies for the ancestral analysis of HCV 1 A with mean and standard error (black line). (b) Wilcoxon test results, where the shade of the blue in the P-value cell represents the significance level; darker the shade, the more significant the results. (c) Midpoint rooted tree from a sample size of 200.

Ancestral HCV 1 B

A

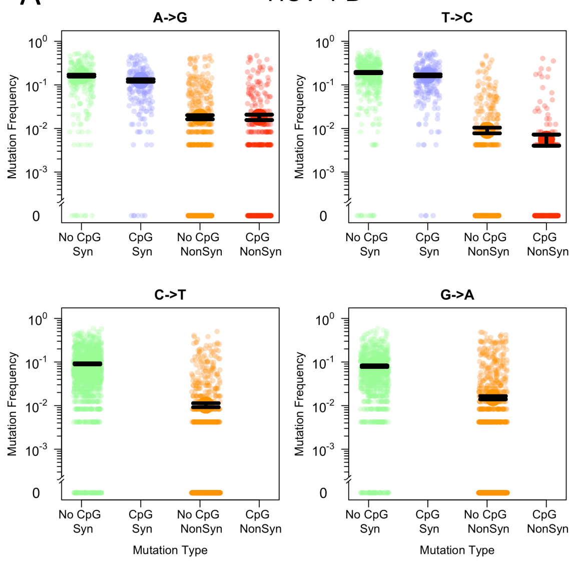

B

HCV 1 B

Mutation Type	Comparison	P–Value
A–>G	Syn: CpG v NonCpG	< 0.01
	NonSyn: CpG v NonCpG	0.841
	Syn v NonSyn	< 0.01
T–>C	Syn: CpG v NonCpG	< 0.01
	NonSyn: CpG v NonCpG	< 0.01
	Syn v NonSyn	< 0.01

C

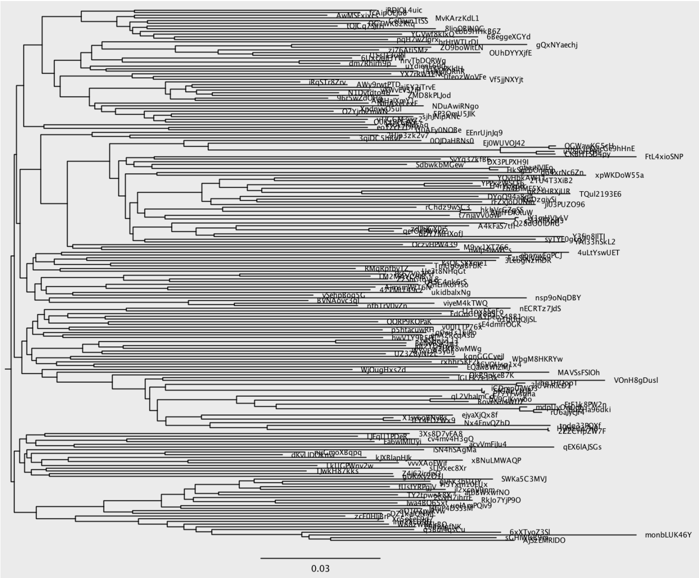

Supplementary Fig. 48(a) Transition mutation frequencies for the ancestral analysis of HCV 1 B with mean and standard error (black line). (b) Wilcoxon test results, where the shade of the blue in the P-value cell represents the significance level; darker the shade, the more significant the results. (c) Midpoint rooted tree from a sample size of 200.

Ancestral HIV pol Gene

A

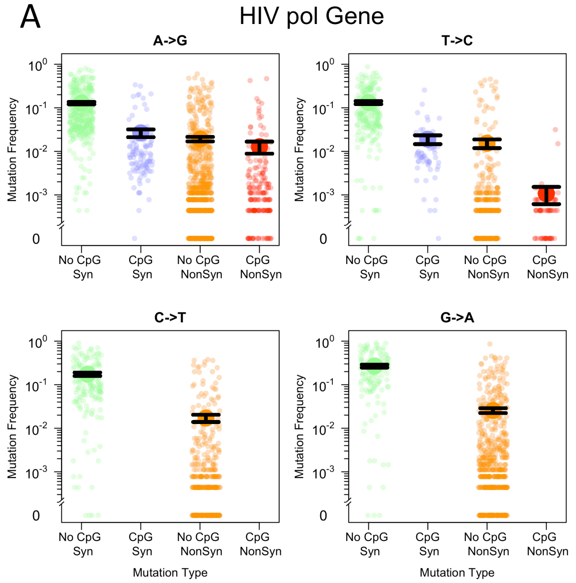

B

HIV pol Gene

Mutation Type	Comparison	P–Value
A–>G	Syn: CpG v NonCpG	< 0.01
	NonSyn: CpG v NonCpG	0.537
	Syn v NonSyn	< 0.01
T–>C	Syn: CpG v NonCpG	< 0.01
	NonSyn: CpG v NonCpG	0.0657
	Syn v NonSyn	< 0.01

C

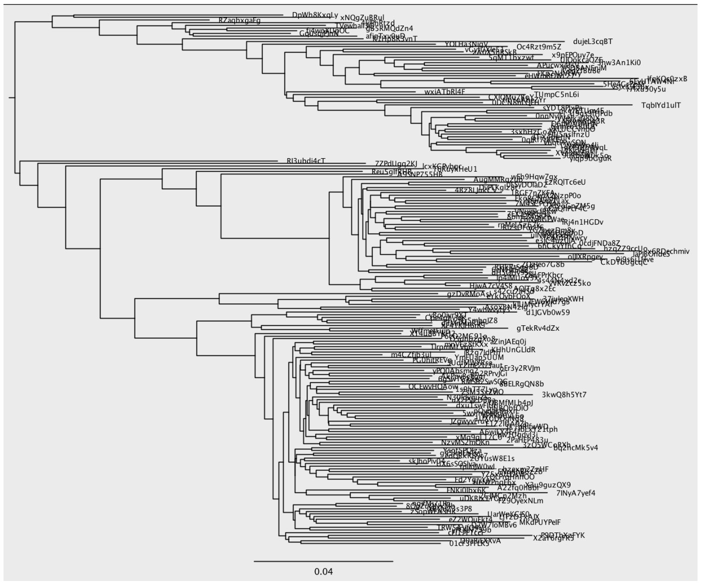

Supplementary Fig. 49 (a) Transition mutation frequencies for the ancestral analysis of HIV Pol gene with mean and standard error (black line). (b) Wilcoxon test results, where the shade of the blue in the P-value cell represents the significance level; darker the shade, the more significant the results. (c) Midpoint rooted tree from a sample size of 200.

Ancestral Human Parainfluenza 1 HN

A

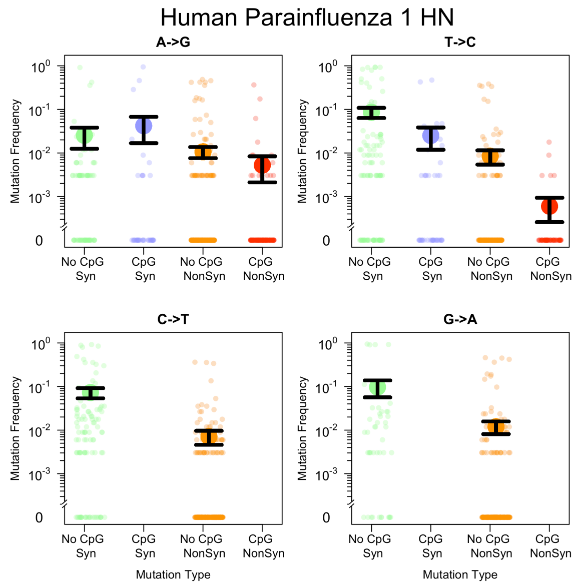

B

Human Parainfluenza 1 HN

Mutation Type	Comparison	P–Value
A–>G	Syn: CpG v NonCpG	< 0.01
	NonSyn: CpG v NonCpG	0.148
	Syn v NonSyn	< 0.01
T–>C	Syn: CpG v NonCpG	< 0.01
	NonSyn: CpG v NonCpG	0.0689
	Syn v NonSyn	< 0.01

C

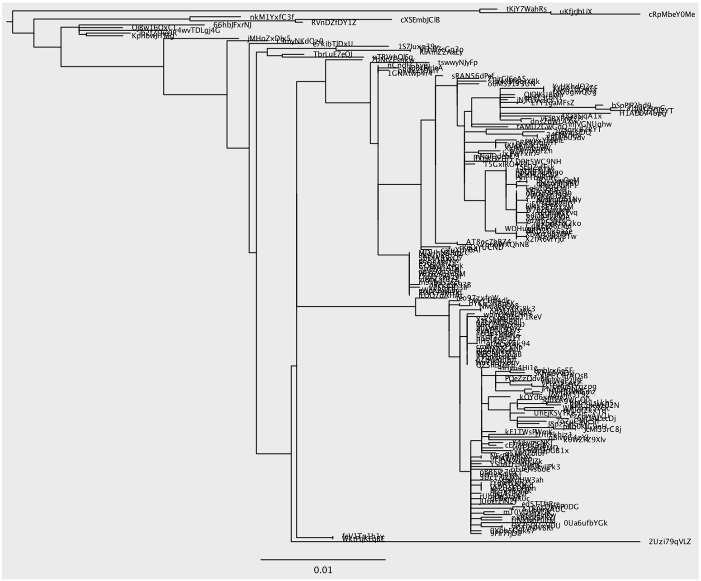

Supplementary Fig. 50 (a) Transition mutation frequencies for the ancestral analysis of Human Parainfluenza 1 HN with mean and standard error (black line). (b) Wilcoxon test results, where the shade of the blue in the P-value cell represents the significance level; darker the shade, the more significant the results. (c) Midpoint rooted tree from a sample size of 200.

Ancestral Human Parainfluenza 3 HN

A

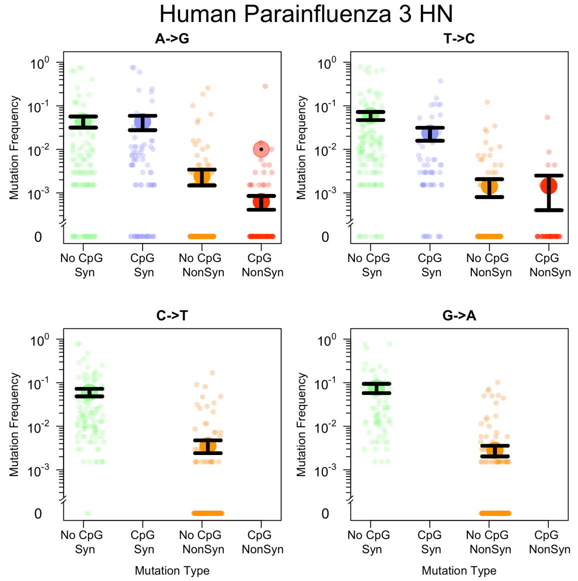

B

Human Parainfluenza 3 HN

Mutation Type	Comparison	P–Value
A–>G	Syn: CpG v NonCpG	0.256
	NonSyn: CpG v NonCpG	0.404
	Syn v NonSyn	< 0.01
T–>C	Syn: CpG v NonCpG	< 0.01
	NonSyn: CpG v NonCpG	0.715
	Syn v NonSyn	< 0.01

C

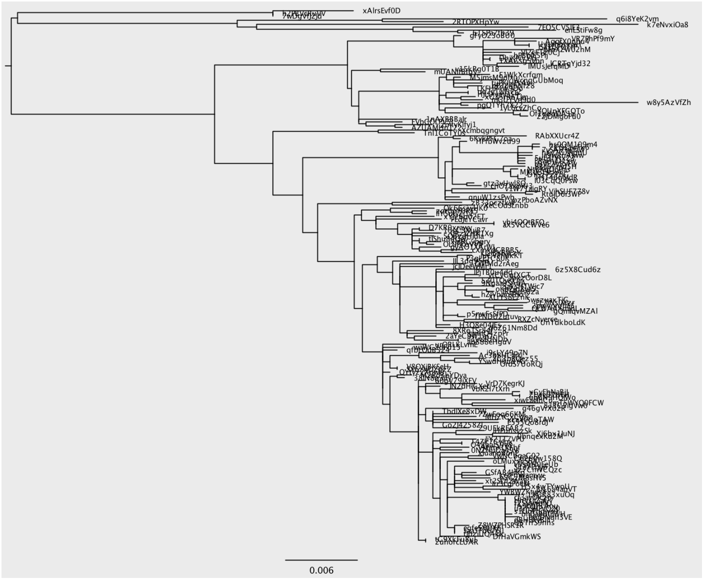

Supplementary Fig. 51 (a) Transition mutation frequencies for the ancestral analysis of Human Parainfluenza 3 HN with mean and standard error (black line). (b) Wilcoxon test results, where the shade of the blue in the P-value cell represents the significance level; darker the shade, the more significant the results. (c) Midpoint rooted tree from a sample size of 200.

Ancestral Human Parainfluenza 1

A

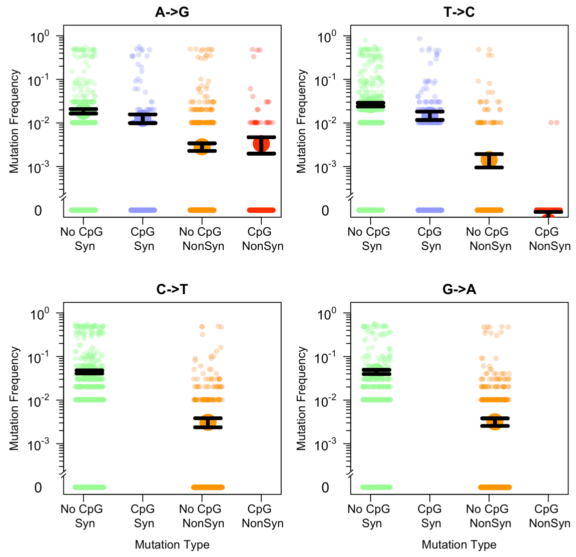

B

Human Parainfluenza 1

Mutation Type	Comparison	P–Value
A–>G	Syn: CpG v NonCpG	< 0.01
	NonSyn: CpG v NonCpG	0.413
	Syn v NonSyn	< 0.01
T–>C	Syn: CpG v NonCpG	< 0.01
	NonSyn: CpG v NonCpG	< 0.01
	Syn v NonSyn	< 0.01

C

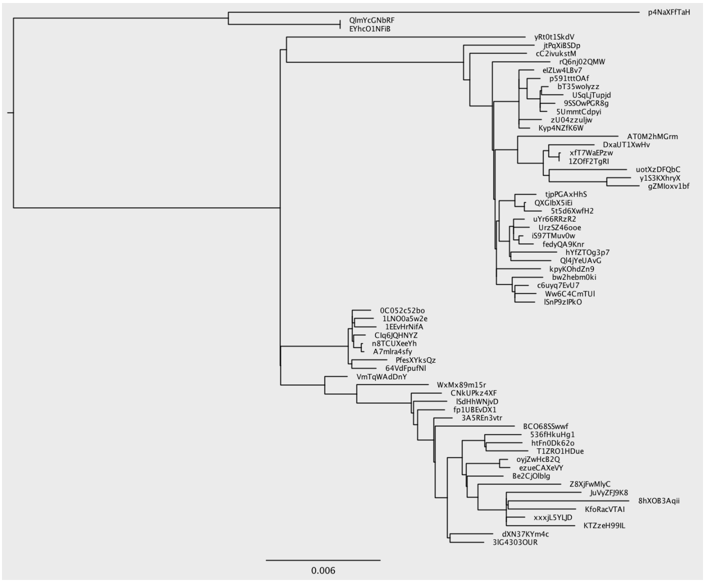

Supplementary Fig. 52 (a) Transition mutation frequencies for the ancestral analysis of Human Parainfluenza 1 with mean and standard error (black line). (b) Wilcoxon test results, where the shade of the blue in the P-value cell represents the significance level; darker the shade, the more significant the results. (c) Midpoint rooted tree from a sample size of 200.

Ancestral Human Parainfluenza 3

A

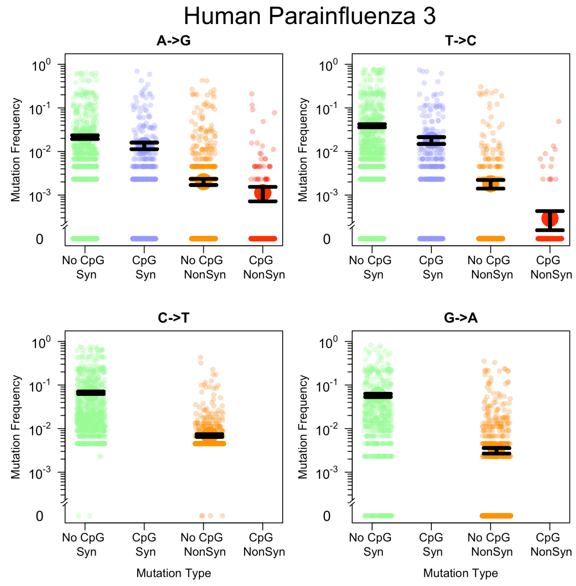

B

Human Parainfluenza 3

Mutation Type	Comparison	P–Value
A–>G	Syn: CpG v NonCpG	< 0.01
	NonSyn: CpG v NonCpG	< 0.01
	Syn v NonSyn	< 0.01
T–>C	Syn: CpG v NonCpG	< 0.01
	NonSyn: CpG v NonCpG	< 0.01
	Syn v NonSyn	< 0.01

C

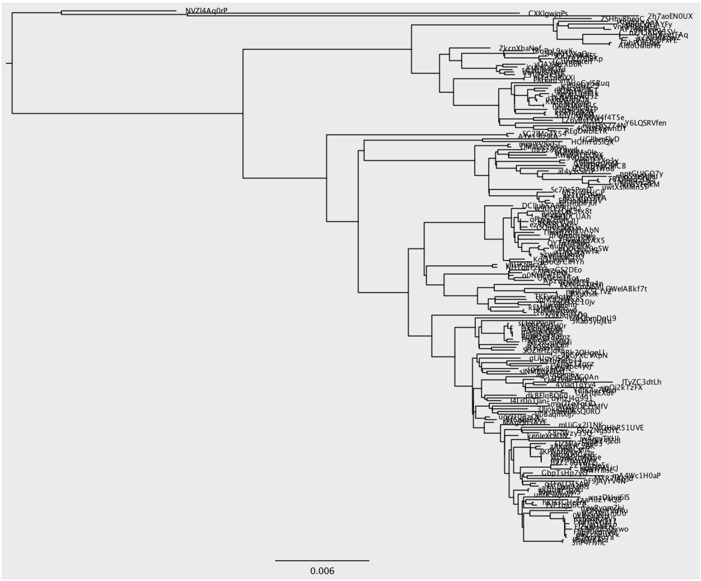

Supplementary Fig. 53 (a) Transition mutation frequencies for the ancestral analysis of Human Parainfluenza 3 with mean and standard error (black line). (b) Wilcoxon test results, where the shade of the blue in the P-value cell represents the significance level; darker the shade, the more significant the results. (c) Midpoint rooted tree from a sample size of 200.

Ancestral Influenza A HA H1N1

A

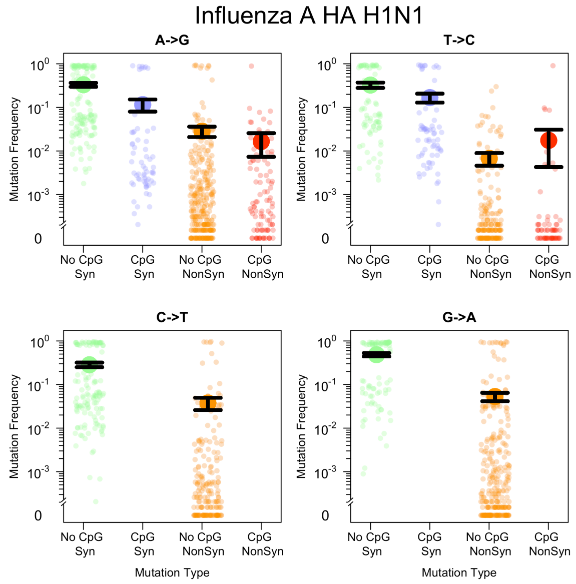

B

Influenza A HA H1N1

Mutation Type	Comparison	P−Value
A−>G	Syn: CpG v NonCpG	< 0.01
	NonSyn: CpG v NonCpG	0.499
	Syn v NonSyn	< 0.01
T−>C	Syn: CpG v NonCpG	< 0.01
	NonSyn: CpG v NonCpG	< 0.01
	Syn v NonSyn	< 0.01

C

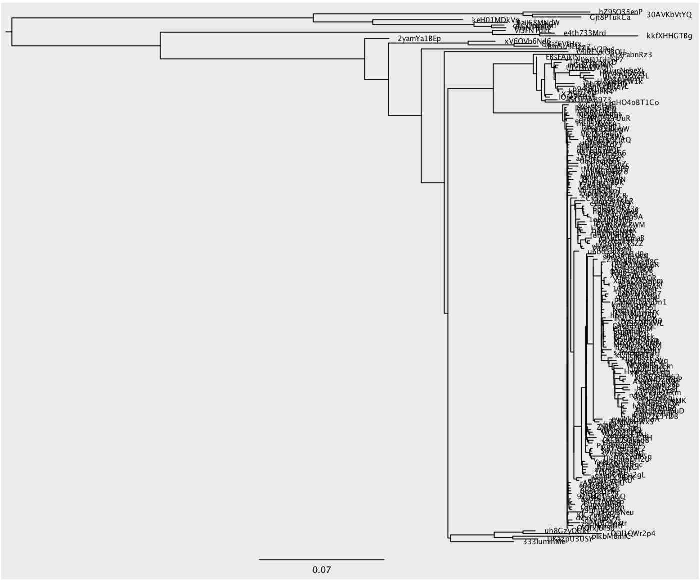

Supplementary Fig. 54 (a) Transition mutation frequencies for the ancestral analysis of Influenza A HA H1N1 with mean and standard error (black line). (b) Wilcoxon test results, where the shade of the blue in the P-value cell represents the significance level; darker the shade, the more significant the results. (c) Midpoint rooted tree from a sample size of 200.

Ancestral Influenza A HA H3N2

A

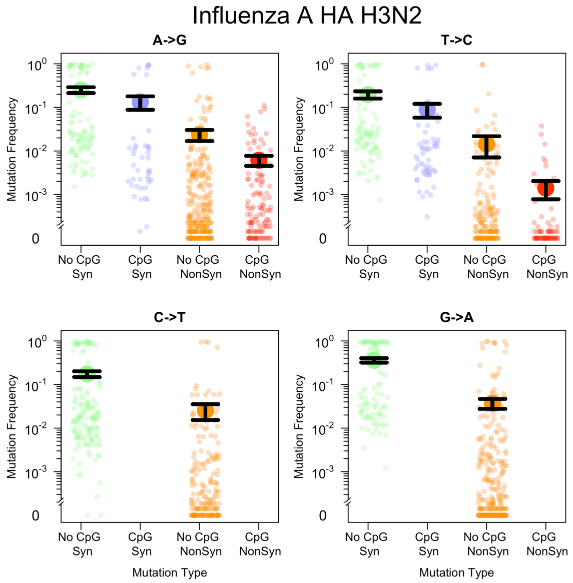

B

Influenza A HA H3N2

Mutation Type	Comparison	P–Value
A–>G	Syn: CpG v NonCpG	< 0.01
	NonSyn: CpG v NonCpG	0.486
	Syn v NonSyn	< 0.01
T–>C	Syn: CpG v NonCpG	< 0.01
	NonSyn: CpG v NonCpG	< 0.01
	Syn v NonSyn	< 0.01

C

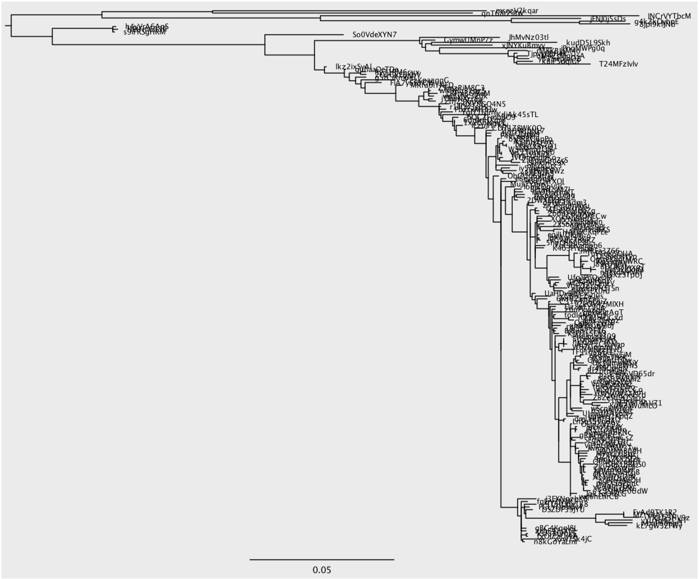

Supplementary Fig. 55 (a) Transition mutation frequencies for the ancestral analysis of Influenza A HA H3N2 with mean and standard error (black line). (b) Wilcoxon test results, where the shade of the blue in the P-value cell represents the significance level; darker the shade, the more significant the results. (c) Midpoint rooted tree from a sample size of 200.

Ancestral Influenza A NA H1N1

A

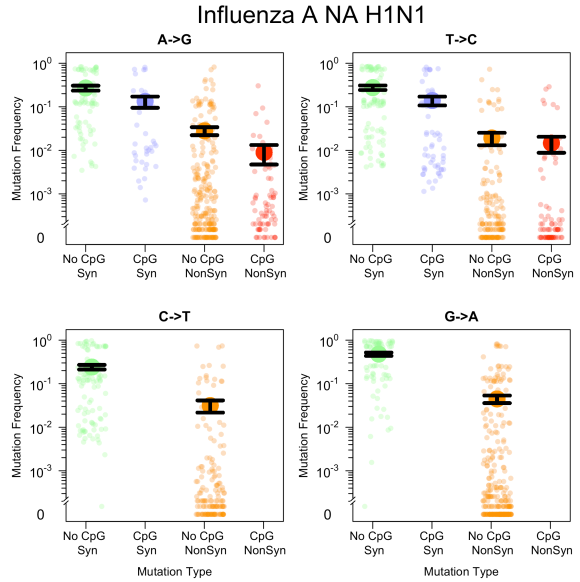

B

Influenza A NA H1N1

Mutation Type	Comparison	P–Value
A–>G	Syn: CpG v NonCpG	< 0.01
	NonSyn: CpG v NonCpG	0.331
	Syn v NonSyn	< 0.01
T–>C	Syn: CpG v NonCpG	< 0.01
	NonSyn: CpG v NonCpG	0.0188
	Syn v NonSyn	< 0.01

C

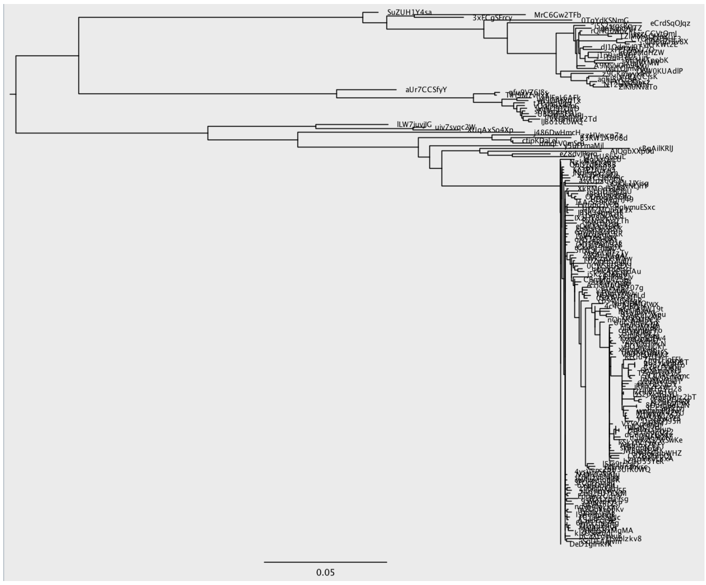

Supplementary Fig. 56 (a) Transition mutation frequencies for the ancestral analysis of Influenza A NA H1N1 with mean and standard error (black line). (b) Wilcoxon test results, where the shade of the blue in the P-value cell represents the significance level; darker the shade, the more significant the results. (c) Midpoint rooted tree from a sample size of 200.

Ancestral Influenza A NA H3N2

A

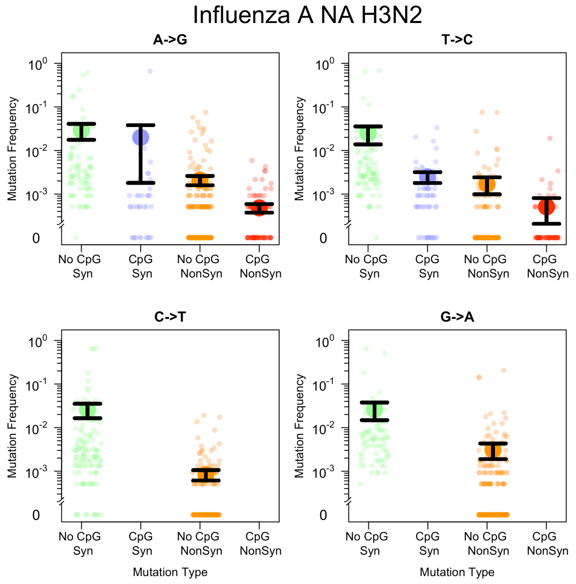

B

Influenza A NA H3N2

Mutation Type	Comparison	P−Value
A−>G	Syn: CpG v NonCpG	< 0.01
	NonSyn: CpG v NonCpG	0.229
	Syn v NonSyn	< 0.01
T−>C	Syn: CpG v NonCpG	< 0.01
	NonSyn: CpG v NonCpG	0.176
	Syn v NonSyn	< 0.01

C

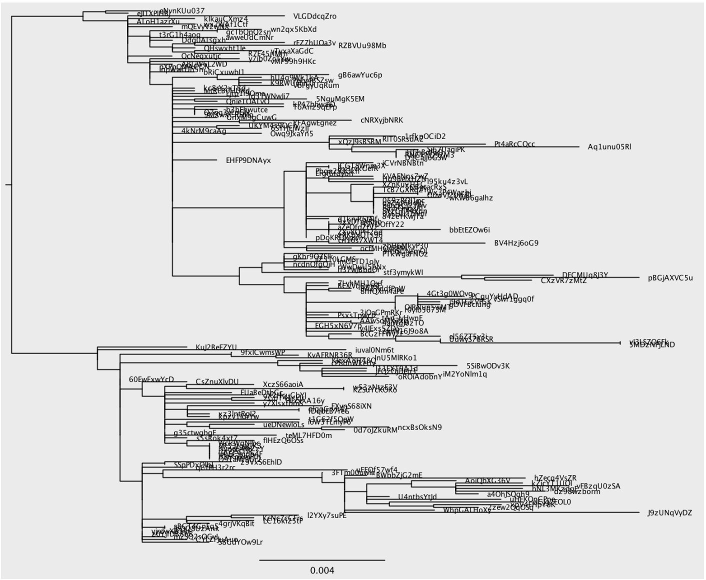

Supplementary Fig. 57 (a) Transition mutation frequencies for the ancestral analysis of Influenza A NA H3N2 with mean and standard error (black line). (b) Wilcoxon test results, where the shade of the blue in the P-value cell represents the significance level; darker the shade, the more significant the results. (c) Midpoint rooted tree from a sample size of 200.

Ancestral Influenza B HA

A

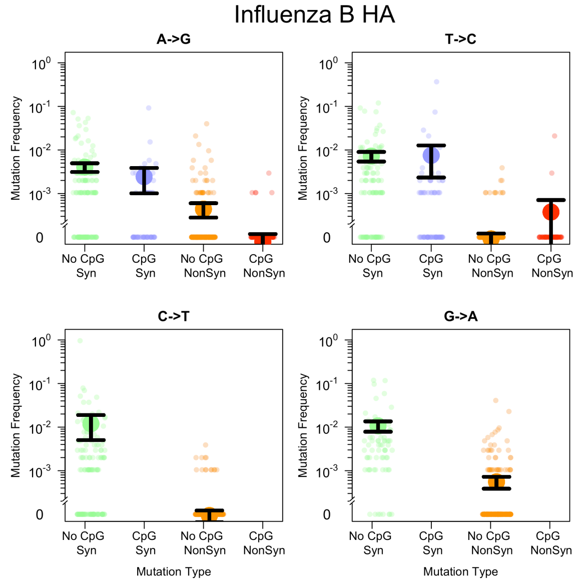

B

Influenza B HA

Mutation Type	Comparison	P–Value
A–>G	Syn: CpG v NonCpG	< 0.01
	NonSyn: CpG v NonCpG	0.0928
	Syn v NonSyn	< 0.01
T–>C	Syn: CpG v NonCpG	< 0.01
	NonSyn: CpG v NonCpG	0.162
	Syn v NonSyn	< 0.01

C

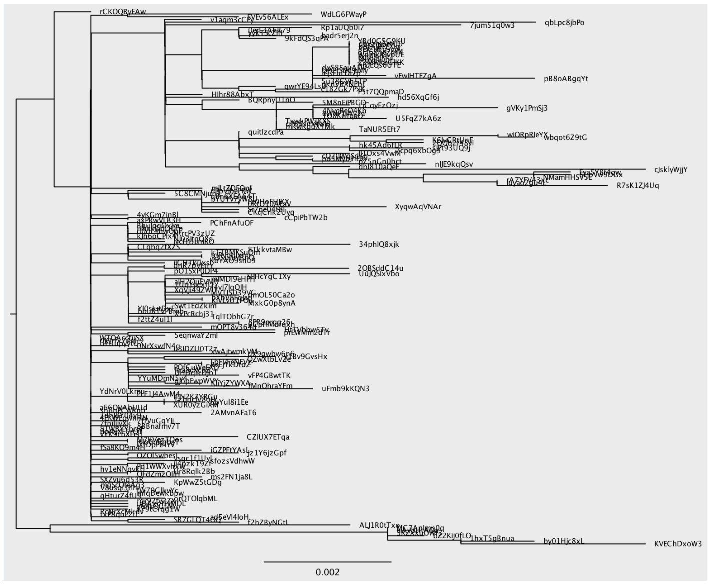

Supplementary Fig. 58 (a) Transition mutation frequencies for the ancestral analysis of Influenza B HA with mean and standard error (black line). (b) Wilcoxon test results, where the shade of the blue in the P-value cell represents the significance level; darker the shade, the more significant the results. (c) Midpoint rooted tree from a sample size of 200.

Ancestral Influenza B NA

A

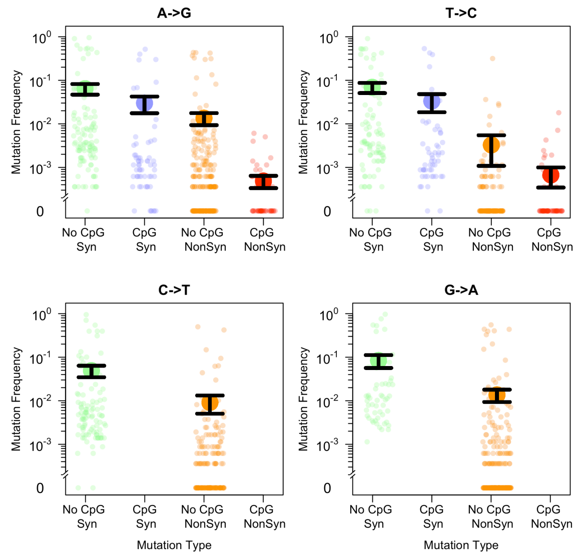

B

Influenza B NA

Mutation Type	Comparison	P–Value
A–>G	Syn: CpG v NonCpG	< 0.01
	NonSyn: CpG v NonCpG	0.0296
	Syn v NonSyn	< 0.01
T–>C	Syn: CpG v NonCpG	< 0.01
	NonSyn: CpG v NonCpG	0.212
	Syn v NonSyn	< 0.01

C

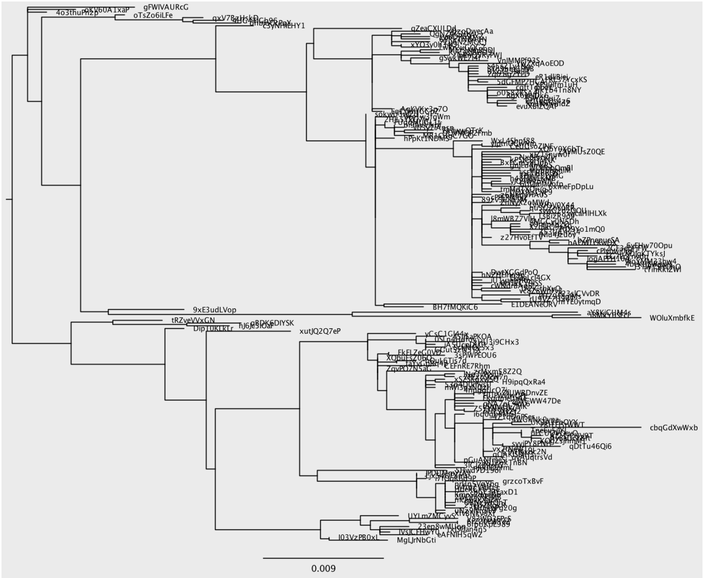

Supplementary Fig. 59 (a) Transition mutation frequencies for the ancestral analysis of Influenza B NA with mean and standard error (black line). (b) Wilcoxon test results, where the shade of the blue in the P-value cell represents the significance level; darker the shade, the more significant the results. (c) Midpoint rooted tree from a sample size of 200.

Ancestral Entero A VP1 EVA71

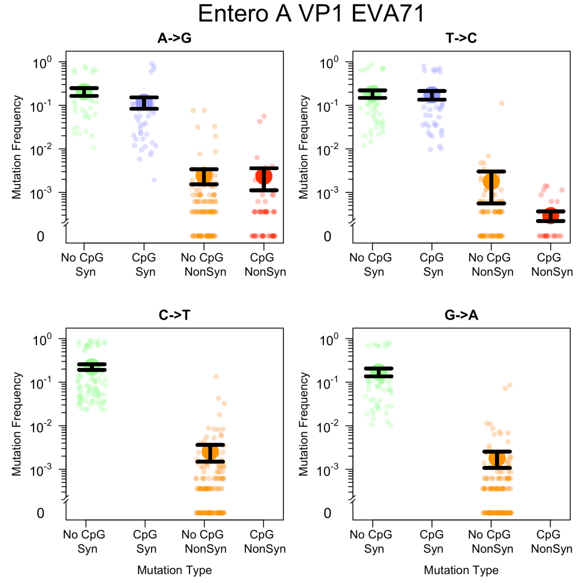

B

Entero A VP1 EVA71

Mutation Type	Comparison	P−Value
A−>G	Syn: CpG v NonCpG	< 0.01
	NonSyn: CpG v NonCpG	0.185
	Syn v NonSyn	< 0.01
T−>C	Syn: CpG v NonCpG	0.187
	NonSyn: CpG v NonCpG	0.188
	Syn v NonSyn	< 0.01

C

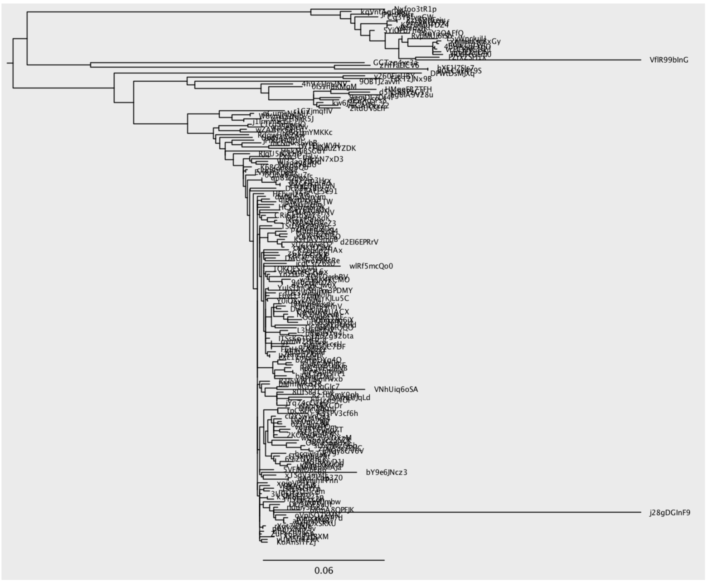

Supplementary Fig. 60 (a) Transition mutation frequencies for the ancestral analysis of Entero A VP1 EVA71 with mean and standard error (black line). (b) Wilcoxon test results, where the shade of the blue in the P-value cell represents the significance level; darker the shade, the more significant the results. (c) Midpoint rooted tree from a sample size of 200.

Ancestral Entero A VP2 EVA71

A

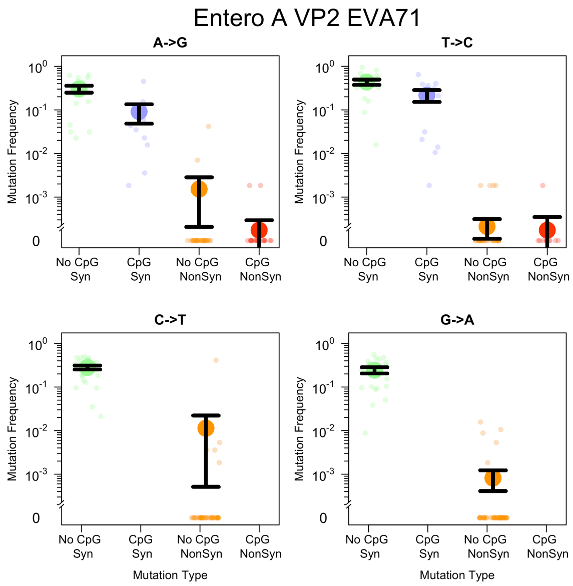

B

Entero A VP2 EVA71

Mutation Type	Comparison	P–Value
A–>G	Syn: CpG v NonCpG	< 0.01
	NonSyn: CpG v NonCpG	0.665
	Syn v NonSyn	< 0.01
T–>C	Syn: CpG v NonCpG	0.011
	NonSyn: CpG v NonCpG	0.438
	Syn v NonSyn	< 0.01

C

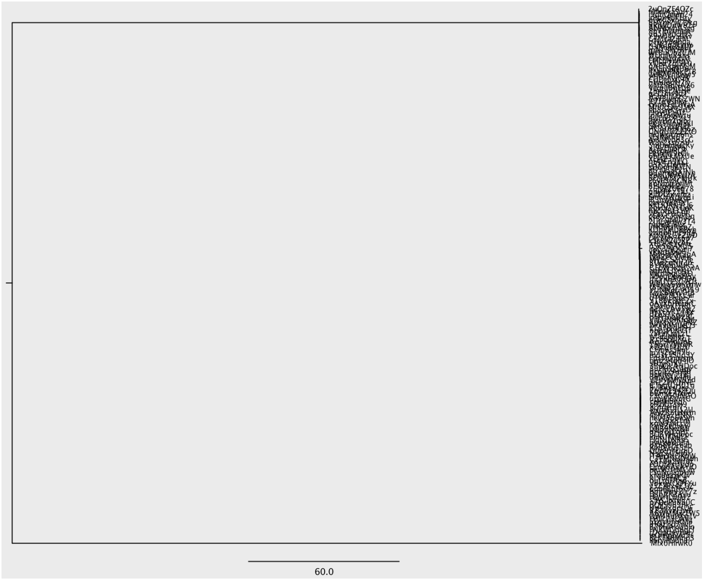

Supplementary Fig. 61 (a) Transition mutation frequencies for the ancestral analysis of Entero A VP2 EVA71 with mean and standard error (black line). (b) Wilcoxon test results, where the shade of the blue in the P-value cell represents the significance level; darker the shade, the more significant the results. (c) Midpoint rooted tree from a sample size of 200.

Ancestral Entero B VP1 Echovirus30

A

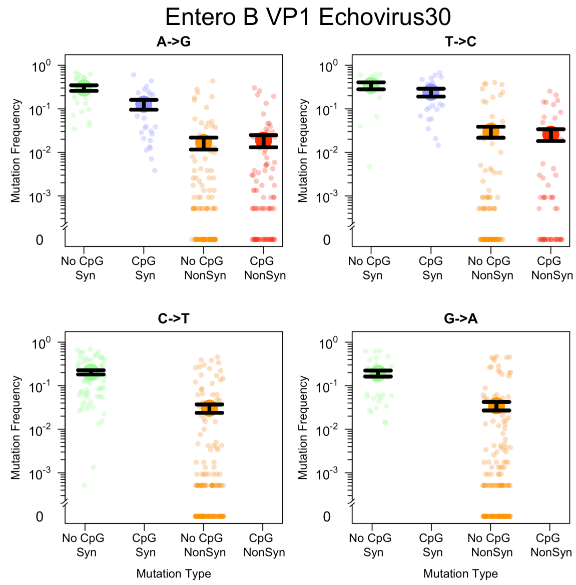

B

Entero B VP1 Echovirus30

Mutation Type	Comparison	P–Value
A–>G	Syn: CpG v NonCpG	< 0.01
	NonSyn: CpG v NonCpG	0.985
	Syn v NonSyn	< 0.01
T–>C	Syn: CpG v NonCpG	0.125
	NonSyn: CpG v NonCpG	0.858
	Syn v NonSyn	< 0.01

C

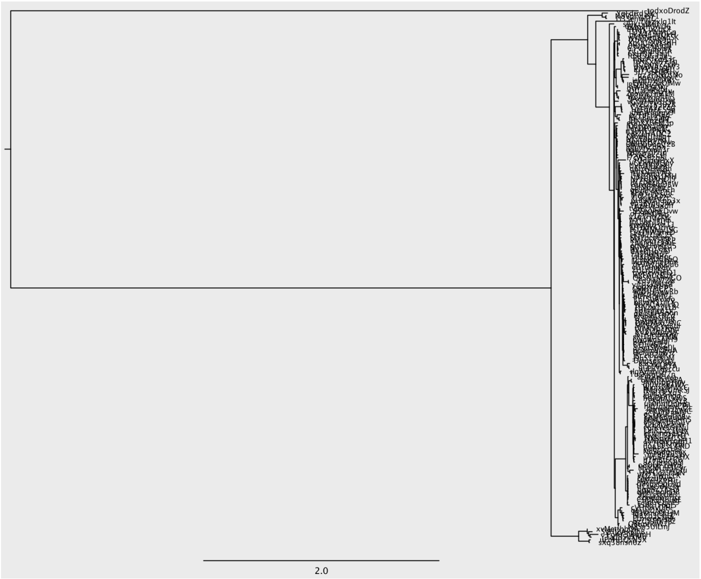

Supplementary Fig. 62 (a) Transition mutation frequencies for the ancestral analysis of Entero B VP1 Echovirus30 with mean and standard error (black line). (b) Wilcoxon test results, where the shade of the blue in the P-value cell represents the significance level; darker the shade, the more significant the results. (c) Midpoint rooted tree from a sample size of 200.

Ancestral Entero B VP2 Echovirus30

A

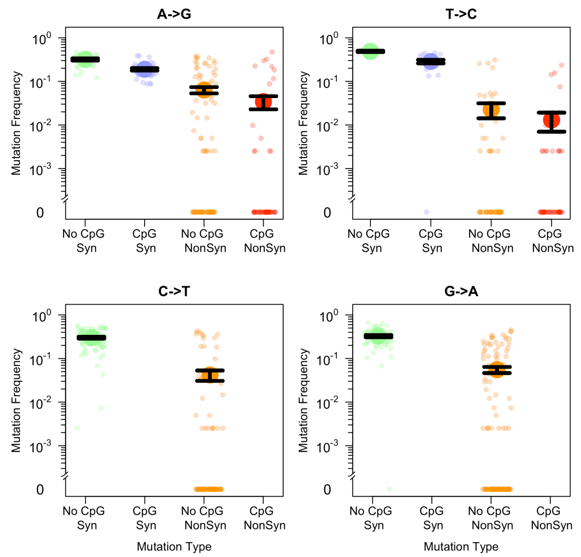

B

Entero B VP2 Echovirus30

Mutation Type	Comparison	P–Value
A–>G	Syn: CpG v NonCpG	< 0.01
	NonSyn: CpG v NonCpG	< 0.01
	Syn v NonSyn	< 0.01
T–>C	Syn: CpG v NonCpG	< 0.01
	NonSyn: CpG v NonCpG	0.215
	Syn v NonSyn	< 0.01

C

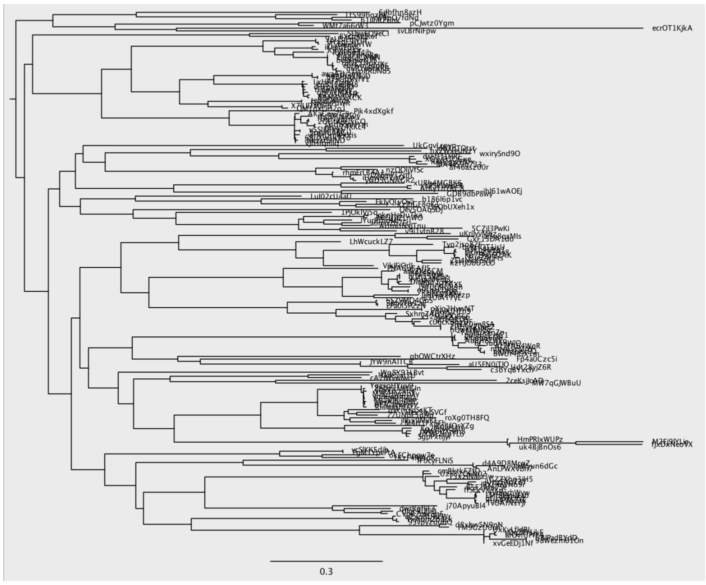

Supplementary Fig. 63 (a) Transition mutation frequencies for the ancestral analysis of Entero B VP2 Echovirus30 with mean and standard error (black line). (b) Wilcoxon test results, where the shade of the blue in the P-value cell represents the significance level; darker the shade, the more significant the results. (c) Midpoint rooted tree from a sample size of 200.

Ancestral Entero C VP1 Polio2

A

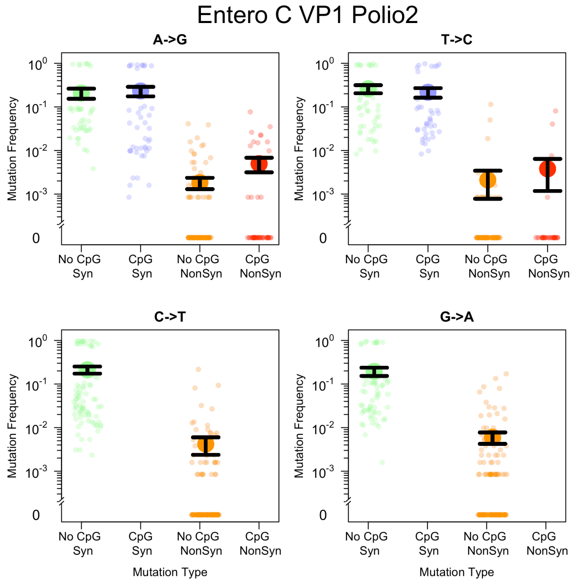

B

Entero C VP1 Polio2

Mutation Type	Comparison	P–Value
A–>G	Syn: CpG v NonCpG	0.0246
	NonSyn: CpG v NonCpG	0.555
	Syn v NonSyn	< 0.01
T–>C	Syn: CpG v NonCpG	0.131
	NonSyn: CpG v NonCpG	0.206
	Syn v NonSyn	< 0.01

C

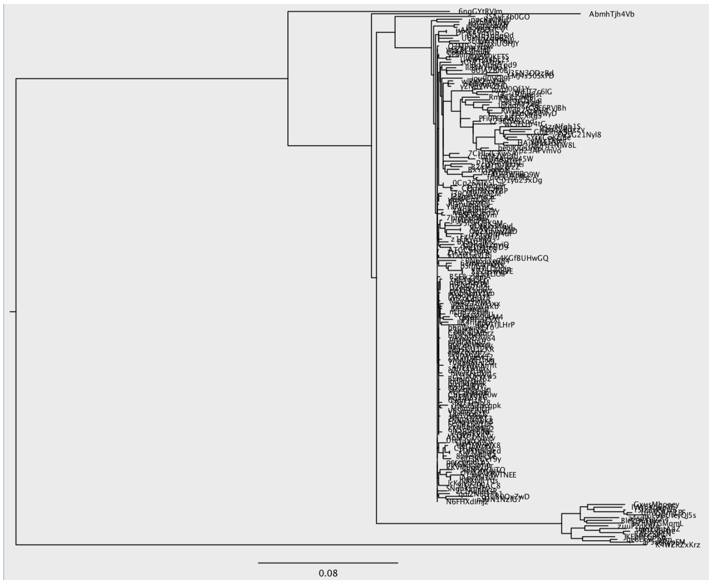

Supplementary Fig. 64 (a) Transition mutation frequencies for the ancestral analysis of Entero C VP1 Polio2 with mean and standard error (black line). (b) Wilcoxon test results, where the shade of the blue in the P-value cell represents the significance level; darker the shade, the more significant the results. (c) Midpoint rooted tree from a sample size of 200.

Ancestral Entero C VP2 Polio2

A

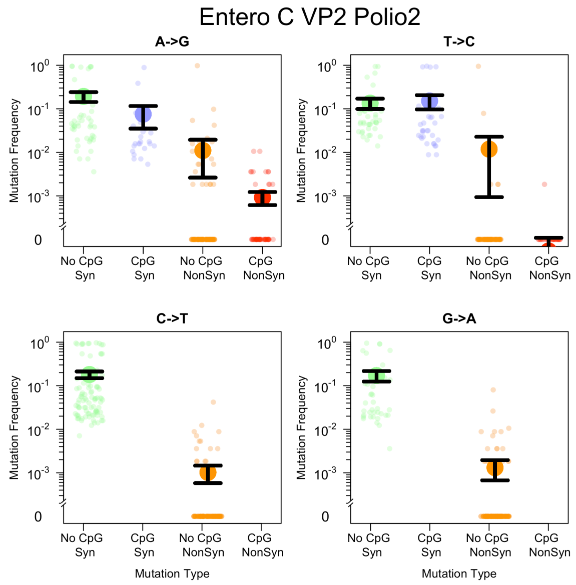

B

Entero C VP2 Polio2

Mutation Type	Comparison	P–Value
A–>G	Syn: CpG v NonCpG	< 0.01
	NonSyn: CpG v NonCpG	0.734
	Syn v NonSyn	< 0.01
T–>C	Syn: CpG v NonCpG	0.0134
	NonSyn: CpG v NonCpG	0.279
	Syn v NonSyn	< 0.01

C

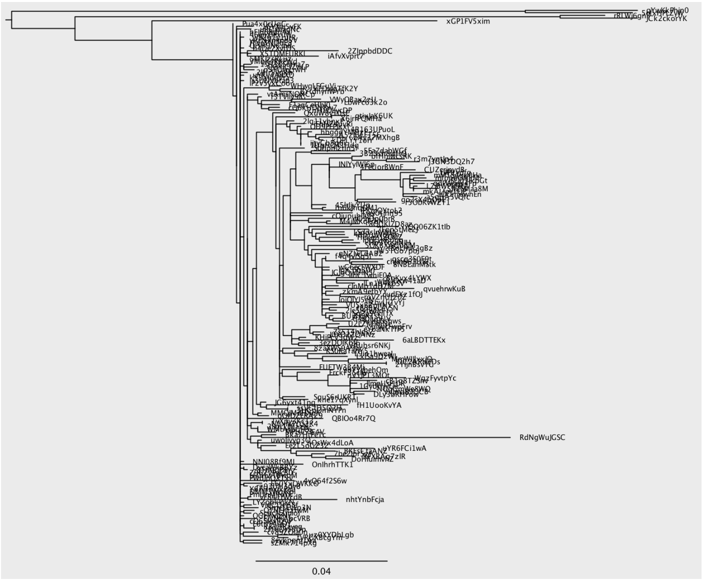

Supplementary Fig. 65 (a) Transition mutation frequencies for the ancestral analysis of Entero C VP2 Polio2 with mean and standard error (black line). (b) Wilcoxon test results, where the shade of the blue in the P-value cell represents the significance level; darker the shade, the more significant the results. (c) Midpoint rooted tree from a sample size of 200.

Ancestral Entero D68 VP1

A

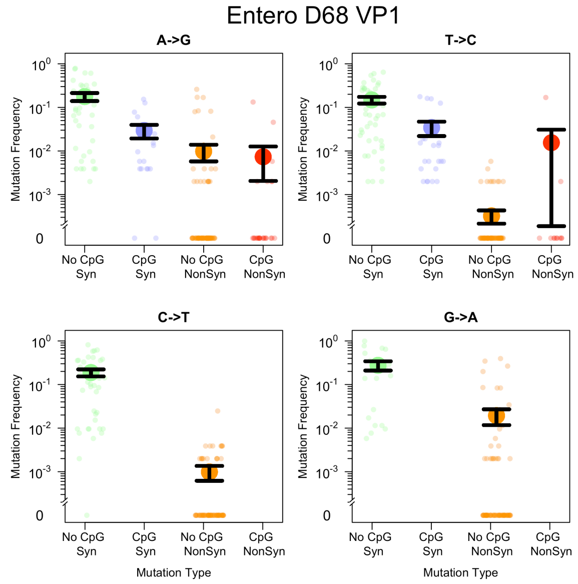

B

Entero D68 VP1

Mutation Type	Comparison	P−Value
A−>G	Syn: CpG v NonCpG	< 0.01
	NonSyn: CpG v NonCpG	0.675
	Syn v NonSyn	< 0.01
T−>C	Syn: CpG v NonCpG	< 0.01
	NonSyn: CpG v NonCpG	0.707
	Syn v NonSyn	< 0.01

C

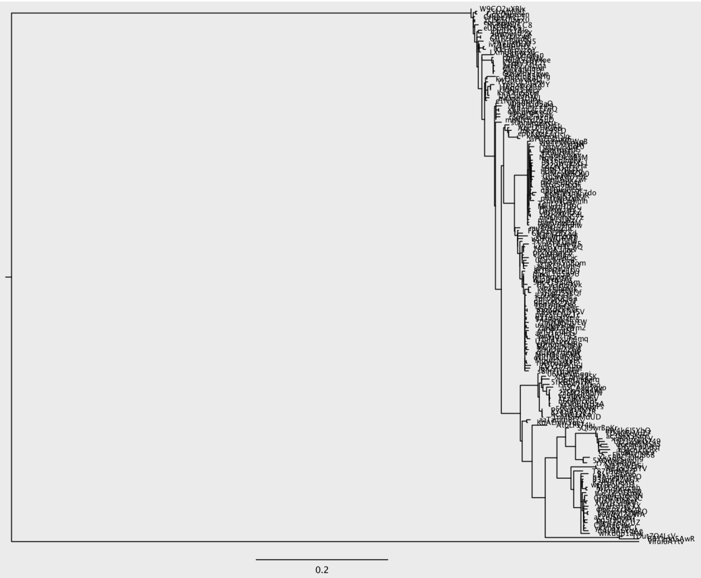

Supplementary Fig. 66 (a) Transition mutation frequencies for the ancestral analysis of Entero D68 VP1 with mean and standard error (black line). (b) Wilcoxon test results, where the shade of the blue in the P-value cell represents the significance level; darker the shade, the more significant the results. (c) Midpoint rooted tree from a sample size of 200.

Ancestral Human Respiratory Syncytial

A

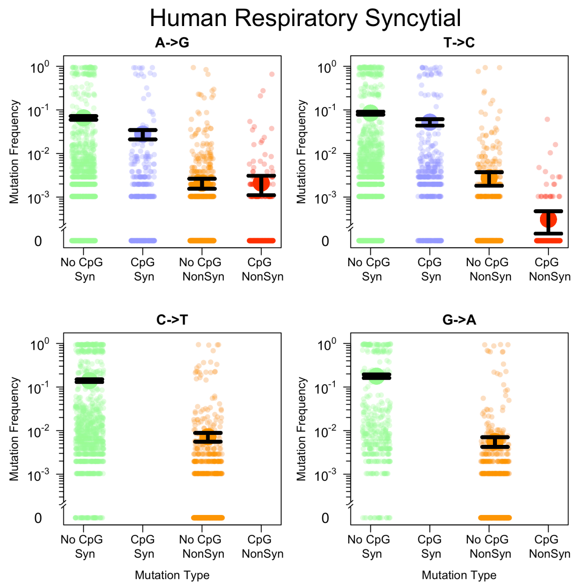

B

Human Respiratory Syncytial

Mutation Type	Comparison	P–Value
A–>G	Syn: CpG v NonCpG	< 0.01
	NonSyn: CpG v NonCpG	0.48
	Syn v NonSyn	< 0.01
T–>C	Syn: CpG v NonCpG	< 0.01
	NonSyn: CpG v NonCpG	< 0.01
	Syn v NonSyn	< 0.01

C

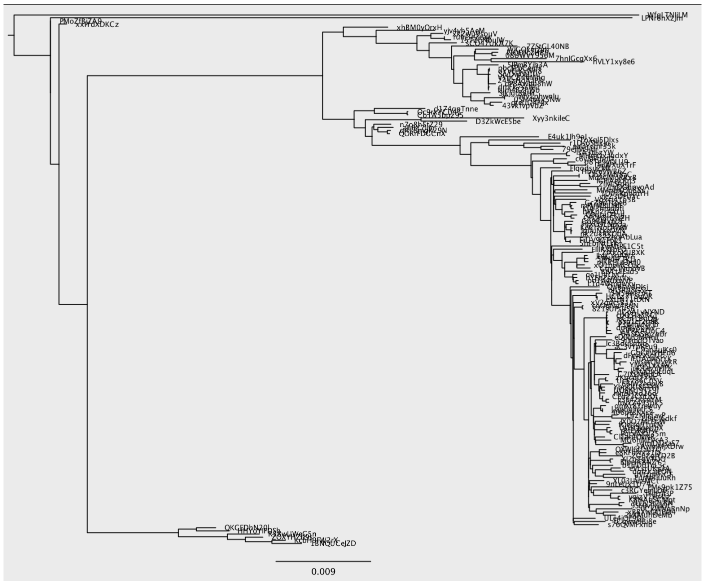

Supplementary Fig. 67 (a) Transition mutation frequencies for the ancestral analysis of Human Respiratory Syncytial with mean and standard error (black line). (b) Wilcoxon test results, where the shade of the blue in the P-value cell represents the significance level; darker the shade, the more significant the results. (c) Midpoint rooted tree from a sample size of 200.

Ancestral Measles HH

A

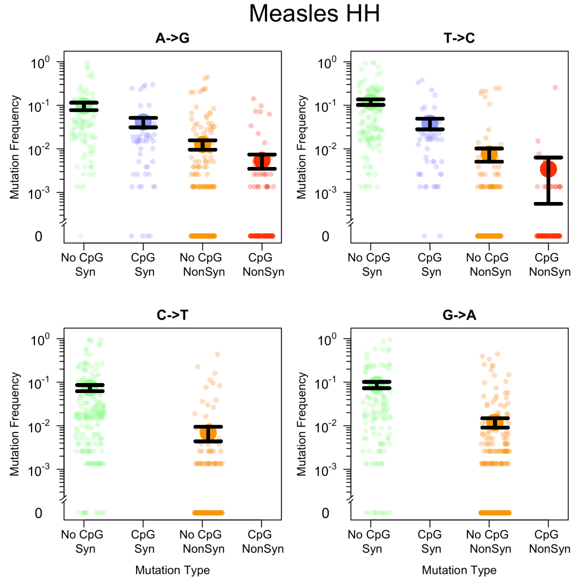

B

Measles HH

Mutation Type	Comparison	P–Value
A–>G	Syn: CpG v NonCpG	< 0.01
	NonSyn: CpG v NonCpG	0.136
	Syn v NonSyn	< 0.01
T–>C	Syn: CpG v NonCpG	< 0.01
	NonSyn: CpG v NonCpG	0.0411
	Syn v NonSyn	< 0.01

C

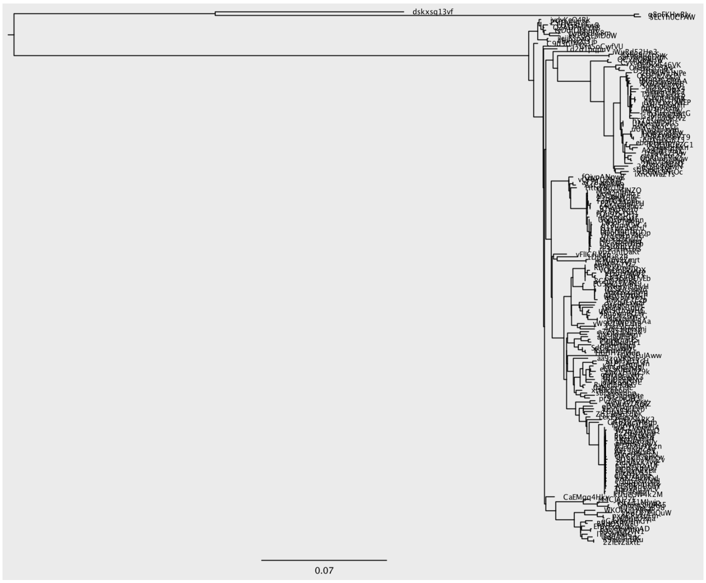

Supplementary Fig. 68 (a) Transition mutation frequencies for the ancestral analysis of Measles HH with mean and standard error (black line). (b) Wilcoxon test results, where the shade of the blue in the P-value cell represents the significance level; darker the shade, the more significant the results. (c) Midpoint rooted tree from a sample size of 200.

Ancestral Rhino B

A

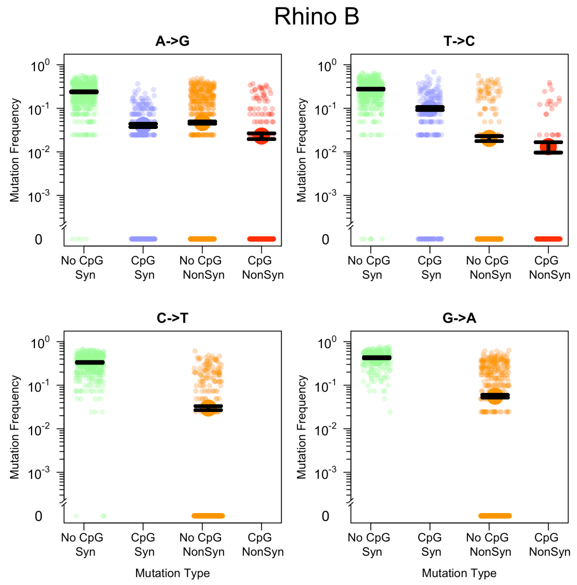

B

Rhino B

Mutation Type	Comparison	P–Value
A–>G	Syn: CpG v NonCpG	< 0.01
	NonSyn: CpG v NonCpG	< 0.01
	Syn v NonSyn	< 0.01
T–>C	Syn: CpG v NonCpG	< 0.01
	NonSyn: CpG v NonCpG	0.41
	Syn v NonSyn	< 0.01

C

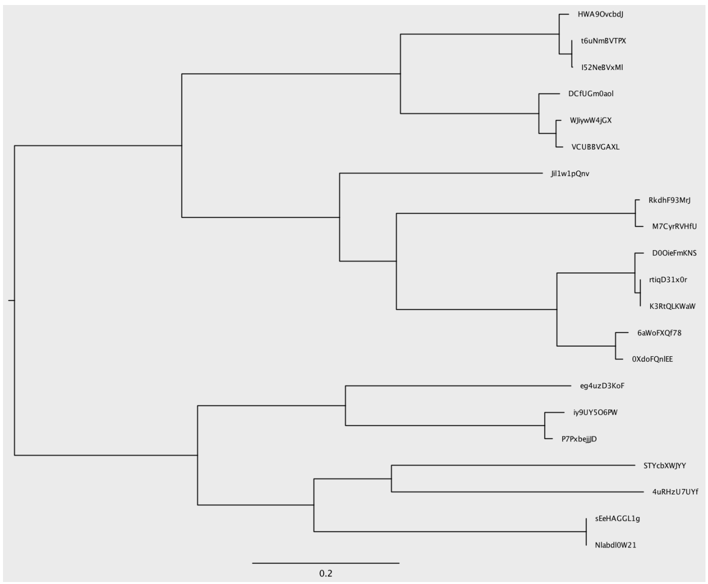

Supplementary Fig. 69 (a) Transition mutation frequencies for the ancestral analysis of Rhino B with mean and standard error (black line). (b) Wilcoxon test results, where the shade of the blue in the P-value cell represents the significance level; darker the shade, the more significant the results. (c) Midpoint rooted tree from a sample size of 200.

Ancestral Rhino C

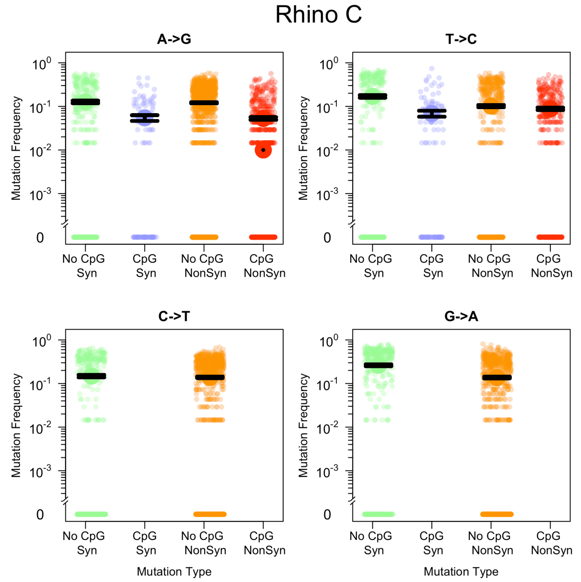

B

Rhino C

Mutation Type	Comparison	P–Value
A–>G	Syn: CpG v NonCpG	< 0.01
	NonSyn: CpG v NonCpG	< 0.01
	Syn v NonSyn	0.798
T–>C	Syn: CpG v NonCpG	0.0269
	NonSyn: CpG v NonCpG	0.998
	Syn v NonSyn	0.0143

C

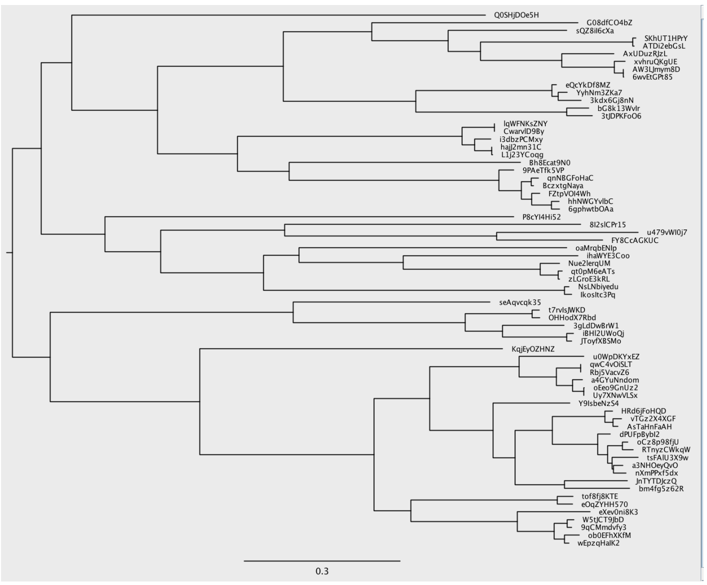

Supplementary Fig. 70 (a) Transition mutation frequencies for the ancestral analysis of Rhino C with mean and standard error (black line). (b) Wilcoxon test results, where the shade of the blue in the P-value cell represents the significance level; darker the shade, the more significant the results. (c) Midpoint rooted tree from a sample size of 200.

Ancestral Rota A VP6

A

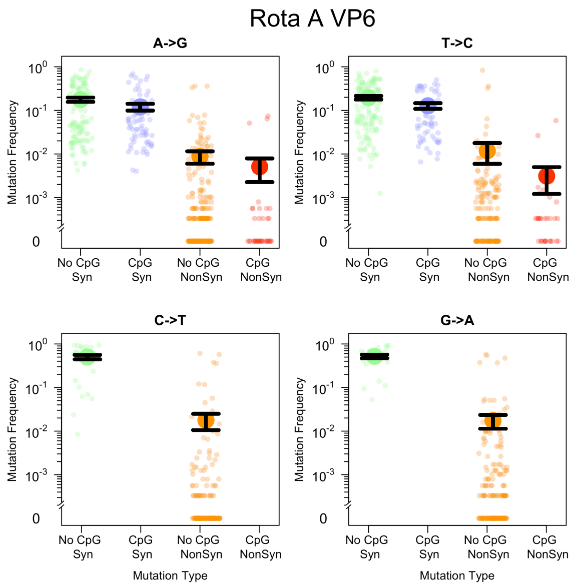

B

Rota A VP6

Mutation Type	Comparison	P−Value
A−>G	Syn: CpG v NonCpG	0.103
	NonSyn: CpG v NonCpG	< 0.01
	Syn v NonSyn	< 0.01
T−>C	Syn: CpG v NonCpG	0.0121
	NonSyn: CpG v NonCpG	0.146
	Syn v NonSyn	< 0.01

C

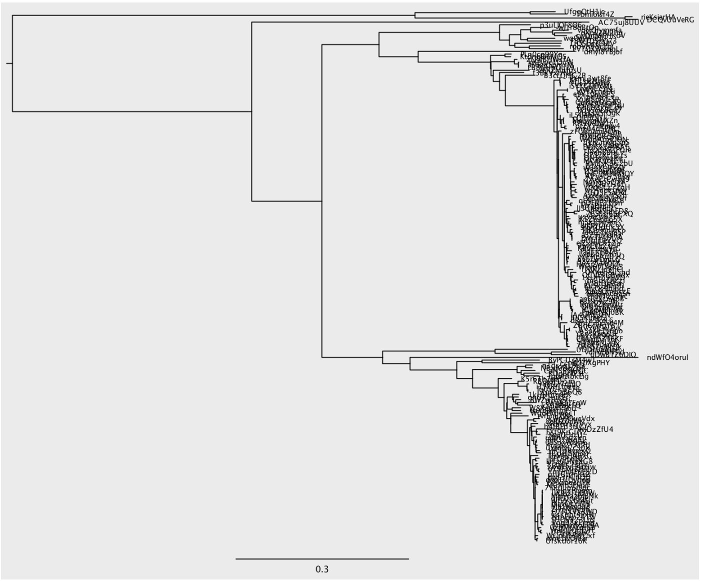

Supplementary Fig. 71 (a) Transition mutation frequencies for the ancestral analysis of Rota A VP6 with mean and standard error (black line). (b) Wilcoxon test results, where the shade of the blue in the P-value cell represents the significance level; darker the shade, the more significant the results. (c) Midpoint rooted tree from a sample size of 200.

Ancestral Bk Polymerase

A

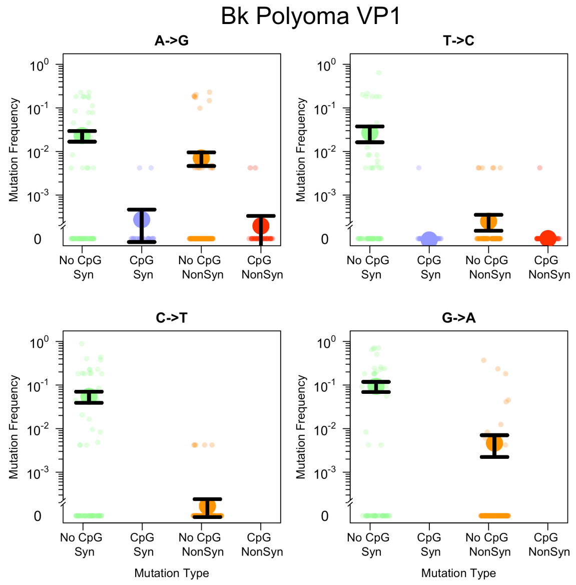

B

Bk Polyoma VP1

Mutation Type	Comparison	P–Value
A–>G	Syn: CpG v NonCpG	< 0.01
	NonSyn: CpG v NonCpG	0.178
	Syn v NonSyn	< 0.01
T–>C	Syn: CpG v NonCpG	< 0.01
	NonSyn: CpG v NonCpG	0.185
	Syn v NonSyn	< 0.01

C

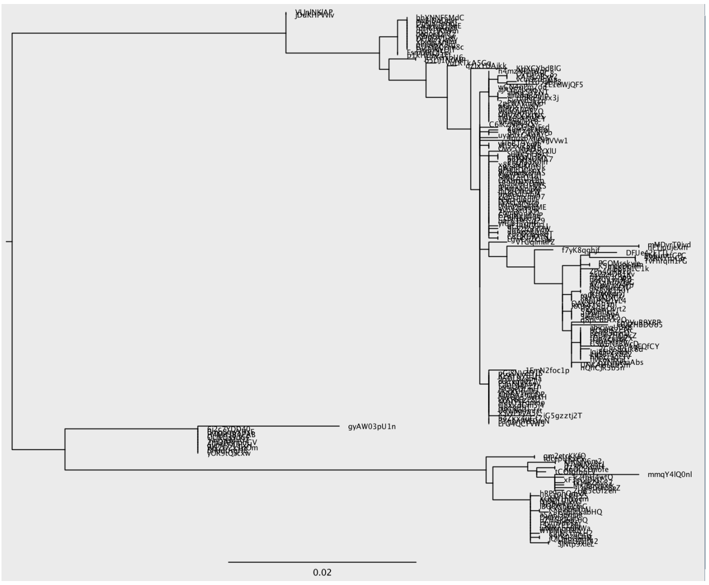

Supplementary Fig. 72 (a) Transition mutation frequencies for the ancestral analysis of Bk Polymerase with mean and standard error (black line). (b) Wilcoxon test results, where the shade of the blue in the P-value cell represents the significance level; darker the shade, the more significant the results. (c) Midpoint rooted tree from a sample size of 200.

Ancestral Human Boca 1 VP1

A

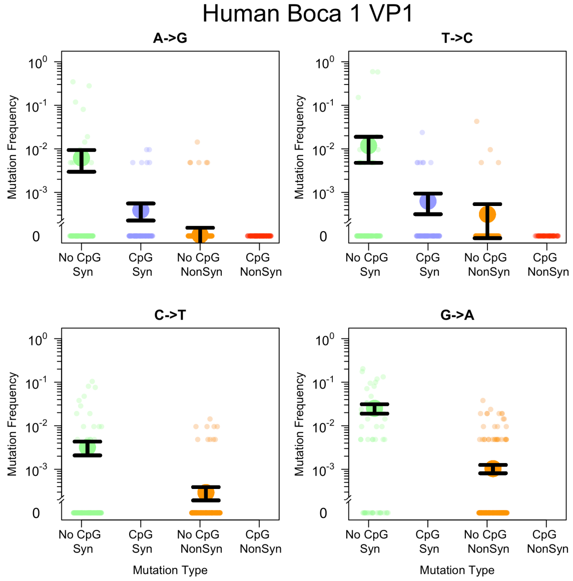

B

Human Boca 1 VP1

Mutation Type	Comparison	P–Value
A–>G	Syn: CpG v NonCpG	0.201
	NonSyn: CpG v NonCpG	0.0854
	Syn v NonSyn	< 0.01
T–>C	Syn: CpG v NonCpG	0.161
	NonSyn: CpG v NonCpG	0.141
	Syn v NonSyn	< 0.01

C

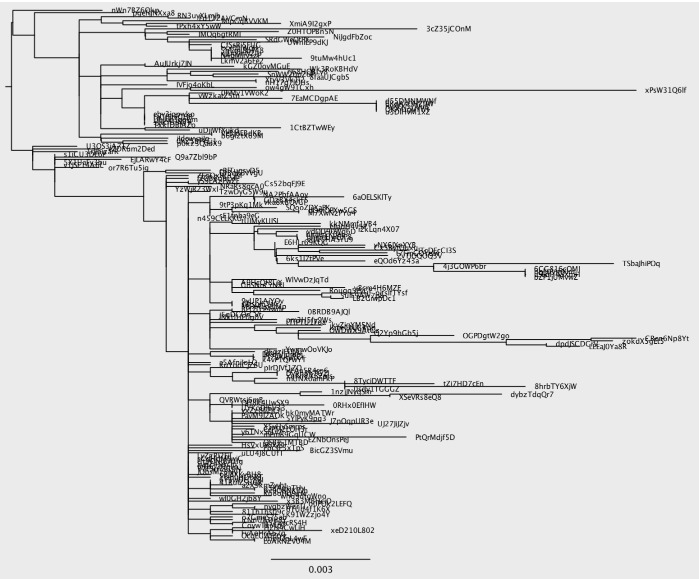

Supplementary Fig. 73 (a) Transition mutation frequencies for the ancestral analysis of Human Boca 1 VP1 with mean and standard error (black line). (b) Wilcoxon test results, where the shade of the blue in the P-value cell represents the significance level; darker the shade, the more significant the results. (c) Midpoint rooted tree from a sample size of 200.

Ancestral HBV A Polymerase

A

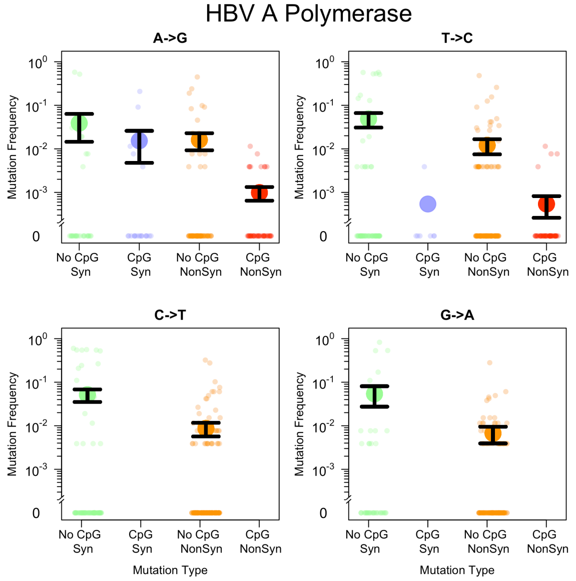

B

HBV A Polymerase

Mutation Type	Comparison	P–Value
A–>G	Syn: CpG v NonCpG	0.321
	NonSyn: CpG v NonCpG	0.282
	Syn v NonSyn	0.129
T–>C	Syn: CpG v NonCpG	0.203
	NonSyn: CpG v NonCpG	< 0.01
	Syn v NonSyn	0.238

C

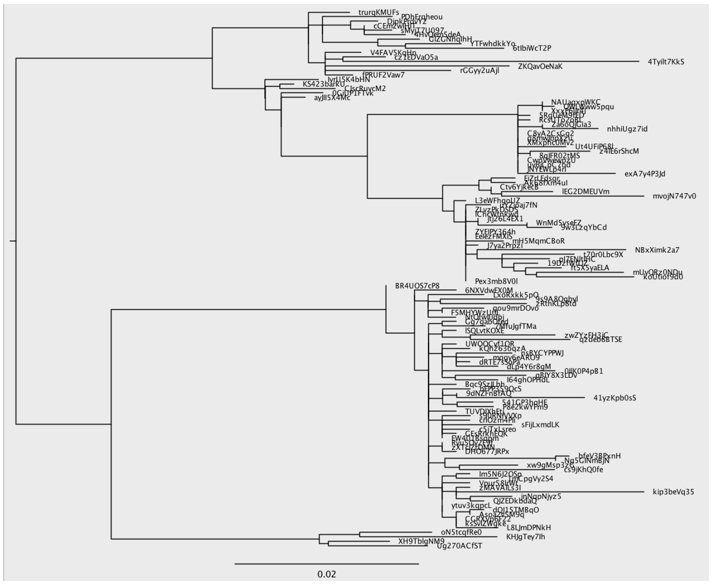

Supplementary Fig. 74 (a) Transition mutation frequencies for the ancestral analysis of HBV A Polymerase with mean and standard error (black line). (b) Wilcoxon test results, where the shade of the blue in the P-value cell represents the significance level; darker the shade, the more significant the results. (c) Midpoint rooted tree from a sample size of 200.

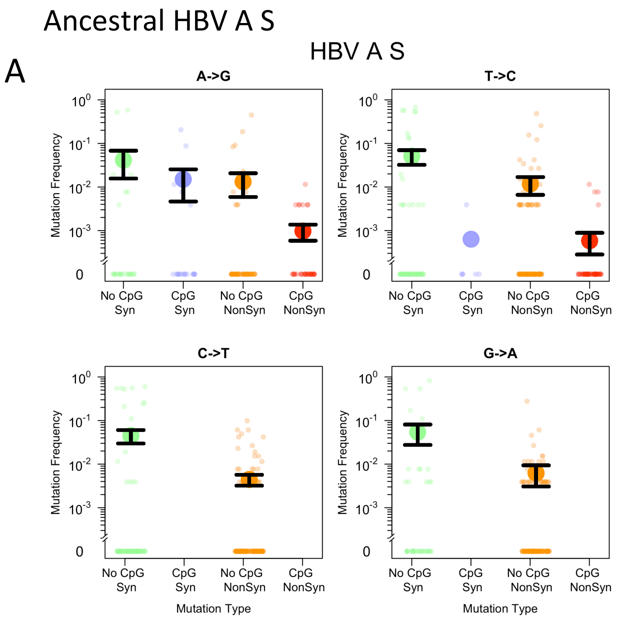

B

HBV A S

Mutation Type	Comparison	P–Value
A–>G	Syn: CpG v NonCpG	0.271
	NonSyn: CpG v NonCpG	0.49
	Syn v NonSyn	0.0602
T–>C	Syn: CpG v NonCpG	0.253
	NonSyn: CpG v NonCpG	< 0.01
	Syn v NonSyn	0.15

C

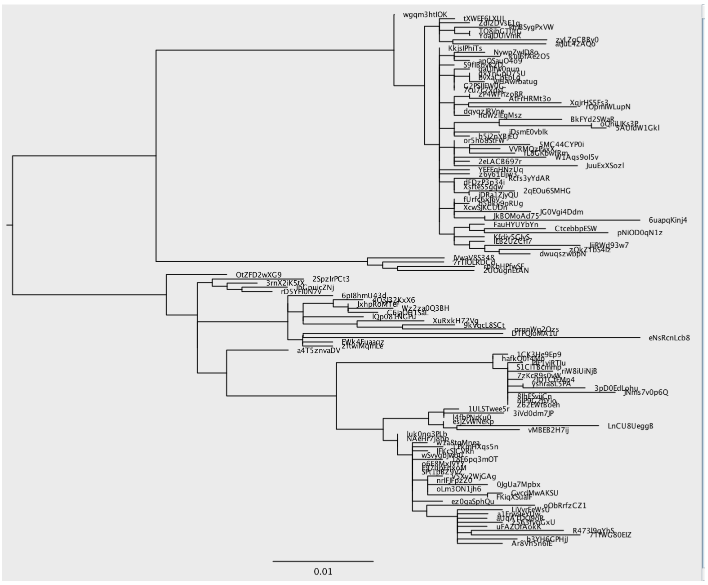

Supplementary Fig. 75 (a) Transition mutation frequencies for the ancestral analysis of HBV A S with mean and standard error (black line). (b) Wilcoxon test results, where the shade of the blue in the P-value cell represents the significance level; darker the shade, the more significant the results. (c) Midpoint rooted tree from a sample size of 200.

Ancestral HBV B Polymerase

A

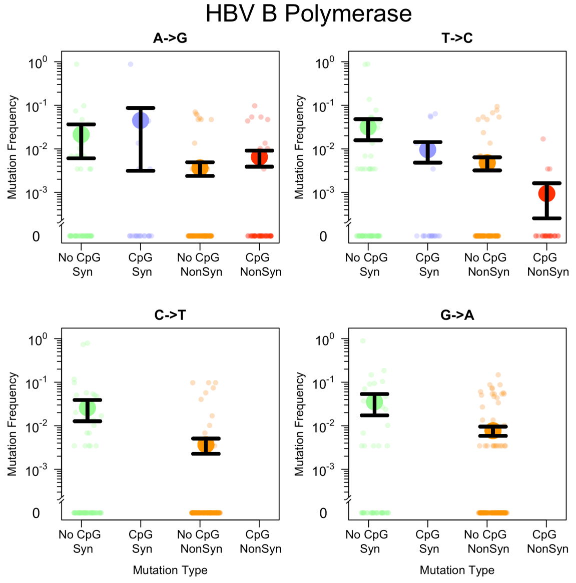

B

HBV B Polymerase

Mutation Type	Comparison	P−Value
A−>G	Syn: CpG v NonCpG	0.326
	NonSyn: CpG v NonCpG	0.895
	Syn v NonSyn	< 0.01
T−>C	Syn: CpG v NonCpG	0.261
	NonSyn: CpG v NonCpG	0.381
	Syn v NonSyn	< 0.01

C

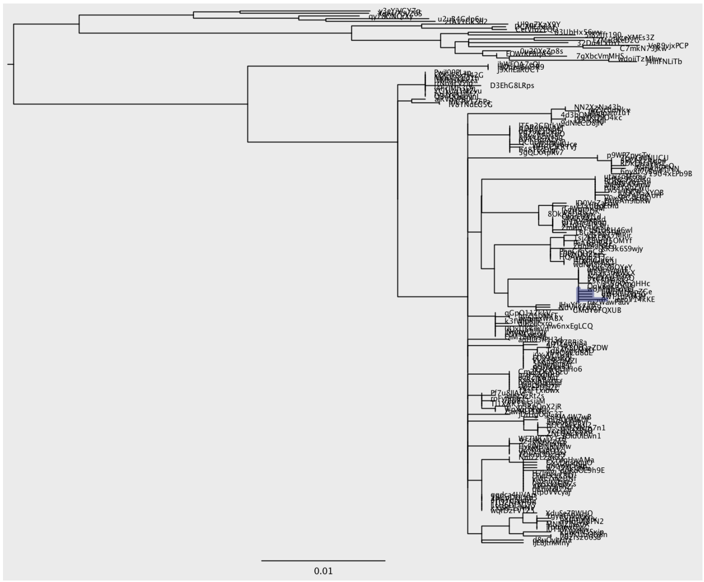

Supplementary Fig. 76 (a) Transition mutation frequencies for the ancestral analysis of HBV B Polymerase with mean and standard error (black line). (b) Wilcoxon test results, where the shade of the blue in the P-value cell represents the significance level; darker the shade, the more significant the results. (c) Midpoint rooted tree from a sample size of 200.

Ancestral HBV B PreC-Core

A

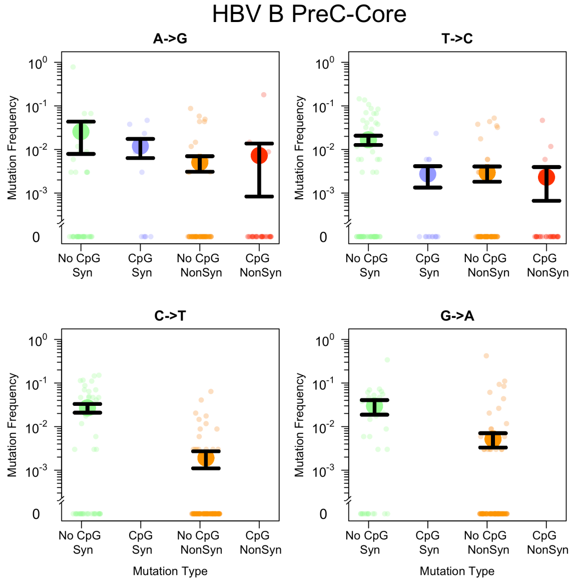

B

HBV B PreC–Core

Mutation Type	Comparison	P–Value
A–>G	Syn: CpG v NonCpG	0.788
	NonSyn: CpG v NonCpG	0.199
	Syn v NonSyn	< 0.01
T–>C	Syn: CpG v NonCpG	0.204
	NonSyn: CpG v NonCpG	0.559
	Syn v NonSyn	< 0.01

C

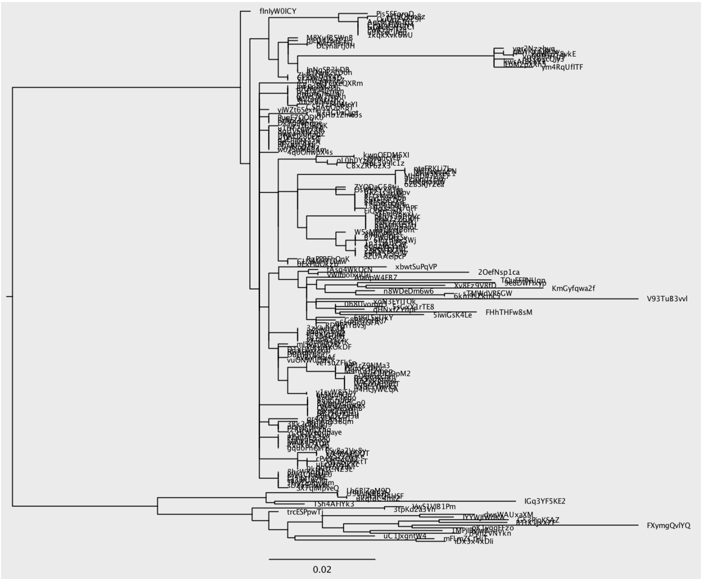

Supplementary Fig. 77 (a) Transition mutation frequencies for the ancestral analysis of HBV B PreC-Core with mean and standard error (black line). (b) Wilcoxon test results, where the shade of the blue in the P-value cell represents the significance level; darker the shade, the more significant the results. (c) Midpoint rooted tree from a sample size of 200.

Ancestral HBV C Polymerase

A

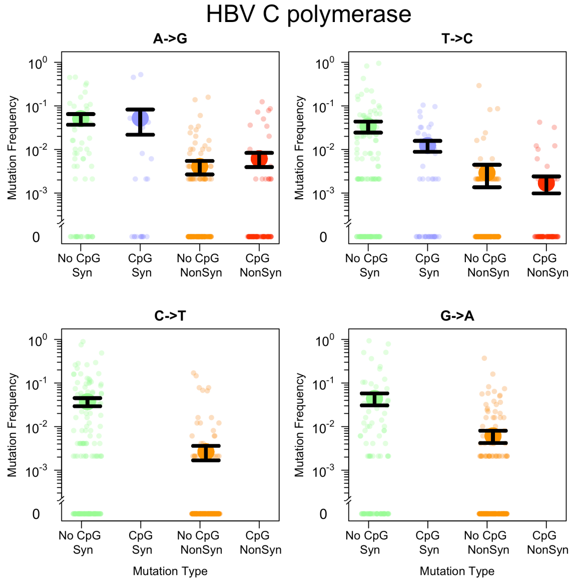

B

HBV C polymerase

Mutation Type	Comparison	P–Value
A–>G	Syn: CpG v NonCpG	0.108
	NonSyn: CpG v NonCpG	0.209
	Syn v NonSyn	< 0.01
T–>C	Syn: CpG v NonCpG	0.156
	NonSyn: CpG v NonCpG	0.542
	Syn v NonSyn	< 0.01

C

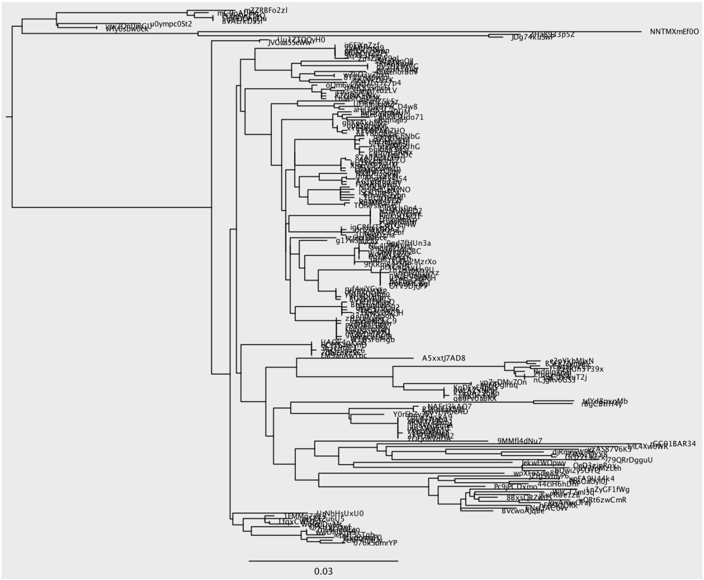

Supplementary Fig. 78 (a) Transition mutation frequencies for the ancestral analysis of HBV C Polymerase with mean and standard error (black line). (b) Wilcoxon test results, where the shade of the blue in the P-value cell represents the significance level; darker the shade, the more significant the results. (c) Midpoint rooted tree from a sample size of 200.

Ancestral HBV C PreC-Core

A

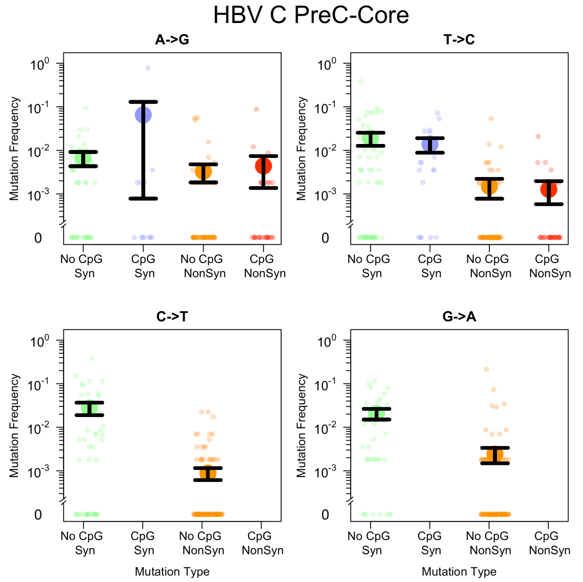

B

HBV C PreC–Core

Mutation Type	Comparison	P–Value
A–>G	Syn: CpG v NonCpG	0.132
	NonSyn: CpG v NonCpG	0.876
	Syn v NonSyn	< 0.01
T–>C	Syn: CpG v NonCpG	0.788
	NonSyn: CpG v NonCpG	0.599
	Syn v NonSyn	< 0.01

C

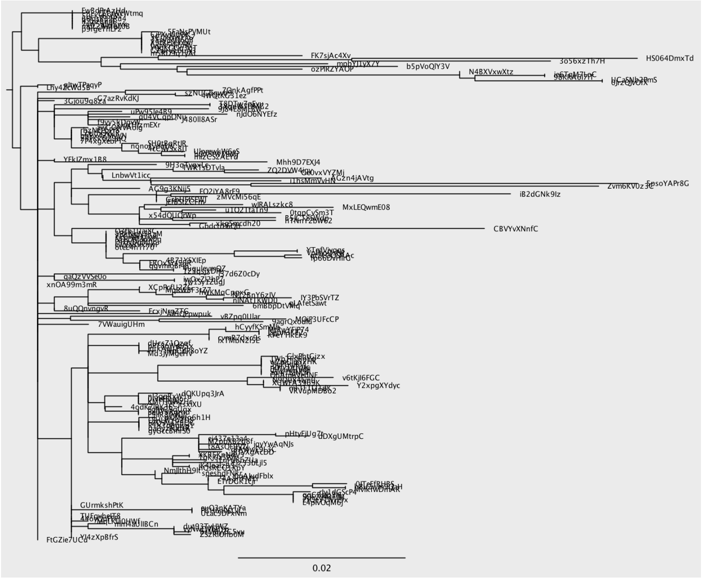

Supplementary Fig. 79 (a) Transition mutation frequencies for the ancestral analysis of HBV C PreC-Core with mean and standard error (black line). (b) Wilcoxon test results, where the shade of the blue in the P-value cell represents the significance level; darker the shade, the more significant the results. (c) Midpoint rooted tree from a sample size of 200.

Ancestral HBV C S

A

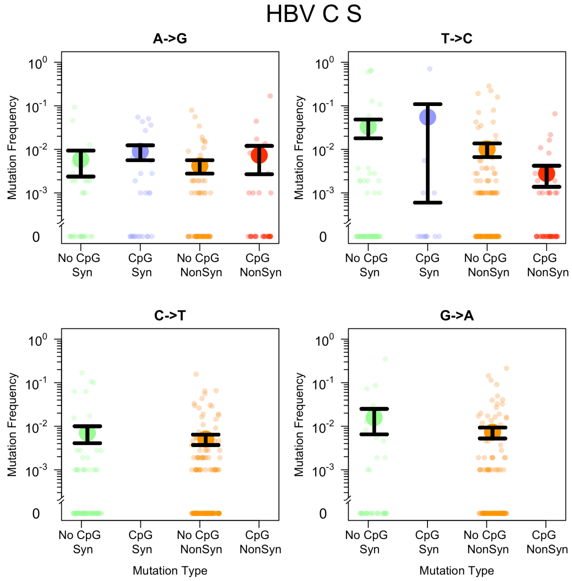

B

HBV C S

Mutation Type	Comparison	P–Value
A–>G	Syn: CpG v NonCpG	0.813
	NonSyn: CpG v NonCpG	0.173
	Syn v NonSyn	0.134
T–>C	Syn: CpG v NonCpG	0.411
	NonSyn: CpG v NonCpG	0.141
	Syn v NonSyn	0.723

C

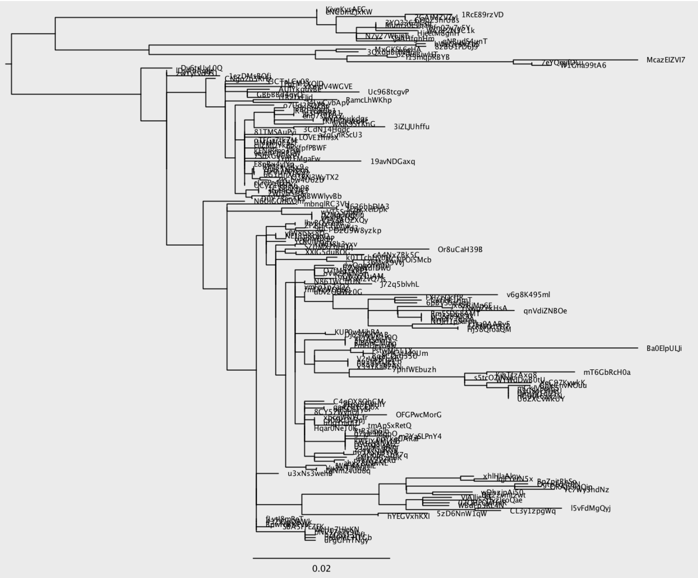

Supplementary Fig. 80(a) Transition mutation frequencies for the ancestral analysis of HBV C S with mean and standard error (black line). (b) Wilcoxon test results, where the shade of the blue in the P-value cell represents the significance level; darker the shade, the more significant the results. (c) Midpoint rooted tree from a sample size of 200.

Ancestral Herpes 2 Glycoprotein G

A

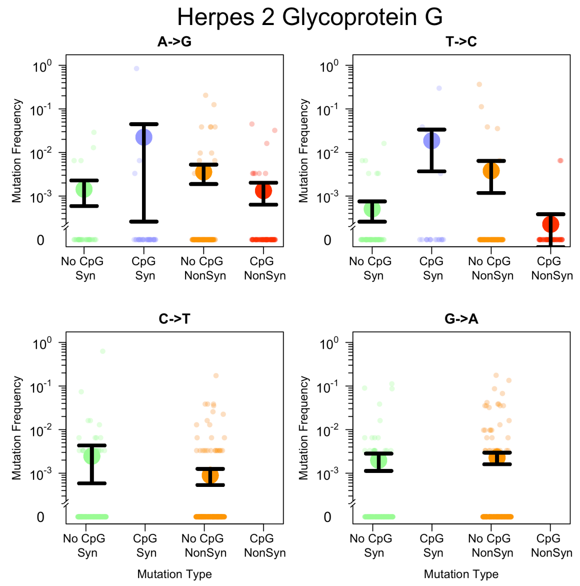

B

Herpes 2 Glycoprotein G

Mutation Type	Comparison	P–Value
A–>G	Syn: CpG v NonCpG	0.343
	NonSyn: CpG v NonCpG	0.501
	Syn v NonSyn	0.529
T–>C	Syn: CpG v NonCpG	0.956
	NonSyn: CpG v NonCpG	0.503
	Syn v NonSyn	< 0.01

C

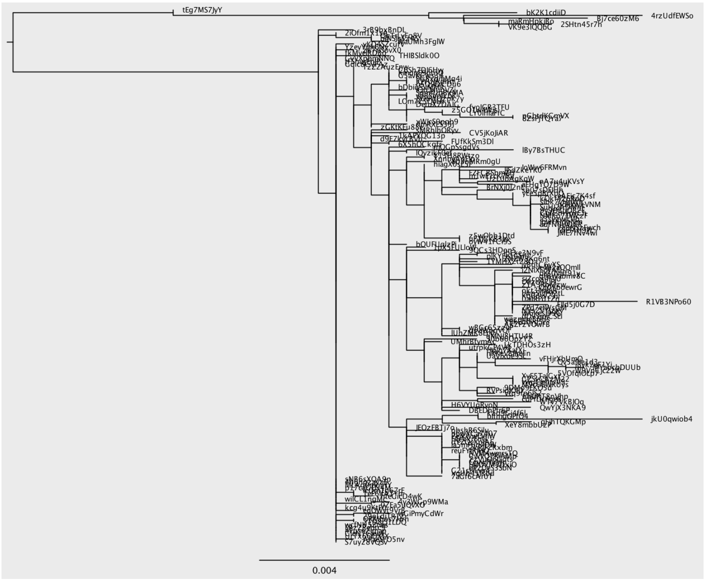

Supplementary Fig. 81 (a) Transition mutation frequencies for the ancestral analysis of Herpes 2 Glycoprotein G with mean and standard error (black line). (b) Wilcoxon test results, where the shade of the blue in the P-value cell represents the significance level; darker the shade, the more significant the results. (c) Midpoint rooted tree from a sample size of 200.

Ancestral Human Papilloma 16 L1

A

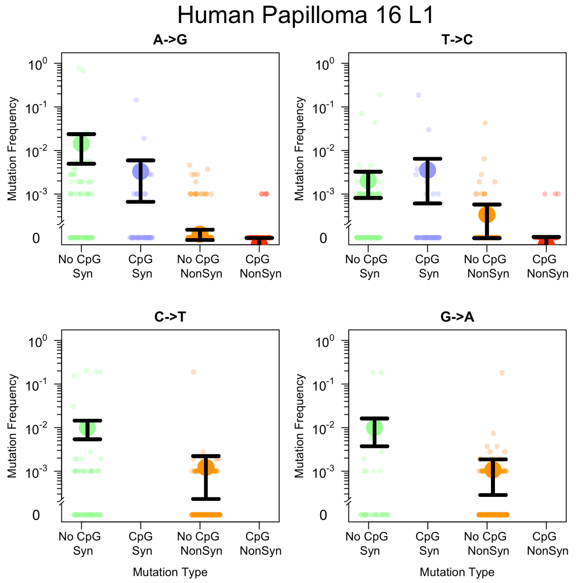

B

Human Papilloma 16 L1

Mutation Type	Comparison	P−Value
A−>G	Syn: CpG v NonCpG	0.0665
	NonSyn: CpG v NonCpG	0.456
	Syn v NonSyn	< 0.01
T−>C	Syn: CpG v NonCpG	0.102
	NonSyn: CpG v NonCpG	0.595
	Syn v NonSyn	< 0.01

C

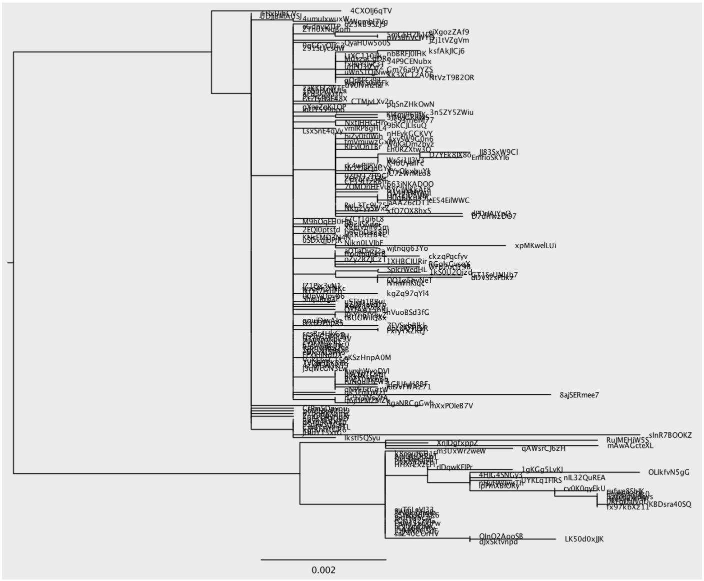

Supplementary Fig. 82 (a) Transition mutation frequencies for the ancestral analysis of Human Papilloma 16 L1 with mean and standard error (black line). (b) Wilcoxon test results, where the shade of the blue in the P-value cell represents the significance level; darker the shade, the more significant the results. (c) Midpoint rooted tree from a sample size of 200.

Ancestral Parvo B19 NS1

A

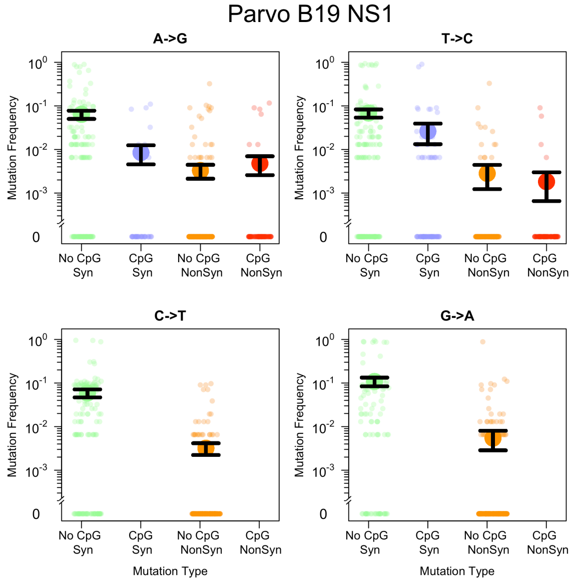

B

Parvo B19 NS1

Mutation Type	Comparison	P–Value
A–>G	Syn: CpG v NonCpG	< 0.01
	NonSyn: CpG v NonCpG	0.743
	Syn v NonSyn	< 0.01
T–>C	Syn: CpG v NonCpG	< 0.01
	NonSyn: CpG v NonCpG	0.287
	Syn v NonSyn	< 0.01

C

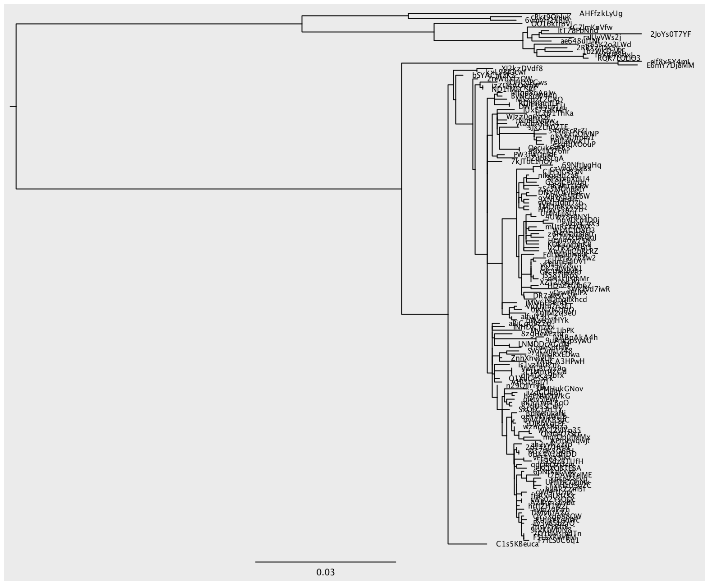

Supplementary Fig. 83 (a) Transition mutation frequencies for the ancestral analysis of Parvo B19 NS1 with mean and standard error (black line). (b) Wilcoxon test results, where the shade of the blue in the P-value cell represents the significance level; darker the shade, the more significant the results. (c) Midpoint rooted tree from a sample size of 200.

Ancestral Parvo B19 VP1

A

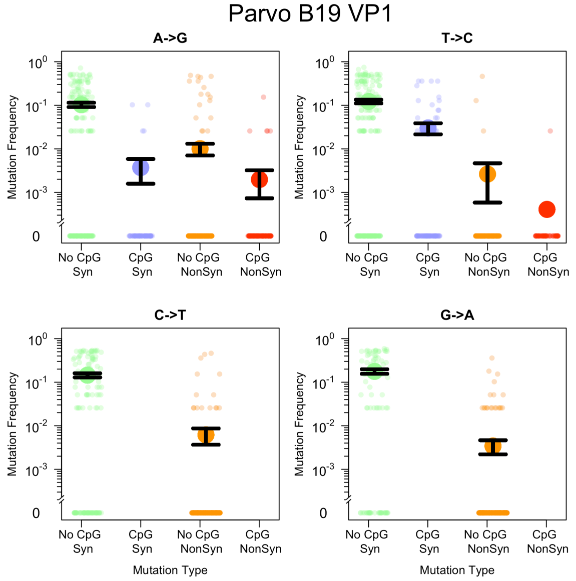

B

Parvo B19 VP1

Mutation Type	Comparison	P–Value
A–>G	Syn: CpG v NonCpG	< 0.01
	NonSyn: CpG v NonCpG	0.343
	Syn v NonSyn	< 0.01
T–>C	Syn: CpG v NonCpG	< 0.01
	NonSyn: CpG v NonCpG	0.572
	Syn v NonSyn	< 0.01

C

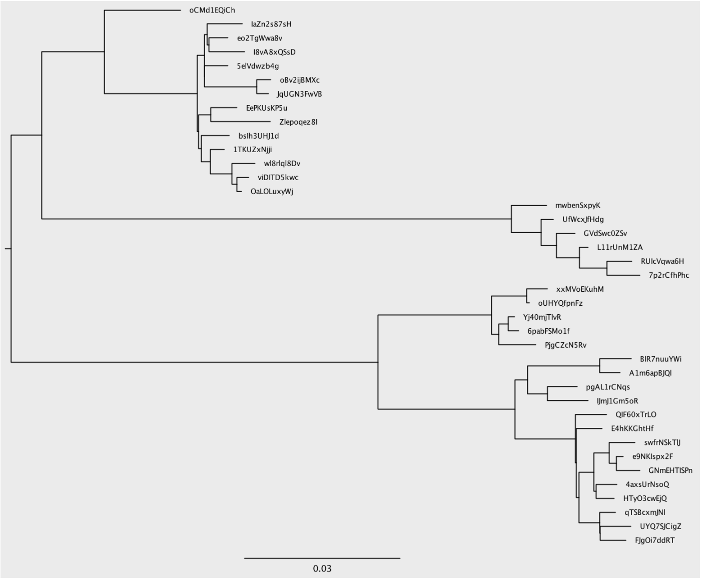

Supplementary Fig. 84 (a) Transition mutation frequencies for the ancestral analysis of Parvo B19 VP1 with mean and standard error (black line). (b) Wilcoxon test results, where the shade of the blue in the P-value cell represents the significance level; darker the shade, the more significant the results. (c) Midpoint rooted tree from a sample size of 200.
