# Supplementary material for: CpG-creating mutations are costly in many human viruses
Source: Evol Ecol. 2020 Apr 24;34(3):339–59. doi: 10.1007/s10682-020-10039-z (PMC7245597; doi:10.1007/s10682-020-10039-z)
Supplement: Supplementary file 2 — Supplementary material 2 (pdf 634 KB) [file 10682_2020_10039_MOESM2_ESM.pdf]

CpG-creating Mutations are Costly in Many Human Viruses – Supplementary Figures

Caudill, Qin, Winstead, Kaur et al.

February 26, 2020

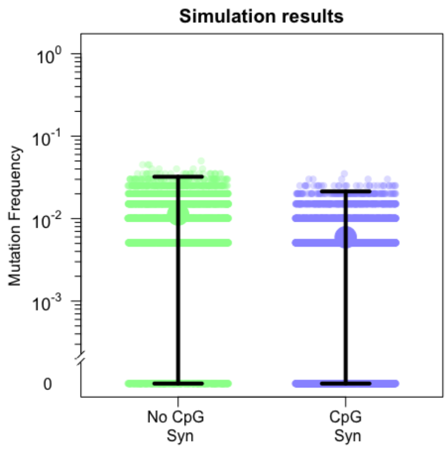

Figure S1. Simulated mutation frequencies (with means (large dots) and standard errors) using the SLIM simulation framework (Haller and Messer, 2019

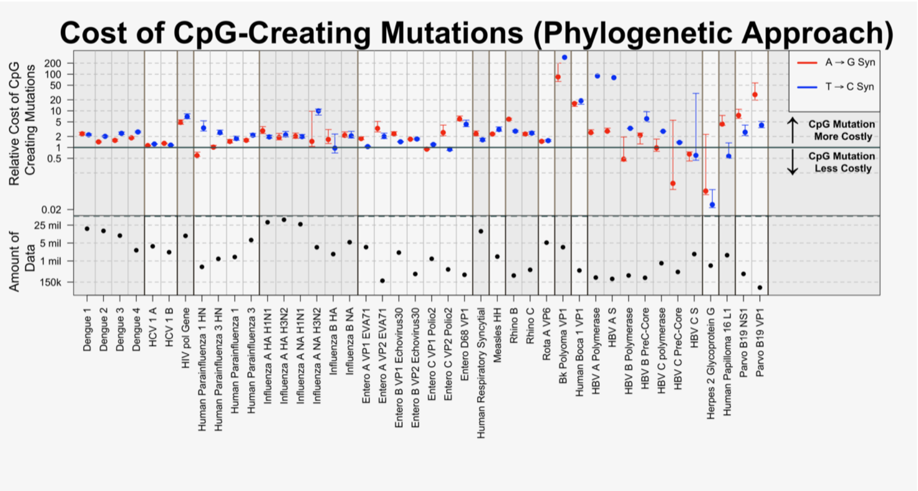

Figure S2. Overview of the cost associated with CpG-creating mutations based on phylogenetic approach. Each dot represents a ratio of the average virus mutation frequency of non-CpG-creating mutations to the average frequency of CpG-creating mutations. The bottom half of the figure depicts the total amount of data in each virus data set(the number of sequences × the number of nucleotides)

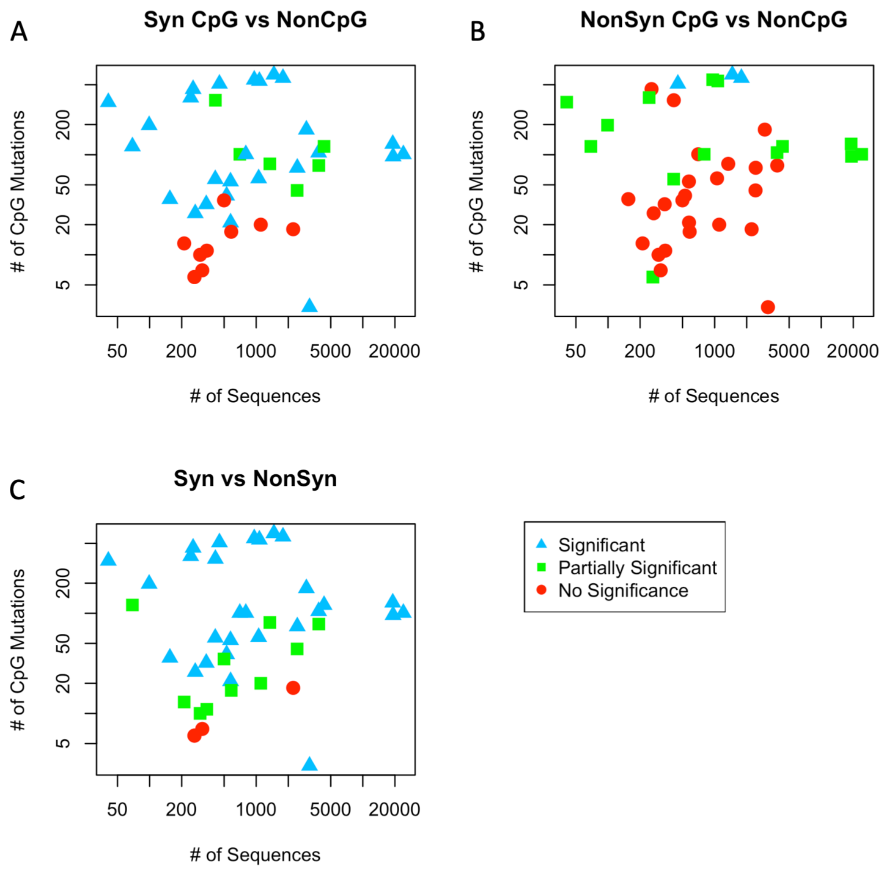

Figure S3. Results based on phylogenetic approach. Each point represents one dataset. Its location corresponds to the amount of sequences (on the x-axis) and the number of sites with CpG-creating mutations (on the y-axis) for each data set. The colors and shapes represent what was found significant in each Wilcoxon test; blue triangles if both A→G and T→C are significant, green squares if only one was significant and red circles if both are not significant. We find that, in general, we are more likely to find significant effects for viruses for which we have more data (towards the top and the right).

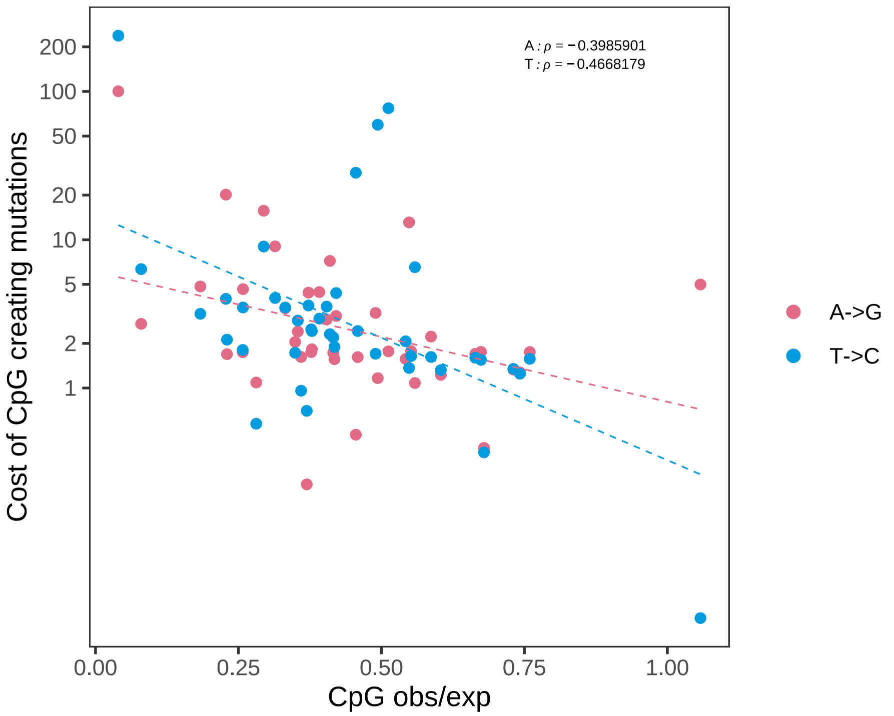

Figure S4. Relation between cost and genomic CpG under-representation. The relationships between costs of CpG creating mutations and the degrees of CG dinucleotide under/over-representation (Rho statistic values) were assessed for all viral genes/genomes used in our study. The results showed overall significant negative correlation (Spearman's $\rho = -0.37$, $P = 0.0005$), indicating the higher the costs of CpG creating mutations, the more CG dinucleotide was underrepresented . Correlation was also assessed separately for A→G and T→C mutations, which resulted in significant negative correlation for T→C mutations (Spearman's $\rho = -0.43$, $P = 0.004$), and marginally significant correlation for A→G mutations (Spearman's $\rho = -0.29$, $P = 0.06$).
